# A *Brucella* effector modulates the Arf6‐Rab8a GTPase cascade to promote intravacuolar replication

**DOI:** 10.15252/embj.2021107664

**Published:** 2021-08-23

**Authors:** Elizabeth Borghesan, Erin P Smith, Sebenzile Myeni, Kelsey Binder, Leigh A Knodler, Jean Celli

**Affiliations:** ^1^ Paul G. Allen School for Global Health Washington State University Pullman WA USA; ^2^ Rocky Mountain Laboratories National Institute of Allergy and Infectious Diseases National Institutes of Health Hamilton MT USA; ^3^ Present address: Department of Molecular Genetics and Microbiology Duke University School of Medicine Durham NC USA; ^4^ Present address: Department of Medical Microbiology Leiden University Medical Center Leiden The Netherlands

**Keywords:** *Brucella*, ACAP1, pathogenesis, retrograde membrane transport, type IV secretion, Membranes & Trafficking, Microbiology, Virology & Host Pathogen Interaction

## Abstract

Remodeling of host cellular membrane transport pathways is a common pathogenic trait of many intracellular microbes that is essential to their intravacuolar life cycle and proliferation. The bacterium *Brucella abortus* generates a host endoplasmic reticulum‐derived vacuole (rBCV) that supports its intracellular growth, via VirB Type IV secretion system‐mediated delivery of effector proteins, whose functions and mode of action are mostly unknown. Here, we show that the effector BspF specifically promotes *Brucella* replication within rBCVs by interfering with vesicular transport between the *trans*‐Golgi network (TGN) and recycling endocytic compartment. BspF targeted the recycling endosome, inhibited retrograde traffic to the TGN, and interacted with the Arf6 GTPase‐activating Protein (GAP) ACAP1 to dysregulate Arf6‐/Rab8a‐dependent transport within the recycling endosome, which resulted in accretion of TGN‐associated vesicles by rBCVs and enhanced bacterial growth. Altogether, these findings provide mechanistic insight into bacterial modulation of membrane transport used to promote their own proliferation within intracellular vacuoles.

## Graphical abstract

*Brucella abortus* effector protein BspF binding to host GTPase‐activating protein ACAP1 facilitates intracellular bacterial growth by decreasing retrograde vesicular transport to the trans‐Golgi network.

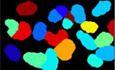

## Introduction

Microbial pathogens with an intracellular lifestyle have developed sophisticated strategies to promote their proliferation within host cells, by exploiting various host cellular processes. Among these strategies, pathogen‐driven remodeling of membrane trafficking pathways either mediates biogenesis of pathogen‐containing vacuoles or provides replication‐permissive conditions within these vacuoles, through nutrient delivery or accretion of host membranes that supports vacuolar integrity and expansion. Vesicle trafficking pathways between the secretory and endosomal compartments are common targets of pathogen manipulation. Anterograde vesicular traffic along the secretory pathway delivers protein cargo and lipids to their destination compartment, providing organelle identity and function. Retrograde transport includes various vesicular trafficking pathways operating between early, recycling, and late endosomal compartments and the *trans*‐Golgi network (TGN) and ER, recycling membrane‐associated trafficking components to their compartments of origin (Bonifacino & Rojas, 2006; Johannes & Wunder, 2011). While retrograde transport between the endosomal and secretory compartments is co‐opted by several bacterial toxins to reach their intracellular targets (Sandvig *et al*, 2013), it is also involved in the intracellular cycle of some bacterial pathogens (Personnic *et al*, 2016; Allgood & Neunuebel, 2018). For example, *Salmonella enterica* serovar Typhimurium (*S*. Typhimurium) inhibits Rab9‐dependent retrograde transport to promote survival within the *Salmonella*‐containing vacuole (SCV) (McGourty *et al*, 2012) and also modulates function of the retromer coat complex, which coordinates endosomal sorting in retrograde transport (Burd & Cullen, 2014), to promote SCV integrity (Patrick *et al*, 2018). The retromer also either mediates growth restriction of *Chlamydia trachomatis* and *Legionella pneumophila* (Finsel *et al*, 2013; Mirrashidi *et al*, 2015) or is required for biogenesis of the *Coxiella burnetii*‐containing vacuole (McDonough *et al*, 2013) and the *Brucella abortus* replicative vacuole (Casanova *et al*, 2019), suggesting pathogen‐restricting and pathogen‐promoting roles of endosome‐to‐Golgi retrograde transport.

The bacterium *Brucella abortus*, a causative agent of the world‐wide zoonosis brucellosis (Pappas *et al*, 2006), undergoes a complex intracellular cycle in phagocytes that includes sequential interactions of its membrane‐bound vacuole, the *Brucella*‐containing vacuole (BCV), with the endocytic, secretory, and autophagic pathways (Celli, 2019). Upon phagocytic uptake, the nascent BCV traffics along the endocytic pathway and partially fuses with lysosomes to become an acidified, endosomal BCV (eBCV), a maturation process that triggers expression of the VirB Type IV secretion system (T4SS) (Boschiroli, 2002; Sieira *et al*, 2004; Starr *et al*, 2008). VirB T4SS‐mediated delivery of effector proteins (de Jong *et al*, 2008; de Barsy *et al*, 2011; Ines Marchesini *et al*, 2011; Döhmer *et al*, 2013; Myeni *et al*, 2013) mediates BCV interactions with the ER and Golgi compartments, culminating in the biogenesis of an ER‐derived, replication‐permissive vacuole (rBCV) (Pizarro‐Cerdá *et al*, 1998; Comerci *et al*, 2001; Celli *et al*, 2003, 2005; Miller *et al*, 2017). Following extensive bacterial replication in rBCVs, autophagic capture of rBCVs and their conversion into fusogenic aBCVs (autophagic BCVs) leads to bacterial egress (Starr *et al*, 2012). While several VirB T4SS effectors have been identified (de Jong *et al*, 2008; de Barsy *et al*, 2011; Ines Marchesini *et al*, 2011; Döhmer *et al*, 2013; Myeni *et al*, 2013), the functions of most are unknown. RicA and BspB contribute to rBCV biogenesis via interference with Rab2a‐dependent and COG complex‐mediated retrograde traffic between the Golgi and ER (de Barsy *et al*, 2011; Miller *et al*, 2017; Smith *et al*, 2020), providing some molecular insight into *Brucella’s* exploitation of ER‐Golgi secretory transport for the purpose of rBCV biogenesis. Recent evidence indicates that VirB T4SS activity is also required for replication within rBCVs and aBCV formation (Smith *et al*, 2016), yet no effector proteins associated with these later stages have been identified or characterized. Here, we show that the T4SS effector BspF specifically promotes *Brucella* growth within rBCVs by interacting with the GTPase‐activating protein (GAP) ACAP1 to modulate Arf6 activity on recycling endosomal membranes. BspF–ACAP1 interaction specifically inhibits the Arf6/Rab8a regulatory axis of membrane transport between the recycling endosome and the TGN to cause recruitment of TGN‐derived vesicles to rBCVs. Hence, these findings reveal *Brucella*’s modulation of a specific recycling transport pathway for the purpose of intravacuolar growth via T4SS effector‐mediated interference with a GTPase regulatory cascade.

## Results

### BspF is required for *Brucella* replication within rBCVs

**Figure 1:**
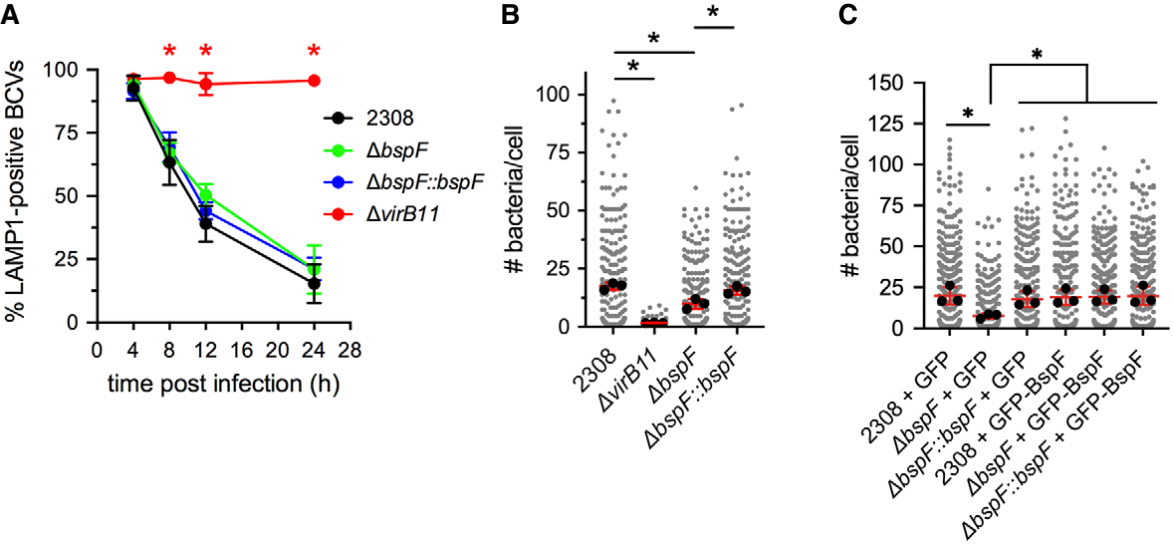
BspF is required for *Brucella* replication within rBCVs rBCV biogenesis in BMMs infected with either wild‐type (2308), VirB‐deficient (Δ*virB11*), Δ*bspF*, or complemented ∆*bspF* (Δ*bspF::bspF*) bacteria, measured as the percentage of LAMP1‐positive BCVs over time. Data are means ± SD of *n* = 3 independent experiments. Asterisks indicate statistically significant differences (*P* < 0.05, two‐way ANOVA followed by Dunnett’s multiple comparisons test) compared with control (2308).*Brucella* replication in BMMs infected with either wild‐type (2308), VirB‐deficient (Δ*virB11*), Δ*bspF*, or complemented ∆*bspF* (Δ*bspF::bspF*) bacteria, measured as number of bacteria per cell at 24 h pi. Data are means ± SD of *n* = 3 independent experiments. Gray dots represent values from individual cells analyzed (*n* > 300); black dots indicate means of individual experiments. Asterisks indicate statistically significant differences (*P* < 0.05, one‐way ANOVA followed by Dunnett’s multiple comparisons test) compared with control (2308).*Brucella* replication in BMMs expressing either GFP or GFP‐BspF and infected with either wild‐type (2308), Δ*bspF*, or complemented ∆*bspF* (Δ*bspF::bspF*) bacteria, measured as number of bacteria per cell at 24 h pi. Data are means ± SD of *n* = 3 independent experiments. Gray dots represent values from individual cells analyzed (*n* > 300); black dots indicate means of individual experiments. Asterisks indicate statistically significant differences (*P* < 0.05, two‐way ANOVA followed by Dunnett’s multiple comparisons test) compared with controls.

We previously identified BspF as a VirB T4SS‐delivered protein during infection of macrophages that interfered with anterograde secretory traffic when ectopically expressed or during infection of HeLa cells (Myeni *et al*, 2013). To investigate whether BspF plays a role in the intracellular cycle of *B. abortus*, we first examined the ability of an in‐frame ∆*bspF* deletion mutant (Myeni *et al*, 2013) to generate rBCVs in murine bone marrow‐derived macrophages (BMMs), by monitoring the progressive exclusion of endosomal membranes from BCVs over a 24‐h time course as a readout of eBCV to rBCV conversion (Comerci *et al*, 2001; Celli *et al*, 2003; Salcedo *et al*, 2008; Starr *et al*, 2008, 2012; Miller *et al*, 2017; Smith *et al*, 2020). While a ∆*virB11* T4SS‐deficient mutant failed to exclude LAMP1 (Comerci *et al*, 2001; Celli *et al*, 2003) and remained within eBCVs, wild‐type, ∆*bspF*, and complemented ∆*bspF::bspF* bacteria similarly converted their original LAMP1‐positive eBCV into LAMP1‐negative rBCVs (Fig 1A), indicating that BspF does not overtly contribute to rBCV biogenesis. We then analyzed the replication efficiency of the ∆*bspF* mutant in BMMs by single‐cell analysis of intracellular bacterial numbers. Compared with wildtype bacteria that showed a broad range of intracellular replication levels and the non‐replicating ∆*virB11* mutant, ∆*bspF* bacteria displayed significantly reduced replication (Fig 1B; *P* = 0.022), which was complemented genetically (Fig 1B). Hence, BspF contributes to optimal intracellular growth of *Brucella* within rBCVs, but not rBCV biogenesis. Additionally, the replication defect of ∆*bspF* bacteria was rescued in BMMs expressing GFP‐BspF, but not GFP (Fig 1C), indicating that ectopic expression of BspF in mammalian cells functionally mimics bacterially delivered BspF.

### BspF interferes with post‐Golgi secretory traffic

**Figure 2:**
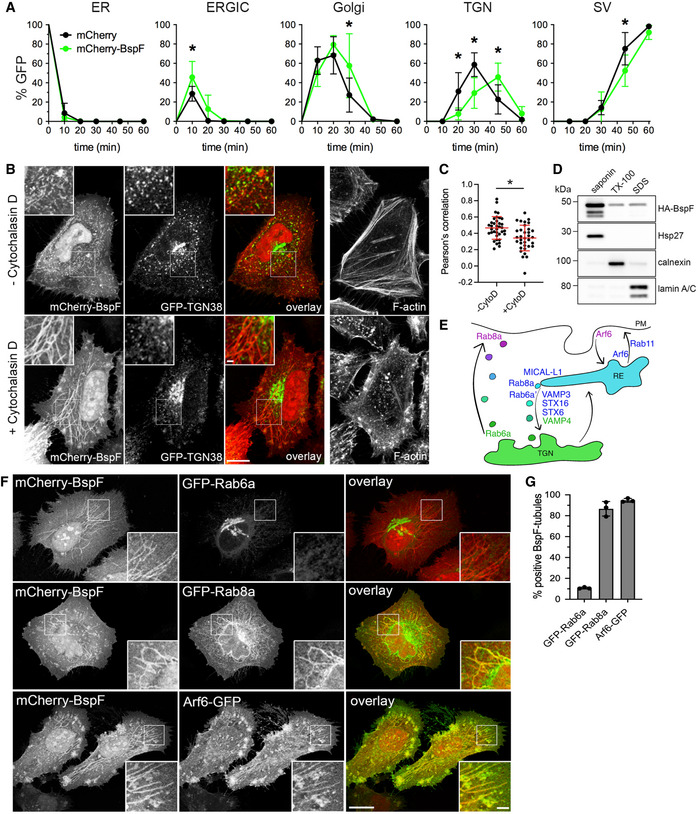
BspF targets the tubular recycling endosome‐to‐TGN transport pathway Quantification of ss‐eGFP‐FKBP^F36 M^ trafficking in HeLa(M)‐C1 cells transfected for 24 h with pmCherry (mCherry) or pmCherry‐BspF (mCherry‐BspF). Rapamycin was added to initiate secretory traffic of ss‐eGFP‐FKBP^F36 M^ and its colocalization with Calnexin (ER), ERGIC‐53 (ERGIC), GM130 (Golgi), p230 (TGN), or secretory vesicles (SV) were scored over a 60‐min time course. Data are means ± SD from *n* = 3 independent experiments. Asterisks indicate statistically significant differences between mCherry‐ and mCherry‐BspF‐expressing cells as determined by a two‐way ANOVA with Sidak’s multiple comparisons test (*P* < 0.05).Representative confocal fluorescence micrographs of HeLa cells co‐transfected for 24 h to produce GFP‐TGN38 and mCherry‐BspF and stained for F‐actin with AlexaFluor™647‐phalloidin. Cells were left untreated or treated with Cytochalasin D (200 nM) for 30 min prior to fixation. Scale bars: 10 and 1 µm (insets).Quantification of colocalization between mCherry‐BspF and GFP‐TGN38 in untreated (−CytoD) and Cytochalasin D‐treated (+CytoD) HeLa cells. Regions of interests (ROI, representative shown as insets in panel B) were randomly selected, and a Pearson’s correlation coefficient was calculated using NIH Fiji image analysis software and Coloc_2 plug‐in. Data are means ± SD from *n* = 3 independent experiments in which 2 ROIs from 10 cells (*n* = 20) were analyzed per experiment. The asterisk indicates a statistically significant difference between treatments as determined by a Mann–Whitney test (*P* < 0.05).Representative Western blot analysis of HeLa cells transfected for 24 h to produce HA‐BspF, separated into saponin‐, Triton X‐100–, and SDS‐soluble fractions and probed for HA‐BspF, Hsp27 (cytosol), Calnexin (membranes), and Lamin A/C (nucleus).Schematic depicting key host proteins that control transport pathways associated with the TGN‐RE‐plasma membrane compartment. Protein colors depict their compartmentalized functions.Representative confocal fluorescence micrographs of HeLa cells co‐transfected for 24 h to produce mCherry‐BspF and either GFP‐Rab6a, GFP‐Rab8a, or Arf6‐GFP and treated with Cytochalasin D (200 nM) for 30 min prior to fixation. Scale bars: 10 and 2 µm (insets).Quantification of localization of GFP‐Rab6a, GFP‐Rab8a, and Arf6‐GFP on mCherry‐BspF‐labeled tubules in transfected HeLa cells. Data are means ± SD from *n* = 3 independent experiments, in which at least 300 individual cells per experiment were analyzed. Source data are available online for this figure.

**Figure EV1:**
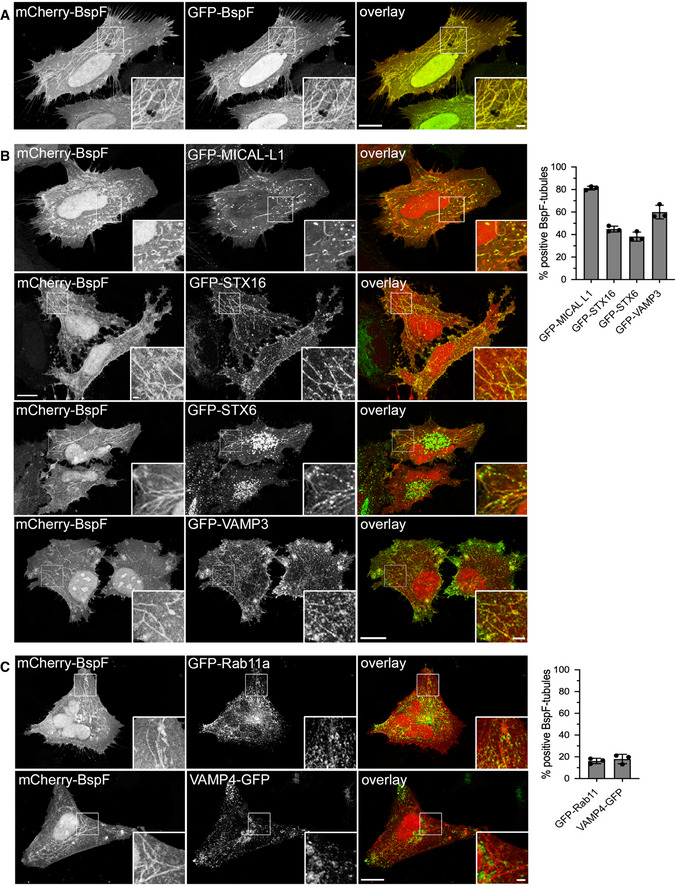
BspF localizes to the endosomal recycling compartment Representative confocal fluorescence micrograph of HeLa cells co‐transfected for 24 h to produce mCherry‐BspF and GFP‐BspF and treated with Cytochalasin D (200 nM) for 30 min prior to fixation. Scale bars: 10 and 2 µm (insets).Representative confocal fluorescence micrographs of HeLa cells co‐transfected for 24 h to produce mCherry‐BspF and either GFP‐MICAL‐L1, GFP‐STX16, GFP‐STX6, or GFP‐VAMP3 and treated with Cytochalasin D (200 nM) for 30 min prior to fixation. Scale bars: 10 and 2 µm (insets). Localization of GFP‐MICAL‐L1, GFP‐STX16, GFP‐STX6, or GFP‐VAMP3 to mCherry‐BspF‐labeled tubules was quantified in at least 300 individual cells per experiment. Data are means ± SD from *n* = 3 independent experiments.Representative confocal fluorescence micrographs of HeLa cells co‐transfected for 24 h to produce mCherry‐BspF and either GFP‐Rab11a or VAMP4‐GFP and treated with Cytochalasin D (200 nM) for 30 min prior to fixation. Scale bars: 10 and 2 µm (insets). Localization of GFP‐Rab11a or VAMP4‐GFP to mCherry‐BspF‐labeled tubules was quantified in at least 300 individual cells per experiment. Data are means ± SD from *n* = 3 independent experiments.

Our previous characterization of BspF as interfering with host secretion (Myeni *et al*, 2013) suggested that this effector targets membrane vesicular transport. We therefore analyzed the effect of ectopically expressed mCherry‐tagged BspF on the traffic of the secretory reporter ss‐eGFP‐FKBP^F36M^ in HeLa(M)‐C1 cells (Gordon *et al*, 2010; Miller *et al*, 2017). Compared with cells expressing mCherry, mCherry‐BspF expression caused a significant delay in cargo traffic within the Golgi apparatus and TGN (Fig 2A), suggesting that BspF targets a post‐Golgi secretory transport event. Consistently, mCherry‐BspF localized to vesiculotubular structures that were partially labeled with the TGN‐to‐plasma membrane cargo receptor TGN38 (Fig 2B and C). Mild treatment with Cytochalasin D, which promotes tubulation of this compartment (Hattula *et al*, 2006), showed a dramatic, enhanced localization of mCherry‐BspF to tubular structures that still partially colocalized with TGN38‐positive vesicles (Fig 2B and C). GFP‐BspF also localized to the same tubular structures as mCherry‐BspF (Fig EV1A). Detergent‐based fractionation of HeLa cells showed that HA‐BspF partitioned between the saponin‐soluble cytosolic and Triton X‐100–soluble membrane fractions (Fig 2D), consistent with a partial association with tubular membranes. Hence, ectopically expressed BspF targets a tubular membrane compartment likely involved in transport between the Golgi apparatus and plasma membrane.

### BspF targets the tubular recycling endosome‐to‐TGN transport pathway

To further identify the BspF‐targeted compartment, we tested by fluorescence microscopy an array of GFP‐tagged Arf‐ and Rab‐family GTPases known to regulate various steps in TGN‐plasma membrane transport for their localization with BspF (Fig 2E). We first examined recruitment of GFP‐Rab6a and GFP‐Rab8a, based on the multiple roles of these GTPases in TGN‐to‐plasma membrane transport (Huber *et al*, 1993; Ang *et al*, 2003; Grigoriev *et al*, 2007, 2011; Micaroni *et al*, 2013). In Cytochalasin D‐treated HeLa cells, mCherry‐BspF‐positive tubules accumulated Rab8a, which localizes to the recycling endosome (RE), but not Rab6a, which localizes to the TGN (Fig 2F and G), suggesting that BspF‐labeled tubules are of endosomal nature. Consistently, the endosomal GTPase Arf6 also accumulated on these tubules (Fig 2F and G). Rab8a regulates exocytic transport between the TGN and plasma membrane via the RE (Ang *et al*, 2003, 2004; Henry & Sheff, 2008; Lucken‐Ardjomande Häsler *et al*, 2020) and contributes to membrane recycling regulated by Arf6 and Rab11 (Hattula *et al*, 2006; Knödler *et al*, 2010; Chen *et al*, 2017). Arf6 also regulates Rab8a‐dependent transport between the RE and the TGN by recruiting the endocytic adaptor MICAL‐L1 (Hattula *et al*, 2006; Rahajeng *et al*, 2012). These transport events also involve the TGN‐associated soluble N‐ethylmaleimide‐sensitive factor attachment protein receptors (SNAREs) Syntaxin16 (STX16) and Syntaxin 6 (STX6), the early endosome‐associated SNARE VAMP4, and the recycling endosome‐associated SNARE VAMP3 (Fig 2E) (Mallard *et al*, 2002; Lucken‐Ardjomande Häsler *et al*, 2020). MICAL‐L1, STX6, STX16, and VAMP3 localized to mCherry‐BspF‐decorated tubules (Fig EV1B), unlike VAMP4 (Fig EV1C), identifying additional TGN‐RE trafficking components on these membrane structures. The recycling endosomal GTPase Rab11a only labeled structures adjacent to BspF‐positive tubules (Fig EV1C), indicating that this tubular compartment is distinct from the RE‐to‐plasma membrane recycling pathway. Collectively, these findings indicate that BspF intrinsically targets a tubular compartment between the TGN and the RE that may be regulated by Arf6 and Rab8a.

### BspF interferes with Arf6‐, Rab8a‐, and Rab6a/a′‐dependent retrograde transport during infection

**Figure EV2:**
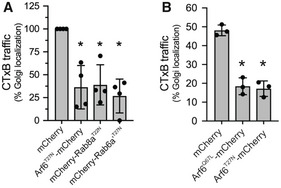
Retrograde transport of Cholera toxin depends upon Arf6, Rab8a, and Rab6a′ in HeLa cells and BMMs A, BQuantification of CTxB transport to the Golgi apparatus in either HeLa cells producing either mCherry, Arf6^T27N^‐mCherry, mCherry‐Rab8a^T22N^, or mCherry‐Rab6a′^T27N^ (A), or in BMMs producing either mCherry, Arf6^Q67L^‐mCherry, or Arf6^T27N^‐mCherry (B). Cells were transfected for 24 h (A) or transduced for 48 h (B) then incubated on ice with AlexaFluor488™‐Cholera Toxin subunit B (CTxB) for binding followed by a 20‐min (A) or 30‐min (B) incubation at 37°C to allow for CTxB retrograde transport to the Golgi apparatus (stained using an anti‐GM130 antibody). CTxB retrograde transport is expressed as percentages of cells in which CTxB colocalized with the GM130 Golgi marker. Data are means ± SD from *n* = 3 to 4 independent experiments, in which 100 cells were analyzed per experiment. Asterisks indicate statistically significant differences compared with mCherry‐producing cells as determined by a one‐way ANOVA with Dunnett’s multiple comparisons test (*P* < 0.05).

**Figure 3:**
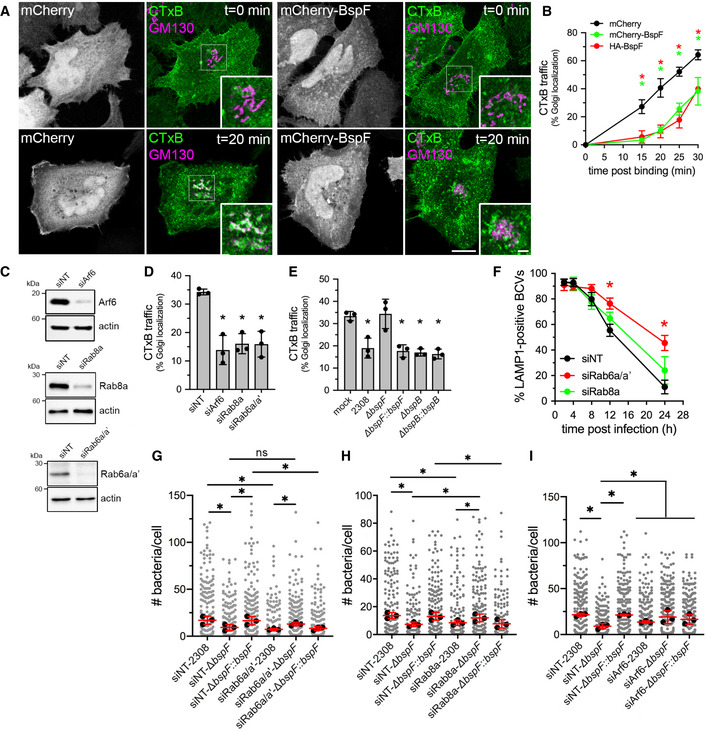
BspF modulates an Arf6/Rab8a‐dependent TGN‐RE transport pathway that is required for *Brucella* replication ARepresentative confocal fluorescence micrographs of HeLa cells transfected for 24 h to produce either mCherry or mCherry‐BspF (grayscale panels), incubated on ice with AlexaFluor™488‐Cholera Toxin subunit B (CTxB; green) and shifted to 37°C for 20 min to allow for CTxB retrograde transport to the Golgi apparatus (stained using an anti‐GM130 antibody; purple). CTxB accumulation within Golgi structures appears white in overlays. Scale bars: 10 and 2 µm (insets).BQuantification of CTxB transport to the Golgi apparatus in HeLa cells producing either mCherry, mCherry‐BspF, or HA‐BspF over a 30‐min time course, expressed as percentages of cells in which CTxB colocalized with the GM130 Golgi marker, as in (A). Data are means ± SD from *n* = 3 independent experiments, in which 100 cells were analyzed per experiment. Asterisks indicate statistically significant differences compared with mCherry‐producing cells as determined by a two‐way ANOVA with Tukey’s multiple comparisons test (*P* < 0.05).CRepresentative Western blot analysis of Arf6, Rab8a, and Rab6a/a′ depletions in BMMs following siRNA‐mediated knockdowns, compared with non‐targeting siRNA (siNT) treatments. β‐actin was used as loading control.DQuantification of CTxB transport to the Golgi apparatus in BMMs following siRNA‐mediated depletion of either Arf6 (siArf6), Rab8a (siRab8a), or Rab6a/a′ (siRab6a/a′) after AlexaFluor™488‐CTxB binding on ice followed by 30‐min incubation at 37°C. Data are means ± SD from *n* = 3 independent experiments, in which 100 cells were analyzed per experiment. Asterisks indicate a statistically significant difference compared with siNT control cells as determined by a one‐way ANOVA with Tukey’s multiple comparisons test (*P* < 0.05).EQuantification of CTxB transport to the Golgi apparatus in BMMs that were either mock‐infected or infected with wild‐type (2308), Δ*bspF*, complemented ∆*bspF* (Δ*bspF::bspF*), ∆*bspB* or complemented ∆*bspB* (Δ*bspB::bspB*) bacteria for 24 h, incubated for 30 min with AlexaFluor™488‐CTxB on ice for binding followed by 30‐min incubation at 37°C. Data are means ± SD from *n* = 3 independent experiments, in which 100 cells were analyzed per experiment. Asterisks indicate a statistically significant difference compared with mock‐infected cells as determined by a one‐way ANOVA with Tukey’s multiple comparisons test (*P* < 0.05).FrBCV biogenesis in BMMs treated with either non‐targeting siNT, siRab6a/a′, or siRab8a siRNAs and infected with wild‐type (2308) bacteria. Data are means ± SD of *n* = 3 independent experiments, in which 100 BCVs were analyzed per experiment. Asterisks indicate statistically significant differences (*P* < 0.05, two‐way ANOVA followed by Dunnett’s multiple comparisons test) compared with control (2308).G–I*Brucella* replication in BMMs treated with non‐targeting siRNAs (siNT), or siRNAs against Rab6a/a′ (siRab6a/a′) (G), Rab8a (siRab8a) (H), or Arf6 (siArf6) (I) and infected with either wild‐type (2308), Δ*bspF*, or complemented ∆*bspF* (Δ*bspF::bspF*) bacteria, measured as number of bacteria per cell at 24 h pi. Data are means ± SD of *n* = 3 independent experiments in which at least 100 cells were analyzed per experiment. Gray dots represent individual cells analyzed; black dots indicate means of individual experiments. Asterisks indicate statistically significant differences (*P* < 0.05, one‐way ANOVA followed by Dunnett’s multiple comparisons test) between test and control conditions. Source data are available online for this figure.

Since BspF targets the TGN‐RE‐plasma membrane network, we tested whether its expression alters transport through this compartment by monitoring traffic of the Cholera Toxin subunit B (CTxB) from the plasma membrane to the Golgi apparatus via the RE (Lencer, 2003). CTxB transport to the Golgi apparatus in HeLa cells was dependent upon Arf6, Rab8a (Hattula *et al*, 2006) and the Rab6a′ isoform (Mallard *et al*, 2002), as overexpression of either dominant negative alleles Arf6^T27N^‐mCherry, mCherry‐Rab8a^T22N^, or mCherry‐Rab6a′^T27N^ inhibited CTxB traffic to the Golgi complex (Fig EV2A). Compared with control cells expressing mCherry alone, where surface‐bound CTxB had reached the Golgi compartment after 20 min (Fig 3A and B), HeLa cells expressing mCherry‐BspF or hemagglutinin (HA)‐tagged BspF showed a significant delay in CTxB traffic to the Golgi complex (Fig 3A and B), demonstrating that BspF interferes with retrograde transport through the RE. To extend these observations to the context of *Brucella* infections, we first verified that CTxB traffic to the Golgi apparatus in BMMs is dependent upon Arf6, Rab8a, and the Rab6a/a′ isoforms in BMMs. Their individual depletions via siRNA nucleofection (84 ± 13% depletion for Arf6; 85 ± 9.0% depletion for Rab8a; 89 ± 6.9% depletion for Rab6a/a′) impaired CTxB traffic (Fig 3C and D). BMMs were then infected for 24 h with various *B. abortus* strains and CTxB traffic analyzed in infected cells. Compared with mock‐infected cells, infection with wild‐type (2308) bacteria inhibited CTxB traffic to the Golgi apparatus (Fig 3E), indicating that *Brucella* interferes with trafficking through the RE during infection. Unlike the wild‐type strain, ∆*bspF* bacteria did not inhibit CTxB transport to the Golgi, a phenotypic defect that was complemented genetically (Fig 3E). Hence, BspF is required for *Brucella* inhibition of retrograde transport to the TGN during infection. To test the specificity of BspF’s effect, we examined the behavior of an in‐frame deletion mutant in *bspB*, which encodes a Golgi‐targeting effector required for bacterial replication that impairs COG‐dependent secretory traffic (Myeni *et al*, 2013; Miller *et al*, 2017). Unlike the ∆*bspF* mutant, the replication‐impaired ∆*bspB* mutant (Miller *et al*, 2017) and its genetic complement inhibited CTxB transport to the same extent as wild‐type bacteria (Fig 3E), emphasizing the specificity of BspF’s effect on retrograde transport. Collectively, these findings demonstrate that *B. abortus* modulates an Arf6‐, Rab8a‐, and Rab6a′‐dependent membrane transport pathway between the RE and the TGN via delivery of BspF.

### Rab6a‐ and Rab8a‐dependent transport pathways differentially contribute to *Brucella* replication

The targeting of the RE‐to‐TGN transport pathway by the replication‐promoting effector BspF supports the possibility that *Brucella* modulates retrograde transport for the purpose of intracellular proliferation. To test this hypothesis, we first depleted in BMMs the closely related Rab6a and Rab6a′ isoforms via siRNA nucleofection, as these isoforms regulate anterograde and retrograde vesicular transport to and from the TGN, and from the TGN to the ER (Mallard *et al*, 2002; Del Nery *et al*, 2006; Utskarpen *et al*, 2006). Rab6a/a′ depletion significantly affected the kinetics of rBCV biogenesis in BMMs (Fig 3F) and impaired replication of wild‐type *B. abortus* at 24 h pi (Fig 3F and G; 87 ± 9.4% and 94 ± 8.3% depletion, respectively), indicating that Rab6a/a′ isoforms contribute to *Brucella*’s intracellular cycle. Because of their high sequence identity, we could not individually deplete Rab6a and Rab6a′ and assess their individual contributions to rBCV biogenesis and bacterial replication. Nonetheless, depletion of Rab8a, which controls the same retrograde transport pathway between the RE and the TGN as Rab6a′ (Mallard *et al*, 2002; Hattula *et al*, 2006; Roland *et al*, 2007) (Fig 2E), significantly affected bacterial replication (Fig 3H; 83 ± 6.6% depletion) but not rBCV biogenesis (Fig 3F; 93 ± 7.1% depletion). These results suggest that Rab6a‐dependent transport contributes to rBCV biogenesis and consequently bacterial replication, while Rab6a′/Rab8a‐dependent RE‐to‐TGN transport only contributes to *Brucella* replication in rBCVs.

### Inhibition of Rab8a‐ and Arf6‐dependent transport suppresses BspF deficiency in *Brucella* replication

Based on the effect of Rab6a/a′ and Rab8a depletions on *Brucella* replication, we next examined their effects on the replication‐impaired ∆*bspF* mutant. Interestingly, depletion of Rab8a, but not Rab6a/a′, suppressed the replication defect of ∆*bspF* bacteria, while the complemented ∆*bspF* mutant behaved like wild‐type bacteria (Fig 3G and H). This indicates that inhibition of Rab8a‐dependent transport functionally mimics BspF’s function in bacterial replication, suggesting that BspF interferes with a vesicular transport process regulated by Rab8a. Given the functional connection between Arf6 and Rab8a regulatory function in the RE (Hattula *et al*, 2006; Rahajeng *et al*, 2012), we next tested the effect of Arf6 depletion on the replication of wild‐type and BspF‐deficient *B. abortus* strains. While Arf6 depletion (82 ± 11%) did not significantly impair replication of wild‐type bacteria, it restored replication of ∆*bspF* bacteria (Fig 3I). These findings indicate that BspF’s role in bacterial replication is via the Arf6/Rab8a regulatory cascade in the RE.

### BspF interacts and interferes with the GTPase‐activating protein ACAP1

**Figure 4:**
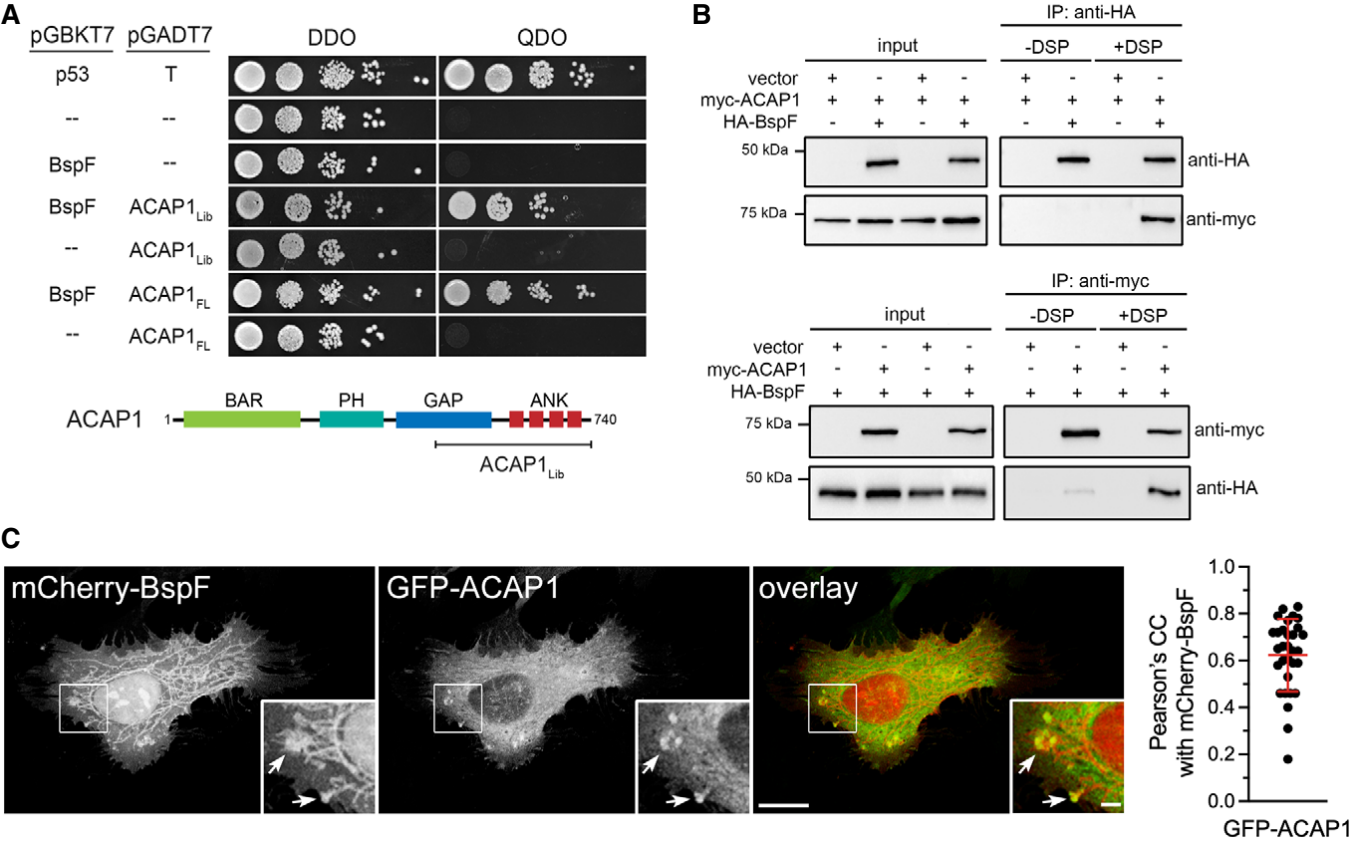
BspF interacts with the Arf6 GTPase‐activating protein ACAP1 Yeast two‐hybrid mating screen showing interaction of BspF with a fragment of ACAP1 (amino acid residues 460–740; ACAP1_Lib_) or full‐length ACAP1 (ACAP1_FL_), compared with positive (p53/T antigen) and negative (empty vectors) control matings plated on permissive double dropout (DDO) or selective quadruple dropout (QDO) media. ACAP1 schematic indicates the region of interaction initially identified (ACAP1_Lib_).Representative co‐immunoprecipitations of HA‐BspF and myc‐ACAP1 in HeLa cells. HeLa cells were transfected to either co‐produce or individually produce HA‐BspF and myc‐ACAP1 and either HA‐BspF or myc‐ACAP1 were immunoprecipitated using either anti‐HA‐conjugated (upper panel) or anti‐myc‐conjugated (lower panel) magnetic beads following cross‐linking (+DSP) or not (‐DSP) with dithiobis[succinimidylpropionate]. Input lysates (10% of the post‐nuclear supernatant) and co‐immunoprecipitates were separated by SDS–PAGE and probed for HA‐BspF and myc‐ACAP1 by Western blotting.Representative confocal micrographs of HeLa cells transfected to produce mCherry‐BspF and GFP‐ACAP1 and treated with Cytochalasin D (200 nM) for 30 min and quantification of colocalization between mCherry‐BspF and GFP‐ACAP1. Arrows indicate areas of BspF and ACAP1 colocalization. Scale bars: 10 µm and 2 µm (insets). Data are means ± SD from *n* = 3 independent experiments in which 10 cells were analyzed per experiment. Pearson’s correlation coefficients were calculated from whole cells using NIH Fiji image analysis software and Coloc_2 plug‐in. Source data are available online for this figure.

**Figure 5:**
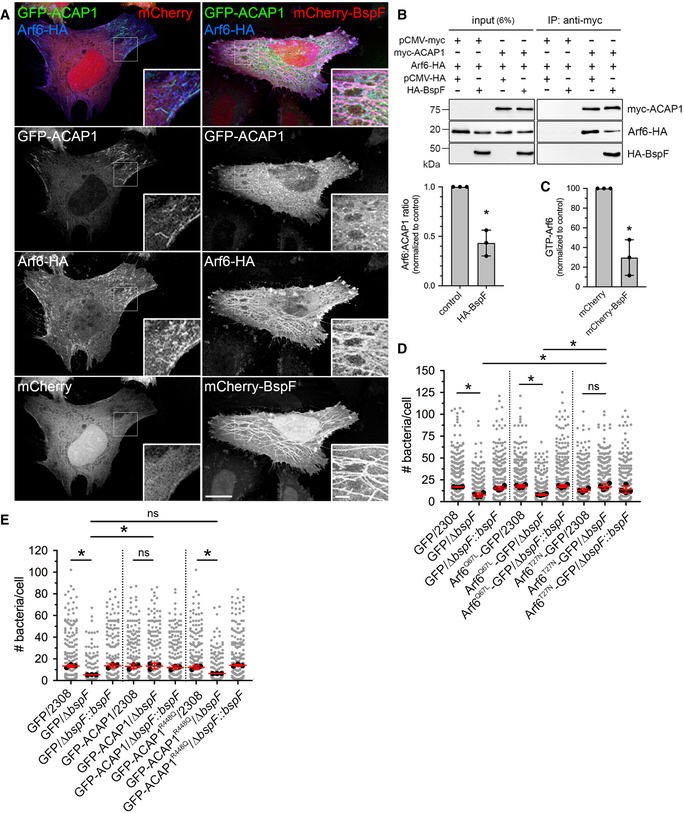
BspF interferes with ACAP1 to modulate Arf6 activity Representative confocal micrograph of HeLa cells transfected to produce either mCherry (red), GFP‐ACAP1 (green), and Arf6‐HA (blue; left hand panels) or mCherry‐BspF (red), GFP‐ACAP1 (green), and HA‐Arf6 (blue; right hand panels) and treated with Cytochalasin D (200 nM) for 30 min prior to fixation. Scale bars: 10 µm and 2 µm (insets).Representative Western blot analysis of co‐immunoprecipitations of myc‐ACAP1 and Arf6‐HA in the presence or absence of HA‐BspF. HeLa cells were transfected to produce Arf6‐HA and combinations of myc‐ACAP1 and HA‐BspF, or not, and myc‐ACAP1 was immunoprecipitated using anti‐myc‐conjugated magnetic beads. Input lysates (6% of post‐nuclear supernatants) and co‐immunoprecipitates were separated by SDS–PAGE and probed for Arf6‐HA, HA‐BspF and myc‐ACAP1 by Western blotting. Quantification of the Arf6/ACAP1 ratio was performed by densitometric analysis. Data are means ± SD of 3 independent experiments. The asterisk indicates a statistically significant difference (*P* = 0.0017, unpaired Student’s *t*‐test) between BspF‐producing and control conditions.Quantification of Arf6 activity (GTP‐Arf6) in HeLa cells transfected to produce either mCherry and Arf6‐HA or mCherry‐BspF and Arf6‐HA by G‐LISA. Data are means ± SD of *n* = 3 independent experiments, normalized to mCherry‐producing controls. The asterisk indicates a statistically significant difference (*P* = 0.0026, unpaired Student’s *t*‐test) between BspF‐producing and control conditions.Bacterial replication in BMMs transduced to either produce GFP, Arf6^Q67L^‐GFP, or Arf6^T27N^‐GFP and infected with either wild‐type (2308), Δ*bspF*, or complemented ∆*bspF* (Δ*bspF::bspF*) bacteria for 24 h. Data are means ± SD of *n* = 4 independent experiments, in which at least 100 cells were analyzed per experiment. Gray dots represent individual cells analyzed (*n* > 300); black dots indicate means of individual experiments. Asterisks indicate statistically significant differences (*P* < 0.05, two‐way ANOVA followed by Dunnett’s multiple comparisons test) between test and control conditions.Bacterial replication in BMMs transduced to either produce GFP, GFP‐ACAP1, or GFP‐ACAP1^R448Q^ and infected with either wild‐type (2308), Δ*bspF*, or complemented ∆*bspF* (Δ*bspF::bspF*) bacteria for 24 h. Data are means ± SD of *n* = 3 independent experiments, in which at least 100 cells were analyzed per experiment. Gray dots represent individual cells analyzed (*n* > 300); black dots indicate means of individual experiments. Asterisks indicate statistically significant differences (*P* < 0.05, two‐way ANOVA followed by Dunnett’s multiple comparisons test) between test and control conditions. Source data are available online for this figure.

**Figure EV3:**
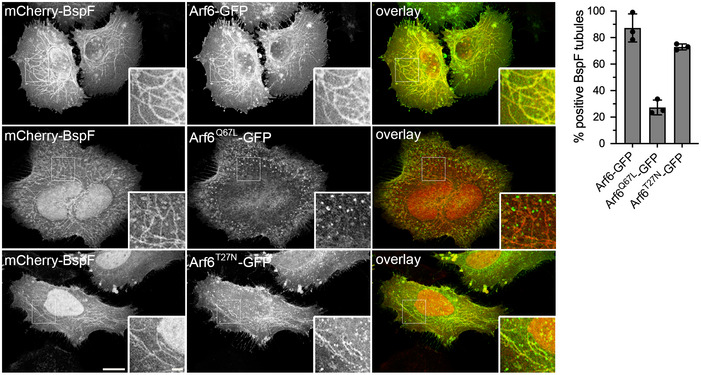
Localization of Arf6‐GFP alleles to mCherry‐BspF‐labeled tubules Representative confocal fluorescence micrographs of HeLa cells co‐transfected for 24 h to produce mCherry‐BspF and either Arf6‐GFP, Arf6^Q67L^‐GFP, or Arf6^T27N^‐GFP and treated with Cytochalasin D (200 nM) for 30 min prior to fixation. Scale bars: 10 and 2 µm (insets). Localization of Arf6‐GFP, Arf6^Q67L^‐GFP, or Arf6^T27N^‐GFP to mCherry‐BspF‐labeled tubules was quantified in at least 300 individual cells per experiment. Data are means ± SD from *n* = 3 independent experiments.

To gain insight into the mode of action of BspF on RE‐TGN transport, we performed a Yeast two‐hybrid screen and found that BspF interacted with a clone expressing a C‐terminal fragment (residues 460–740) of the GTPase‐activating protein (GAP) ACAP1, which was confirmed using a clone expressing full‐length ACAP1 (Fig 4A). The BspF–ACAP1 interaction was corroborated in mammalian cells but appeared weak, as HA‐tagged BspF and myc‐ACAP1 reciprocally co‐immunoprecipitated only upon cross‐linking when co‐expressed in HeLa cells (Fig 4B). ACAP1 is a GAP that regulates Arf6 activity in endocytic recycling from the RE (Jackson *et al*, 2000; Hattula *et al*, 2006; Rahajeng *et al*, 2012; Chen *et al*, 2014). Confocal fluorescence microscopy analysis of GFP‐ACAP1 and mCherry‐BspF in Cytochalasin D‐treated HeLa cells showed a significant colocalization of ACAP1 and BspF (Pearson’s correlation coefficient of 0.62), mostly in coalesced endosomes at the cell periphery but not on BspF‐labeled tubules emanating from these structures (Fig 4C), which nonetheless accumulated Arf6 (Fig 2F). While ACAP1 and Arf6 colocalized on tubular endosomes in mCherry‐producing, Cytochalasin D‐treated HeLa cells (Fig 5A), mCherry‐BspF production abrogated ACAP1 accumulation on tubular recycling endosomes (Fig 5A), suggesting it interferes with ACAP1–Arf6 interactions. To test this hypothesis, we quantified the effect of BspF expression on the ACAP1–Arf6 interaction via co‐immunoprecipitation, by co‐expressing myc‐ACAP1 and Arf6‐HA in the presence or absence of HA‐BspF in HeLa cells. Immunoprecipitation of myc‐ACAP1 showed that expression of BspF caused a 57 ± 13% decrease in Arf6 co‐immunoprecipitation (Fig 5B), indicating that BspF affects the ACAP1–Arf6 interaction. Decreased interaction of a GTPase with its GAP may result from either increased GAP activity, enhancing dissociation following GTPase inactivation, or may alternatively indicate interference with GAP function resulting in sustained GTPase activation. To discriminate between these possibilities, we measured Arf6 activation in cells expressing BspF or not. Production of mCherry‐BspF in HeLa cells decreased the levels of active, GTP‐bound Arf6 to 29.7 ± 18.1% of those in mCherry‐producing, control cells (Fig 5C), indicating that BspF causes Arf6 inactivation. Consistently, BspF‐labeled tubules were predominantly labeled with the dominant inactive allele Arf6^T27N^ and not the dominant active allele Arf6^Q67L^ (Fig EV3), in agreement with the known association of inactive Arf6 with recycling endosomal membranes (Hattula *et al*, 2006). To confirm the effect of BspF on Arf6 activity, we next examined whether Arf6 dominant alleles production in BMMs affects BspF‐dependent bacterial replication. Compared with GFP or Arf6^Q67L^‐GFP production, expression of Arf6^T27N^‐GFP rescued the replication defect of ∆*bspF* bacteria without affecting that of wild‐type or complemented ∆*bspF::bspF* bacteria (Fig 5D), mirroring the effect of Arf6 depletion (Fig 3I). Hence, inactivation of Arf6 mimics the role of bacterially delivered BspF in bacterial replication. Interestingly, Arf6^Q67L^‐GFP and Arf6^T27N^‐GFP production in BMMs equally interfered with CTxB traffic (Fig EV2B), indicating that rescuing of the ∆*bspF* mutant replication defect is not simply via inhibition of retrograde transport but also requires inactive Arf6. Considering BspF interaction with ACAP1 (Fig 4) and ACAP1’s role as an Arf6 GAP (Jackson *et al*, 2000), if BspF enhances ACAP1 function to downmodulate Arf6 activity, we reasoned that overexpression of ACAP1 in BMMs should also suppress the bacterial replication defects caused by *bspF* deletion. Retroviral expression of GFP‐ACAP1 in BMMs specifically restored replication of the ∆*bspF* mutant to wild‐type levels (Fig 5E), indicating that enhancing ACAP1 activity mimics BspF’s function. By contrast, expression of the catalytically inactive GAP mutant allele ACAP1^R448Q^ (Jackson *et al*, 2000) failed to rescue the replication defect of ∆*bspF* bacteria (Fig 5E), demonstrating that ACAP1’s rescuing effect is via its GAP activity. Taken together, these results argue that BspF enhances ACAP1 GAP‐dependent function to downmodulate Arf6 activity in order to promote bacterial replication.

### BspF promotes recruitment of TGN‐associated membranes to rBCVs

**Figure 6:**
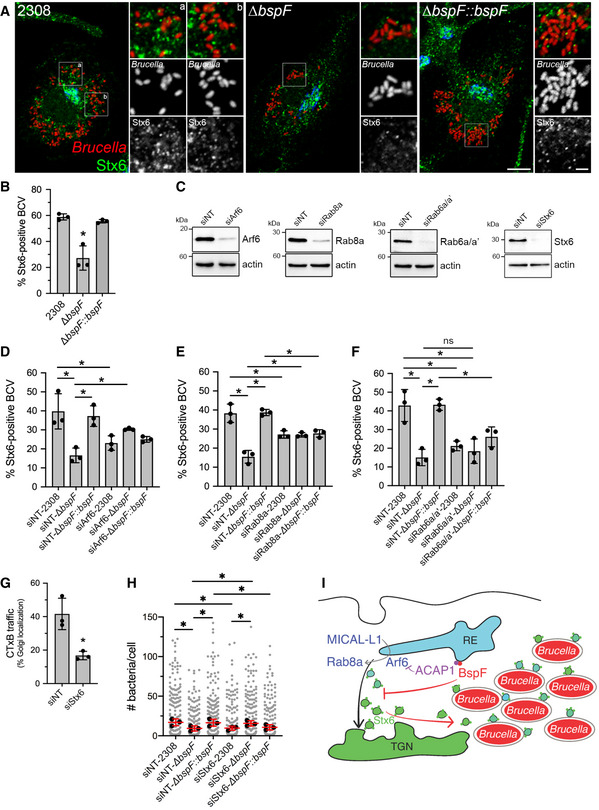
BspF promotes recruitment of TGN‐derived membranes to rBCVs in an Arf6‐/Rab8a‐dependent manner ARepresentative confocal micrographs of BMMs infected with either wild‐type (2308), ∆*bspF*, or complemented ∆*bspF* (∆*bspF::bspF*) bacteria (red) for 24 h and immunostained for the TGN vesicular marker Stx6 (green) and GM130 (blue). Scale bars, 10 µm and 2 µm (insets). Magnified insets show the association between Stx6‐positive vesicles and rBCVs.BRecruitment of Stx6‐positive vesicles to rBCVs (expressed as percentage of Stx6‐positive BCVs) in BMMs infected for 24 h with either wild‐type (2308), ∆*bspF*, or complemented ∆*bspF* (∆*bspF*::*bspF*) bacteria. Data are means ± SD from *n* = 3 independent experiments, in which at least 300 BCVs were analyzed per experiment via CellProfiler image analysis. Asterisks indicate statistically significant differences compared with 2308‐infected BMMs as determined by one‐way ANOVA with Tukey’s multiple comparisons test (*P* < 0.05).CRepresentative Western blot analysis of Arf6, Rab8a, Rab6a/a′, and Stx6 depletions in BMMs following siRNA‐mediated knockdowns, compared with non‐targeting siRNA (siNT) treatments. β‐actin was used as loading control.D–FRecruitment of Stx6‐positive vesicles to rBCVs in BMMs treated with non‐targeting siRNAs (siNT), siRNAs against Arf6 (siArf6) (D), Rab8a (siRab8a) (E), or Rab6a/a′ (siRab6a/a′) (F) and infected for 24 h with either wild‐type (2308), Δ*bspF*, or complemented ∆*bspF* (Δ*bspF::bspF*) bacteria. Data are means ± SD of *n* = 3 independent experiments, in which 200 BCVs were analyzed per experiment. Asterisks indicate statistically significant differences (*P* < 0.05, one‐way ANOVA followed by Dunnett’s multiple comparisons test) between test and control conditions; ns, not significant.GQuantification of CTxB retrograde transport in BMMs following siRNA‐mediated depletion of Stx6 (siStx6) after AlexaFluor™488‐CTxB binding on ice followed by 30‐min incubation at 37°C. Data are means ± SD from *n* = 3 independent experiments, in which 100 cells were analyzed per experiment. The asterisk indicates a statistically significant difference (*P* = 0.0114, unpaired Student’s *t*‐test) compared with the siNT control.H*Brucella* replication in BMMs treated with either non‐targeting siRNAs (siNT), or siRNAs against Stx6 (siStx6) and infected for 24 h with either wild‐type (2308), Δ*bspF*, or complemented ∆*bspF* (Δ*bspF::bspF*) bacteria. Data are means ± SD of *n* = 3 independent experiments, in which at least 100 cells were analyzed per experiment. Gray dots represent individual cells analyzed (*n* > 300); black dots indicate means of individual experiments. Asterisks indicate statistically significant differences (*P* < 0.05, one‐way ANOVA followed by Dunnett’s multiple comparisons test) between test and control conditions.IModel of BspF remodeling of TGN‐RE membrane traffic. Bacterially delivered BspF targets RE membranes where it binds ACAP1 and promotes inactivation of Arf6. Increased turnover of active Arf6 results in inhibition of the Arf6/Rab8a cascade and retrograde RE‐TGN transport, which alters TGN‐derived vesicular traffic and redirects Stx6‐positive vesicles to rBCVs in a process that promotes intravacuolar bacterial growth. Source data are available online for this figure.

**Figure EV4:**
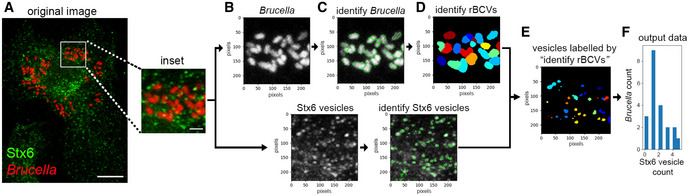
CellProfiler analysis pipeline of Stx6‐positive vesicle recruitment to rBCVs A confocal micrograph inset (10.5 µm^2^ area) from a BMM infected with wild‐type DsRed_m_‐expressing *B. abortus* and stained for endogenous Syntaxin 6 (AlexaFluor™488‐Stx6) was selected in the DsRed channel (*Brucella*) and input into CellProfiler for analysis. Scale bars, 10 and 2 µm.*Color to gray* module split the red (*Brucella*) and green (Stx6) channels and reverted the images to gray scale.*Identify primary objects* module identified individual *Brucella* and Stx6‐positive vesicles based on their size.*Expand or shrink objects* module expanded the size of individual *Brucella* by 6 pixels to encompass whole rBCVs and associated vesicles.*Relate objects* module identified Stx6‐positive vesicles within the rBCV area and filtered out non‐associated vesicles.*Classify objects* module counted the number of vesicles associated with each rBCV (expanded *Brucella*). X‐ and y‐axes represent pixels coordinates. The output data counted the number of vesicles associated with each *Brucella*, which was derived to determine the percentage of Stx6‐positive rBCVs.

BspF‐dependent interference with TGN‐RE transport and the roles of TGN‐associated transport pathways in bacterial replication suggest that *Brucella* remodels post‐Golgi compartments for replication purposes. To test this hypothesis, we examined via confocal fluorescence microscopy whether TGN‐associated membrane carriers are recruited to rBCVs during bacterial replication. Using Stx6 as a generic marker of TGN‐associated vesicles, we found that 58.9 ± 2.4% of wild‐type bacteria‐containing rBCVs were tightly associated with Stx6‐positive vesicles or structures (Figs 6A and B and EV4), indicating interactions of TGN‐associated vesicles with rBCVs. Importantly, ∆*bspF* bacteria were significantly impaired in their ability to recruit Stx6‐positive vesicles to their rBCVs (27.2 ± 9.3% of positive rBCVs), a defect that was genetically complemented (Figs 6A and B and EV4). Hence, recruitment of TGN‐associated vesicles to rBCVs is driven in part by BspF. To determine whether the host transport pathways required for accretion of TGN‐associated vesicles by rBCVs are those targeted by BspF, we next tested the effect of depletions of either Arf6, Rab8a, or Rab6a/a′ on TGN‐associated vesicle recruitment to rBCVs. Depletions of either of these GTPases (82 ± 11% depletion for Arf6; 86 ± 3.5% depletion for Rab8a; 90 ± 4.4% depletion for Rab6a/a′; Fig 6C) decreased Stx6‐positive vesicle recruitment by rBCVs containing wild‐type bacteria (Fig 6D–F), indicating that these events require Arf6/Rab8a‐ and Rab6a/a′‐dependent membrane transport. In agreement with our observations on ∆*bspF* replication (Fig 3G–I), depletions of either Rab8a or Arf6, but not of Rab6a/a′, partially suppressed the defect in Stx6‐positive vesicle recruitment by ∆*bspF* bacteria (Fig 6D–F), further indicating that inactivation of the Arf6/Rab8a regulatory cascade functionally mimics BspF’s effect on TGN‐associated transport. Taken together, these findings show that BspF interferes with Arf6/Rab8a‐regulated membrane transport at the RE to promote recruitment of TGN‐associated vesicles to rBCVs.

### Stx6‐dependent vesicular transport is required for BspF‐dependent *Brucella* optimal replication

To determine whether acquisition of TGN‐associated vesicles by rBCVs contribute to bacterial replication, we targeted Stx6 as a key SNARE protein functioning in TGN‐associated vesicular trafficking, including endosome‐TGN retrograde transport (Laufman *et al*, 2011; Bock *et al*, 2017). Depletion of Stx6 via siRNA nucleofection of BMMs (84 ± 8.0% depletion, Fig 6G) inhibited CTxB traffic to the Golgi apparatus, confirming its role in RE‐TGN retrograde transport in macrophages. Stx6 depletion (87 ± 12% depletion; Fig 6C) significantly impaired replication of wild‐type bacteria (Fig 6H), confirming the importance of TGN‐associated membrane transport for *Brucella* replication and also significantly rescued replication of ∆*bspF* bacteria (Fig 6H). Given the requirement of Stx6 in CTxB transport (Fig 6G), we interpret these results as reflecting the same suppressive effect as that seen through inhibition of Arf6/Rab8a‐dependent transport (Fig 3G–I). Taken together, these findings argue that BspF‐mediated remodeling of Stx6‐dependent, TGN‐associated vesicular traffic promotes *Brucella* replication within rBCVs.

## Discussion

Here, we have uncovered the mode of action of BspF, a *Brucella* T4SS effector that contributes to bacterial growth within the replication‐permissive rBCVs. The *Brucella* VirB T4SS has been long known to mediate rBCV biogenesis via delivery of specific effectors (Comerci *et al*, 2001; Celli *et al*, 2003, 2005; de Barsy *et al*, 2011; Döhmer *et al*, 2013; Miller *et al*, 2017; Smith *et al*, 2020). Recent progress in our understanding of T4SS effector functions has reinforced the concept that *Brucella* exploits membrane trafficking pathways between the ER and the Golgi apparatus to generate its replicative vacuole, with RicA‐ and BspB‐modulating Golgi‐to‐ER retrograde transport via targeting of the GTPase Rab2 and the Golgi‐associated COG complex, respectively (de Barsy *et al*, 2011; Miller *et al*, 2017; Smith *et al*, 2020). Our findings on BspF’s function broaden the scope of *Brucella*’s exploitation of secretory functions by revealing a role of post‐Golgi transport steps in the bacterium’s intracellular cycle. Uncovering the specific function of BspF in intracellular bacterial replication also substantiates a role of *Brucella*’s VirB T4SS as directly promoting intracellular bacterial proliferation, consistent with recent evidence suggesting that an active VirB T4SS is required during bacterial replication within rBCVs (Smith *et al*, 2016).

We show that ectopically expressed or T4SS‐delivered BspF intrinsically targets ACAP1, resulting in decreased ACAP1–Arf6 interactions and total active GTP‐bound Arf6. Together with the localization of BspF to the tubular RE and the rescue of BspF’s function in bacterial replication by either overexpression of ACAP1, a dominant inactive allele of Arf6 (Arf6^T27N^), or depletion of Arf6, these findings argue that BspF inhibits Arf6 activity on RE membranes via modulation of ACAP1 to fulfill its function. Given that Arf6 promotes activation of Rab8a on RE membranes via recruitment of the endocytic adapter MICAL‐L1 (Hattula *et al*, 2006; Rahajeng *et al*, 2012) to initiate membrane transport at the RE, we propose that BspF’s interference with Arf6 activity impairs Rab8a‐dependent transport, resulting in inhibition of post‐TGN exocytic transport via the RE and of RE‐TGN retrograde transport. Since recruitment of TGN‐associated vesicles to rBCVs also depends upon the Arf6/Rab8a axis and BspF, we infer that BspF‐mediated interference with this GTPase cascade results in increased availability of TGN‐derived, Stx6‐positive vesicles and their recruitment to rBCVs (Fig 6I). Reduced TGN‐associated vesicles recruitment to rBCVs via either *bspF* deletion or Arf6 or Rab8a depletions correlated with decreased bacterial replication in rBCVs, arguing that interception of TGN‐derived traffic by rBCVs promotes intravacuolar bacterial growth. This model is further supported by the findings that (i) Arf6, Rab8a, and Stx6 functions are important for *Brucella* intracellular replication and (ii) inhibition of Arf6/Rab8a‐ or Stx6‐dependent transport pathways could functionally rescue the replication defect of a BspF‐deficient mutant. By contrast, inactivation of Rab6a/a′‐dependent transport pathways affected replication and TGN‐associated vesicle recruitment, but also rBCV biogenesis, and did not rescue replication of ∆*bspF* bacteria. Additionally, Rab6a/a′ depletion failed to rescue replication of the ∆*bspF* mutant, which further supports the specificity of BspF’s effect toward the Arf6/Rab8a regulatory cascade in this pathway.

The molecular mechanism by which BspF enhances Arf6 inactivation via its interaction with ACAP1 remains to be determined. The rescue of the replication defect of a ∆*bspF* mutant by ACAP1 overexpression in macrophages suggests that BspF enhances ACAP1 GAP activity to inactivate Arf6, which is consistent with the failure of the GAP mutant ACAP1^R448Q^ to rescue the phenotype. Our original identification of the BspF–ACAP1 interaction indicates that BspF directly binds a C‐terminal region of ACAP1 that contains its GAP domain and Ankyrin (Ank) repeats, a region known to bind the PTB domain‐containing adaptor protein GULP that regulates ACAP1 activity on Arf6 (Ma *et al*, 2007). BspF’s targeting of this same region of ACAP1 argues that it may either compete with binding of ACAP1 regulators or directly alter its GAP activity. Bioinformatic analysis of BspF amino acid sequence predicts it carries a Gcn5‐associated N‐acetyl transferase (GNAT) domain (Pfam PF13480) within the amino acid residues 224–366 (Myeni *et al*, 2013). It is therefore possible that BspF acetylates ACAP1, a post‐translational modification that is known to regulate the membrane‐binding activity of ACAP4, another Arf6 GAP (Song *et al*, 2018), consistent with the loss of ACAP1 association with membrane tubules that we observed in the presence of BspF. Recent evidence that BspF influences crotonylation of host proteins (Zhu *et al*, 2021) also suggests that it may post‐translationally modulate ACAP1 and Arf6 functions.

How interception of TGN‐derived vesicular traffic by rBCVs via BspF promotes intravacuolar bacterial growth remains to be determined. *C. trachomatis* intercepts TGN‐derived vesicles to acquire and incorporate sphingolipids, a process that contributes to inclusion biogenesis and bacterial growth (Hackstadt *et al*, 1996; Scidmore *et al*, 1996; van Ooij *et al*, 2000; Robertson *et al*, 2009; Capmany & Damiani, 2010). *Legionella*‐containing vacuoles also intercept TGN‐derived membranes during their maturation (Weber *et al*, 2018), but whether these events contribute to bacterial proliferation remains to be established. Similarly, BspF‐mediated redirection of TGN transport might provide intravacuolar *Brucella* with specific lipids or other nutrients that facilitate their growth, a hypothesis to be tested in future studies. Uropathogenic *Escherichia* coli (UPEC) remodels RE traffic to acquire transferrin‐bound iron and promote its intracellular growth within vacuoles (Dikshit *et al*, 2015). Given the role of Stx6 in RE‐TGN retrograde transport, it remains possible that the Stx6‐positive vesicles recruited to rBCVs also originate from the RE and may supply micro‐nutrients such as iron to intravacuolar *Brucella*, which may be captured within rBCVs by the bacterial iron acquisition systems (Roop, 2012).

Modulation of transport pathways between endosomal compartments and the TGN is a common pathogenic strategy that serves several intracellular microbes, although with diverse roles such as vacuolar trafficking and integrity, nutrient acquisition, and intracellular survival (Personnic *et al*, 2016). *S. enterica* Typhimurium T3SS effector SifA modulates kinesin‐mediated vesicular traffic to inhibit Rab9‐dependent retrograde transport of mannose 6‐phosphate receptors from late endosomes and impair lysosomal bactericidal capabilities (McGourty *et al*, 2012), thereby enhancing bacterial intravacuolar survival. *S*. Typhimurium T3SS effector SseC binds the retromer complex to promote SCV integrity (Patrick *et al*, 2018), although how retrograde transport contributes to vacuolar maintenance is unknown. The *L. pneumophila* Dot/Icm T4SS effector RidL and the *C. trachomatis* T3SS effector IncE bind the retromer complex components Vps29 (Finsel *et al*, 2013; Bärlocher *et al*, 2017; Romano‐Moreno *et al*, 2017; Yao *et al*, 2018) and SNX1/2/5/6 (Mirrashidi *et al*, 2015), respectively, to inhibit its activity and counteract its restrictive functions on bacterial intracellular growth (Finsel *et al*, 2013; Mirrashidi *et al*, 2015). Yet, how retrograde transport restricts these two pathogens remains to be understood. Conversely, retromer functions are necessary to the biogenesis of the intracellular vacuoles of *C. burnetii* and *B. abortus* (McDonough *et al*, 2013; Casanova *et al*, 2019), although the underlying mechanisms of these requirements are unknown. The role of the retromer in rBCV biogenesis argues for a direct role of retrograde transport in the *Brucella* intracellular cycle, but it may also reflect the need for functional integrity of the endosomal/lysosomal system for rBCV biogenesis, given the retromer’s functional connection with Rab7 (Casanova *et al*, 2019). Our findings that BspF alters Arf6/Rab8a‐dependent retrograde transport to promote bacterial growth within rBCVs are likely unrelated to this described role of the retromer, because BspF promotes bacterial growth and not rBCV biogenesis. Hence, our findings constitute a distinct example of bacterial subversion of endosome‐TGN transport via the endosomal recycling compartment by targeting of a GTPase regulatory cascade, for the purpose of intracellular proliferation.

## Materials and Methods

### Reagents and Tools table

**Table:** 

Reagent/Resource	Reference or Source	Identifier or Catalog Number
**Experimental Models**		
*Brucella abortus 2308::miniTn7K‐dsRed*	Miller *et al*, 2017	N/A
*Brucella abortus* 2308 ∆*bspF::miniTn7K‐dsRed*	This study	N/A
*Brucella abortus* 2308 ∆*bspF::miniTn7K‐dsRed‐bspF*	This study	N/A
*Brucella abortus* 2308 ∆*bspB::miniTn7K‐dsRed*	Miller *et al*, 2017	N/A
*Brucella abortus* 2308 ∆*bspB::miniTn7K‐dsRed‐bspB*	Miller *et al*, 2017	N/A
*Brucella abortus* 2308 ∆*virB11::miniTn7K‐dsRed*	Smith *et al*, 2016	N/A
*Homo sapiens*: embryonic kidney 293T cells (HEK293T/17)	ATCC	Cat#CRL‐11268; RRID:CVCL_1926
*Homo sapiens*: HeLa cells (CCL‐2)	ATCC	Cat#CCL‐2; RRID:CVCL_0030
*Homo sapiens*: HeLa‐M clone 1 (C1) cells	Gordon *et al*, 2010	N/A
*M. musculus*: Bone Marrow Derived Macrophages (BMMs) from C57BL/6J mice	The Jackson Laboratory	Cat#000664
**Recombinant DNA**		
*Indicate species for genes and proteins when appropriate*		
pUC18T‐miniTn7K‐*dsRed*‐*bspF* *Brucella abortus*	This study	N/A
pUC18T‐miniTn7K‐*dsRed*	Smith *et al*, 2016	N/A
pUC18T‐Tn7‐*tnp*	Dr M Kovach; Myeni *et al*, 2013	N/A
pcDNA3‐*arf6*‐HA *Homo sapiens*	This study	N/A
pCMV‐HA	Clontech	Cat#635690
pCMV‐HA‐*bspF* *Brucella abortus*	Myeni *et al*, 2013	N/A
pCMV‐Myc	Clontech	Cat#631604
pCMV‐myc‐*ACAP1* *Homo sapiens*	This study	N/A
pmCherry‐C1	Clontech	Cat#632524
pmCherry‐N1‐*arf6* ^T27N^ *Homo sapiens*	This study	N/A
pmCherry‐*bspF* *Brucella abortus*	This study	N/A
pmCherry‐*rab6a*′^T27N^ *Homo sapiens*	This study	N/A
pmCherry‐*rab8a* ^T22N^ *Homo sapiens*	This study	N/A
pEGFP‐*ACAP1* *Homo sapiens*	This study	N/A
pEGFP‐N1‐*arf6* *Homo sapiens*	This study	N/A
pEGFP‐N1‐*arf6* ^Q67L^ *Homo sapiens*	This study	N/A
pEGFP‐N1‐*arf6* ^T27N^ *Homo sapiens*	This study	N/A
pEGFP‐*MICAL‐L1* *Homo sapiens*	This study	N/A
pEGFP‐r*ab6a* *Homo sapiens*	This study	N/A
pEGFP‐r*ab6a*′ *Homo sapiens*	This study	N/A
pEGFP‐*rab8a* *Homo sapiens*	Hattula & Peränen, 2000	N/A
pEGFP‐*rab11a* *Homo sapiens*	This study	N/A
pEGFP‐s*tx6* *Rattus norvegicus*	Kudlyk *et al*, 2013	N/A
pEGFP‐s*tx16* *Homo sapiens*	Willett *et al*, 2013	N/A
pEGFP‐*TGN38*	This study	N/A
pEGFP‐*VAMP3* *Homo sapiens*	Addgene	Cat#42310
pEGFP‐*VAMP4* *Homo sapiens*	Addgene	Cat#42313
pCLXSN‐MCS2	Miller *et al*, 2017	N/A
pCLXSN‐MCS2‐GFP	Miller *et al*, 2017	N/A
pCLXSN‐MCS2‐*GFP‐ACAP1* *Homo sapiens*	This study	N/A
pCLXSN‐MCS2‐*GFP‐ACAP1(R448Q)* *Homo sapiens*	This study	N/A
pCLXSN‐MCS2‐*arf6(Q67L)‐GFP* *Homo sapiens*	This study	N/A
pCLXSN‐MCS2*‐arf6(T27N)‐GFP* *Homo sapiens*	This study	N/A
pCLXSN‐MCS2‐*GFP‐bspF* *Brucella abortus*	This study	N/A
pCLXSN‐MCS2‐mCherry	This study	N/A
pCLXSN‐MCS2‐*arf6^Q67L^‐mCherry* *Homo sapiens*	This study	N/A
pCLXSN‐MCS2‐*arf6^T27N^‐mCherry* *Homo sapiens*	This study	N/A
pCL‐Eco	Imgenex, Novus Biologicals	Cat#NBP2‐29540
**Antibodies**		
Mouse monoclonal anti‐β‐Actin (clone 8H10D10) (1:20,000)	Cell Signaling Technology^®^	Cat#3700S; Lot#18; RRID:AB 2227609
Rabbit monoclonal anti‐Arf6 (clone D12G6) (1:10,000)	Cell Signaling Technology^®^	Cat#5740S, Lot#1; RRID:AB_10694539
Rabbit polyclonal anti‐Calnexin (IF 1:1,000) (WB 1:20,000)	Enzo Life Sciences, Inc.	Cat#ADI‐SPA‐860‐D; Lot#12301304; RRID:AB_10616095
Mouse monoclonal anti‐ERGIC‐53 (G1/93) (1:200)	Alexis® Biochemicals, Enzo Life Sciences, Inc.	Cat#ALX‐804‐602‐C100; RRID:AB_2051363
Mouse monoclonal anti‐GM130 (1:500)	BD Transduction Laboratories™, BD Biosciences	Cat#610823; Lot#07536; RRID:AB_398142
Rabbit monoclonal anti‐HA (clone C29F4) (1:10,000)	Cell Signaling Technology^®^	Cat#3724S, Lot#9; RRID:AB_1549585
Rat monoclonal anti‐HA (3F10) (1:500)	Roche	Cat#1867423; RRID:AB_390918
Mouse monoclonal anti‐Hsp27 (G31) (1:10,000)	Cell Signaling Technology^®^	Cat#2402; Lot#8; RRID:AB_331761
Rabbit polyclonal anti‐Lamin A/C (1:5,000)	Cell Signaling Technology^®^	Cat#2032; Lot#5; RRID:AB_2136278
Rat monoclonal anti‐LAMP1 (1D4B) (1:400)	Developmental Studies Hybridoma Bank, University of Iowa	Cat#1d4b; RRID:AB_2134500
Rabbit monoclonal anti‐Myc (clone 71D10) (1:10,000)	Cell Signaling Technology^®^	Cat#2278S, Lot#5; RRID:AB_490778
Mouse monoclonal anti‐p230 (1:100)	BD Transduction Laboratories, BD Biosciences	Cat#611280; RRID:AB_398808
Rabbit monoclonal anti‐Rab6 (clone D37C7) (1:1,000)	Cell Signaling Technology^®^	Cat#9625S, Lot#1; RRID:AB_10971791
Rabbit monoclonal anti‐Rab8a (clone D22D8) (1:1,000)	Cell Signaling Technology^®^	Cat#6975S, Lot#2; RRID:AB_10827742
Rabbit monoclonal anti‐Syntaxin 6 (clone C34B2) (IF 1:100) (WB 1:1,000)	Cell Signaling Technology^®^	Cat#2869S, Lot#6; RRID:AB_2196500
Peroxidase AffiniPure Goat anti‐Mouse IgG (1:10,000)	Jackson Laboratories	Cat#115‐035‐071
Peroxidase AffiniPure Goat anti‐Rabbit IgG (1:10,000)	Jackson Laboratories	Cat#111‐035‐144
Alexa Fluor™ 488‐conjugated donkey anti‐rabbit (1:500)	Invitrogen, ThermoFisher, Scientific	Cat#A21208, Lot#1810450
Alexa Fluor™ 647‐conjugated donkey anti‐rat (1:500)	Molecular Probes^®^, ThermoFisher, Scientific	Cat#A21208; Lot#1810450
Alexa Fluor™ 647‐conjugated phalloidin (1:250)	Molecular Probes^®^, ThermoFisher, Scientific	Cat#A22287; Lot#1246069
**Oligonucleotides and other sequence‐based reagents**		
RC247‐*bspF* pGBKT7 Forward: 5′‐GATGGAGGCCGAATTCGCTGCAAAACCTTTGCTTGAGG‐3′	This study	N/A
RC248‐*bspF* pGBKT7 Reverse: 5′‐GCAGGTCGACGGATCCTTTATGCTCGGTGAAACTGCG‐3′	This study	N/A
RC603‐miniTn*7*K‐*dsRed* Forward: 5′‐ATCATCCTCATCACCGACAA‐3′	Myeni *et al*, 2013	N/A
RC604‐miniTn*7*K‐ds*Red* Reverse: 5′‐GCTATATTCTGGCGAGCGAT‐3′	Myeni *et al*, 2013	N/A
WSU0141‐*ACAP1* Reverse *Bam*HI: 5′‐TATTGGATCCTATTACAGCGTGTGGAGGTCATG‐3′	This study	N/A
WSU0165‐Mutagenesis ACAP1 R448Q Forward: 5′‐GTTCCGGCATCCACCAGAGCCTTGGTGTTC‐3′	This study	N/A
WSU0166‐Mutagenesis ACAP1 R448Q Reverse: 5′‐GAACACCAAGGCTCTGGTGGATGCCGGAAC‐3′	This study	N/A
WSU0218‐*bspF* Tn*7*K Forward: 5′‐AGCTCGAATTCACCATCTTCCGATCTTGGCTG‐3′	This study	N/A
WSU0219‐*bspF* Tn*7*K *Kpn*I Reverse: 5′‐AAGGTACCTTATTTATGCTCGGTGAAACTGC‐3′	This study	N/A
WSU0221‐*bspF* pmCherry Forward *Bgl*II: 5′‐CTCAGATCTGCTGCAAAACCTTTGCTTGAGG‐3′	This study	N/A
WSU0222‐*bspF* pmCherry Reverse *Kpn*I: 5′‐CGCGGTACCTTATTTATGCTCGGTGAAACTGC‐3′	This study	N/A
WSU0247‐*eGFP* Forward *Eco*RI: 5′‐TAGCGGAATTCGTCGCCACCATGGTGAGC‐3′	This study	N/A
WSU0248‐*rab6a* Reverse *Bam*HI: 5′‐CGGGATCCGTTAGCAGGAACAGCCTCC‐3′	This study	N/A
WSU0258‐*rab6a* T27N Forward: 5′‐CAAAGCGTTGGAAAGAATTCTTTGATCACCAGA‐3′	This study	N/A
WSU0259‐*rab6a* T27N Reverse: 5′‐TCTGGTGATCAAAGAATTCTTTCCAACGCTTTG‐3′	This study	N/A
WSU0350‐pEGFP‐N1 *BamHI* Reverse: 5′‐CGCGGATCCGCTTTACTTGTACAGCTCGTCCATG‐3′	This study	N/A
WSU0353‐pEGFP C1 Reverse: 5′‐TGATCAGTTATCTAGATCCGGTGG‐3′	Miller *et al*, 2017	N/A
WSU0354‐pEGFP C1 Forward *Cla*I: 5′‐TAATATCGATGCCACCATGGTG‐3′	Miller *et al*, 2017	N/A
WSU0433‐*arf6* Forward *Eco*RI: 5′‐ATAAGAATTCACCATGGGGAAGGTGCTA‐3′	This study	N/A
WSU0435‐*ACAP1* Forward *Sal*I: 5′‐TTAGTCGACATGACGGTCAAGCTGGATTTCG‐3′	This study	N/A
WSU0437‐*arf6* Reverse *Kpn*I: 5′‐GAGGTACCAGAGATTTGTAGTTAGAGGTTAACC‐3′	This study	N/A
WSU0438‐*rab11a* Forward *EcoRI*: 5′‐ ATTAGAATTCTATGGGTACCCGCGAC‐3′	This study	N/A
RC451‐*ACAP1* pCMV Forward *Eco*RI: 5′‐ATGGAGGCCCGAATTCAAACGGTCAAGCTGGATTTCGAG‐3′	This study	N/A
RC452‐*ACAP1* pCMV Reverse *Eco*RI: 5′‐TCGGTCGACCGAATTCTTACAGCGTGTGGAGGTCATG‐3′	This study	N/A
RC453‐*acap1* pGADT7 Forward: 5′‐GGAGGCCAGTGAATTCACGGTCAAGCTGGATTTCGAG‐3′	This study	N/A
RC464‐*acap1* pGADT7 Reverse: 5′‐CGAGCTCGATGGATCCCAGCGTGTGGAGGTCATG‐3′	This study	N/A
WSU0620‐*MICAL‐L1* Forward *HindIII*: 5′‐ TAGTAAGCTTATATGGCTGGGCCG‐3′	This study	N/A
WSU621‐*MICAL‐L1* Reverse *SalI*: 5′‐ATTGTCGACTTAGCTCTTGTCTCTGG‐3′	This study	N/A
TW770‐*bspF* pEGFP‐C1 Forward *Bgl*II: 5′‐GTCCGGACTCAGATCTGCTGCAAAACCTTTGCTTGA‐3′	Myeni *et al*, 2013	N/A
TW771‐*bspF* pEGFP‐C1 Reverse *Bgl*II: 5′‐CTTGAGCTCGAGATCTTTATTTATGCTCGGTGAAACTGCG‐3′	Myeni *et al*, 2013	N/A
siRNA targeting sequence: ON‐TARGETplus Mouse Arf6 siRNA ‐ SMARTpool: CAAACGGGGUGGGGUAAUA, CUGACAUUUGACACGAAUA, CGGCAUUACUACACCGGGA, GGGUCUCAUCUUCGUGGUA	Dharmacon™	Cat#L‐043217‐01‐0005
siRNA targeting sequences: ON‐TARGETplus Non‐targeting Pool: UGGUUUACAUGUCGACUAA, UGGUUUACAUGUUGUGUGA, UGGUUUACAUGUUUUCUGA, UGGUUUACAUGUUUUCCUA	Dharmacon™	CAT# D‐001810‐10‐20
siRNA targeting sequence: ON‐TARGETplus Mouse Rab6a siRNA ‐ SMARTpool: GGGCGGAGACUUCGGGAAU, UCAGAGGAAGUAUGCAUUA, GCACUUGGAUUAUGGAUCU, UCGUGGAGGUGAUGUAUUA	Dharmacon™	CAT#L‐040858‐01‐0005
siRNA targeting sequences: ON‐TARGETplus Mouse Rab8a siRNA ‐ SMARTpool: CAGGAGCGGUUUCGAACAA, GUAUCAUGCUGGUCUACGA, CAGAAGGUAGCCAGCGGUA, CGGACUCGAUUCACAAAUU	Dharmacon™	CAT#L‐040860‐01‐0005
siRNA targeting sequences: ON‐TARGETplus Mouse Stx6 siRNA ‐ SMARTpool: GCACAUCUAUUACGCUUAU, CAUCACAAGUACUCGGCAA, CUGGAGUGGCAGAUCGCUA, AGAACAUGUCGCAGCGCAU	Dharmacon™	CAT#L‐059391‐01‐0005
**Chemicals, Enzymes and other reagents**		
Cytochalasin D	Thermo Fisher	Cat#C8272‐1MG, Lot#067M4075V
AlexaFluor™488‐conjugated Cholera Toxin Subunit B	Invitrogen	Cat#C22841
Fetal Bovine Serum	Atlanta Biologicals	Cat#S10350H; Lot#J15105
Human epidermal growth factor (EGF)	EMD Millipore	Cat#01‐107
Normal horse serum	Gibco	Cat#16050‐130; Lot#1517706
FuGENE® 6	Promega	Cat#E2692
Rapamycin	LC Laboratories^®^	Cat#R‐5000; Lot#ASW‐125
Aureobasidin A	Takara Bio USA, Inc	Cat#630499
X‐a‐Gal	Takara Bio USA, Inc	Cat#630463
**Software**		
*Include version where applicable*		
Adobe® Photoshop^®^ CS6 software for Mac	Adobe Systems Incorporated, San Jose, California, USA	www.adobe.com/ products/photoshop; RRID:SCR_014199
CellProfiler4.0.7	Broad Institute, Cambridge, Massachusetts, USA	www.cellprofiler.org
GraphPad Prism^®^ version 9.0e for Mac	GraphPad Software, La Jolla, California, USA	www.graphpad.com; RRID:SCR_002798
Fiji ImageJ 2.1.0	Open source database, National Institute of Health	imageJ.nih.gov
MacVector version 12.7.5 for Mac	MacVector, Inc., Apex, North Carolina, USA	www.macvector.com
**Other**		
Matchmaker Gold Yeast Two‐Hybrid system	Takara Bio USA, Inc	Cat#630489
Mate & Plate human bone marrow cDNA library	Takara Bio USA, Inc	Cat#630477
Yeastmaker™ Yeast Transformation System 2	Takara Bio USA, Inc	Cat#630439
Easy Yeast Plasmid Isolation kit	Takara Bio USA, Inc	Cat#630467
Arf6 G‐LISA Activation Assay Kit (Colorimetric Based)	Cytoskeleton, Inc.	Cat#BK133
Mouse Macrophage Nucleofector® Kit	Lonza	Cat#VPA1009

### Methods ad Protocols

#### Bacterial strains and culture

*Brucella abortus* strains 2308, 2308∆*virB11*, 2308∆*bspB*, 2308∆*bspB::*mTn7K‐*bspB*, and 2308∆*bspF* have been described previously (Myeni *et al*, 2013; Smith *et al*, 2016) and were grown on tryptic soy agar (TSA, Difco™) for 3 days at 37°C and 5% CO_2_, and subsequently in tryptic soy broth (TSB, Difco™) at 37°C with shaking to an OD_600_˜0.8 for infections. For genetic complementation of *B. abortus* strain 2308Δ*bspF* via chromosomal insertion of a single copy of *bspF*, a 1626 bp DNA fragment containing *bspF* (BAB1_1948) and 340 bp upstream were PCR amplified from pUC18T‐miniTn7K‐*bspF* (Myeni *et al*, 2013) using primers WSU0218 and WSU0219 (Reagents and Tools table), and cloned into pUC18T‐miniTn7K*‐dsRed* using *Eco*RI and *Kpn*I restriction sites to generate pUC18T‐mini*Tn*7K*‐dsRed‐bspF*, which was electroporated into *B. abortus* 2308Δ*bspF* as described below. For fluorescence microscopy purposes, all strains were modified to express the fluorescent protein DsRed_m_ via integration of the miniTn7K*‐dsRed* at the *attTn*7 locus by electroporation of either pUC18T‐miniTn7K*‐dsRed* (Smith *et al*, 2016), pUC18T‐miniTn7K‐*dsRed‐bspB* (Miller *et al*, 2017), or pUC18T‐miniTn7K‐*dsRed‐bspF* with the helper plasmid pUC18T‐Tn7*tnp*, as described previously (Myeni *et al*, 2013). Electroporants were selected on TSA plates containing 30 μg/ml of kanamycin, and correct insertion of mini*Tn7K‐dsRed* was confirmed using PCR primers RC603 and RC604 (Reagents and Tools table). All experiments with *B. abortus* strains were performed in a Biosafety Level 3 facility in compliance with the CDC/USDA Federal Select Agents Program regulations in accordance with standard operating procedures approved by Washington State University Institutional Biosafety Committee. *Escherichia coli* strains used for cloning (DH10B, DH5α: Invitrogen) were grown in Luria‐Bertani (Difco™ LB Lennox, BD) broth at 37°C, supplemented with 50 μg/ml of kanamycin or 100 μg/ml of ampicillin (Thermo Fisher Scientific) when necessary.

#### Mammalian cells

Human embryonic kidney 293T cells (HEK293T/17; ATCC CRL‐11268) were grown in Dulbecco’s Modified Eagle's Medium (DMEM 4.5 g/l glucose and sodium pyruvate; Corning Cat#15013) supplemented with 10% heat‐inactivated fetal bovine serum (FBS; Gibco, Life Technologies) and 4 mM l‐glutamine at 37°C and 10% CO_2_. HeLa cells (ATCC clone CCL‐2) were cultured in Minimum Essential Medium (MEM; Corning Cat#15010) supplemented with 2 mM l‐glutamine and 10% FBS and grown at 37°C in 5% CO_2_. HeLa‐M clone 1 (C1) cells (Gordon *et al*, 2010) were grown in 4.5 g/l glucose DMEM supplemented with 10% FBS, 2 mM l‐glutamine and 1.66 μg/ml puromycin (Thermo Fisher Scientific) at 37°C and 5% CO_2_. Murine bone marrow‐derived macrophages (BMMs) were generated from bone marrow cells collected from 6‐ to 12‐week‐old female C57BL/6J mice (Jackson) and differentiated into macrophages for 5 days at 37°C and 10% CO_2_ in 1 g/l glucose Dulbecco’s Modified Eagle's Medium (DMEM with l‐glutamine, and sodium pyruvate, Corning Cat#10014) supplemented with 10% fetal bovine serum (FBS, Invitrogen) and 20% L‐929 mouse fibroblasts‐conditioned medium (L‐CSF) in non‐tissue culture‐treated Petri dishes (Corning). After 5 days, adherent BMMs were washed with PBS, harvested by incubation in chilled cation‐free PBS (Corning) supplemented with 1 g/l D‐glucose on ice for 10 min, pelleted by centrifugation, and resuspended in complete medium (DMEM, 10% FBS, and 10% L‐CSF) and replated in either 6‐ or 24‐well tissue culture‐treated plates (Cellstar ® greiner bio‐one) at a density of 1 × 10^6^ or 5 × 10^4^ cell/well, respectively. BMMs were further incubated at 37°C under 10% CO_2_ atmosphere for 48 h, replenishing with complete medium 24 h before infection.

#### Construction of mammalian expression plasmids and retroviral vectors

All PCR primers referred to are described in the Reagents and Tools table. To generate pmCherry‐C1‐Rab6a′^T27N^, *rab6a* cDNA was amplified from pEGFP‐C1‐Rab6a (Clontech) using primers WSU0247 and WSU0248, digested with *Xho*I and *Bam*HI and ligated into pmCherry‐C1 (Clontech). Site‐directed mutagenesis of human *rab6a* cDNA was carried out by PCR using primers WSU0258 and WSU0259 and *Dpn*I digestion of the template plasmid. Subsequently, the variable region of human *rab6a* ^T27N^/*rab6a*′^T27N^ cDNA was swapped by digestion of pmCherry‐C1‐Rab6a^T27N^ and pDONR201‐Rab6a′^T27N^ (Addgene #44702) using *Eco*RI and *Eco*RV restriction sites and ligation of the *rab6a*′^T27N^ fragment into pmCherry‐C1‐Rab6a^T27N^ to generate pmCherry‐C1‐Rab6a′^T27N^. Dominant negative human Rab8a^T22N^ was digested from pEGFP‐C1‐Rab8a^T22N^ (Hattula & Peränen, 2000) using *Eco*RI and *Bam*HI restriction sites and ligated into pmCherry‐C1 (Clontech) to generate pmCherry‐Rab8a^T22N^. Wild‐type, constitutively active, and dominant negative human Arf6, Arf6^Q67L^, and Arf6^T27N^ were amplified from pcDNA3‐HA‐Arf6 (Addgene #10834), pcDNA3‐HA‐Arf6^Q67L^ (Addgene #10835), or pcDNA3‐HA‐Arf6^T27N^ (Addgene #10831) using primers WSU0433 and WSU0437 and cloned into either pEGFP‐N1 or pmCherry‐N1 (Clontech) using *Eco*RI and *Kpn*I restriction sites to generate pmEGFP‐N1‐Arf6, pEGFP‐N1‐Arf6^Q67L^, pEGFP‐N1‐Arf6^T27N^or pmCherry‐N1‐Arf6, pmCherry‐N1‐Arf6^Q67L^, and pmCherry‐N1‐Arf6^T27N^. Subsequently, Arf6‐GFP alleles were amplified using primers WSU0433 and WSU0350 and cloned into pCLXSN‐MCS2 using *EcoRI* and *BamHI* restriction sites to generate pCLXSN‐Arf6‐GFP, pCLXSN‐Arf6^Q67L^‐GFP, and pCLXSN‐Arf6^T27N^‐GFP. Arf6‐mCherry alleles were amplified using primers WSU0433 and WSU0336 and cloned into pCLXSN‐MCS2 using *EcoRI* and *KpnI* restriction sites to generate pCLXSN‐Arf6‐mCherry, pCLXSN‐Arf6^Q67L^‐mCherry, and pCLXSN‐Arf6^T27N^‐mCherry. Human ACAP1 cDNA was originally amplified from pCMV6‐AC‐GFP‐ACAP1 (OriGene #RG203724) using primers RC451 and RC452 and cloned into pCMV‐myc (Clontech) using the *Eco*RI restriction site to generate pCMV‐myc‐ACAP1. *ACAP1* cDNA was then amplified from pCMV‐myc‐ACAP1 using primers WSU0435 and WSU0141 and cloned into pEGFP‐C1 (Clontech) using *Sal*I and *Bam*HI restriction sites to generate pEGFP‐C1‐ACAP1. To generate the ACAP1 GAP‐inactive mutant (Jackson *et al*, 2000), human ACAP1 cDNA was amplified from pCMV6‐AC‐GFP‐ACAP1 (OriGene #RG203724) using primers RC453 and RC464 and cloned into pGADT7 using the *Eco*RI and *Bam*HI restriction sites to generate pGADT7‐ACAP1_FL_. Site‐directed mutagenesis of *pGADT7‐ACAP1* was carried out by PCR using primers WSU0165 and WSU0166 and *Dpn*I digestion of the template plasmid. *ACAP1^R448Q^* was amplified from pGADT7‐ACAP1^R448Q^ using primers WSU0435 and WSU0141 and cloned into pEGFP‐C1 using *SalI* and *BamHI* restriction sites. The resulting *GFP‐ACAP1* and *GFP‐ACAP1^R448Q^* fragments were then amplified using primers WSU0354 and WSU0141 and cloned into pCLXSN‐MCS2 (Miller *et al*, 2017) using *Bam*HI and *Cla*I restriction sites to generate pCLXSN‐GFP‐ACAP1 and pCLXSN‐GFP‐ACAP1^R448Q^. Human MICAL‐L1 was amplified from pENTR223‐MICAL‐L1 (DNASU HsCD00073683) using primers WSU0620 and WSU0621 and cloned into pEGFP‐C1 (Clontech) using *Hin*dIII and *Sal*I restriction sites to generate pEGFP‐C1‐MICAL‐L1. Human *rab11a* was amplified from pEGFP‐C1‐2xHA‐Rab11a (Addgene #12674) using primers WSU0353 and WSU0438 and cloned into pEGFP‐C1 (Clontech) using *Eco*RI and *Bam*HI restriction sites to generate pEGFP‐C1‐Rab11a. *BspF* was amplified from pCMV‐HA‐*bspF* (Myeni *et al*, 2013) using primers WSU0221 and WSU0222 and cloned into pmCherry‐C1 (Clontech) using *Bgl*II and *Kpn*I restriction sites to generate pmCherry‐C1‐BspF. *BspF* was amplified from *B. abortus* (strain 2308) genomic DNA with primers TW770 and TW771 using *Bgl*II restriction sites and cloned into pEGFP‐C1. The resulting *GFP‐bspF* fragment was amplified from pEGFP‐C1‐*bspF* using primers WSU0353 and WSU0354 and cloned into pCLXSN‐MCS2 using *Cla*I and *Sal*I restriction sites to generate pCLXSN‐GFP‐*bspF*. pCLXSN‐GFP was previously described (Miller *et al*, 2017). All constructs were confirmed by DNA sequencing.

#### siRNA treatment and retroviral transduction of BMMs

After 5 days of differentiation, BMMs were collected and 1 × 10^6^ cells electroporated with 2–3 μM ON‐TARGETplus SMARTpool siRNAs (GE Dharmacon) directed against either mouse Arf6 (L‐043217‐01‐0005), mouse Rab8a (L‐040860‐01‐0005), mouse Rab6a/a′ (L‐040858‐01‐0005), mouse Stx6 (L‐059391‐01‐0005), or a non‐targeting (siNT; D‐001810‐10‐20) siRNA, in an Amaxa^TM^ Nucleofector II using the Mouse Macrophage Nucleofector^®^ Kit (Lonza). BMMs were immediately diluted in pre‐warmed medium, plated either onto coverslips in a 24‐well plate, or in a 6‐well plate, and incubated for 72 h prior to infection. Protein depletions were evaluated by Western blotting and densitometric analysis using β‐actin levels for normalization. Retroviral transductions of BMMs were performed using derivatives of pCLXSN and the ecotropic helper plasmid pCL‐Eco (Retromax, Imgenex). Retroviral supernatants were generated as follows: HEK 293T cells were seeded in 10 cm tissue culture dishes at 2.5 × 10^6^ in 20 ml medium and transfected after 24 h with a mix of 800 µl DMEM, 8 µg pCL‐Eco, 8 µg pCLXSN derivative, and 48 µl FuGENE^®^ 6 (Roche) following the manufacturer's protocol and incubated for 48 h prior to collection. Retroviral supernatants filtered through a 0.45 μm filter were added to BMMs (2:5 ratio v/v), and retroviral transduction proceeded for 24 h before BMM infections was performed.

#### Infection of macrophages

Bacterial cultures in early logarithmic phase of growth were diluted in chilled macrophage tissue culture medium [1 g/l glucose DMEM with l‐glutamine and sodium pyruvate (Corning Cat#10014) supplemented with 10% FBS (Invitrogen) and 10% L‐929 mouse fibroblasts‐conditioned medium (L‐CSF)] and added to chilled BMMs at a multiplicity of infection (MOI) of 10. Bacteria were centrifuged onto cells at 400 × *g* for 10 min at 4°C and incubated for 20 min at 37°C for BMM. Infected cells were then washed five times with DMEM to remove extracellular bacteria, then treated with 100 μg/ml of gentamicin (Gibco) in macrophage medium between 1 and 2 h pi to kill extracellular bacteria, after which gentamicin was omitted for the remainder of the experiment.

#### Antibodies

For immunofluorescence, antibodies used were rabbit monoclonal anti‐Stx6, (1:100; Cell Signaling), rabbit polyclonal anti‐Calnexin (1:1,000; Enzo Life Sciences), mouse monoclonal anti‐GM130 (1:500; BD Biosciences, 610822), mouse monoclonal anti‐p230 (1:100; BD Transduction Laboratories), mouse monoclonal anti‐ERGIC‐53 (1:200; Alexis^®^ Biochemicals ALX‐804‐602), rat monoclonal anti‐LAMP1 (1:400; clone 1D4B) (obtained from the Developmental Studies Hybridoma Bank and developed under the auspices of the NICHD and maintained by The University of Iowa, Department of Biological Sciences, Iowa City, IA 52242), rat monoclonal anti‐HA (1:500; Sigma‐Aldrich, 3F10), and Alexa Fluor™ 647 Phalloidin (1:250; Invitrogen™).

For Western blotting, primary antibodies used were rabbit monoclonal anti‐Arf6 (1:1,000; Cell Signaling), anti‐Rab8a (1:1,000; Cell Signaling), anti‐Rab6 (1:250; Cell Signaling), anti‐Stx6 (1:1,000; Cell Signaling), rabbit polyclonal anti‐Calnexin (1:20,000; Stressgen), anti‐Lamin A/C (1:5,000; Cell Signaling), anti‐β‐actin (1:20,000; Cell Signaling) antibodies; rabbit monoclonal anti‐HA, (1:10,000; Cell Signaling), anti‐myc (1:10,000; Cell Signaling), anti‐Hsp27 (1:10,000; clone G31, Cell Signaling) antibodies. Secondary antibodies used were AffiniPure goat anti‐rabbit IgG HRP (1:10,000; Jackson Laboratories, 111‐035‐144) and AffiniPure Goat anti‐mouse IgG (1:10,000; Jackson Laboratories, light chain‐specific 115‐005‐174).

#### Immunofluorescence microscopy

Mammalian cells seeded onto 12 mm glass coverslips were processed for immunofluorescence staining as follows: when necessary, endosomal tubule formation was induced by treating cells with pre‐warmed media containing 200 nM Cytochalasin D (Sigma‐Aldrich) and incubated at 37°C, 5% CO_2_ for 30 min prior to fixation. Coverslips were then washed three times in 1× PBS and then fixed in 3% paraformaldehyde (EMD) in 1× PBS for 20 min at 37°C. Samples were then washed three times with 1× PBS, and free aldehydes quenched in 50 mM ammonium chloride in PBS for 30 min at room temperature. Samples were blocked and permeabilized for 30 min in 0.1% saponin (w/v), 10% normal horse serum (v/v), 1× PBS, and then incubated for 20 min to 1 h with primary antibodies diluted in permeabilization buffer at room temperature, except for anti‐Stx6 incubations that were performed overnight at 4°C. Samples were washed in 0.1% saponin/PBS, then 1× PBS, and incubated for 30 min with either Alexa Fluor™ 488‐conjugated donkey anti‐mouse IgG, anti‐rat IgG, and Alexa Fluor™ 568‐conjugated donkey anti‐mouse IgG or anti‐rabbit IgG antibodies (1:500; Invitrogen, Life Technologies) at room temperature. Coverslips were washed in PBS, then rinsed in distilled H_2_O, and mounted on glass slides in Mowiol (Calbiochem). Samples were viewed with a Leica DM4000 epifluorescence upright microscope for quantitative analysis or a Leica SP8 confocal laser‐scanning microscope for image acquisition. Representative confocal micrographs of 1,024 × 1,024 pixels were acquired and assembled using Adobe Photoshop CC 2021. To quantify localization of host trafficking markers to BspF‐labeled tubules, HeLa cells expressing mCherry‐BspF and GFP‐tagged host proteins and treated with Cytochalasin D were analyzed manually and scored as positive if > 75% of mCherry‐BspF tubules in each cell analyzed were labeled with the GFP‐tagged marker. Three hundred cells were analyzed from three independent experiments. Colocalization of either GFP‐TGN38 or GFP‐ACAP1 with mCherry‐BspF was performed using the Fiji ImageJ (Fiji 2.1.0) Coloc_2 colocalization plug‐in. Parameters used were a Costes threshold regression with 100 randomizations and a point spread function (PSF) of 3.00. Ten cells/experiment were imaged by confocal microscopy and analyzed and data from three independent experiments were combined. Colocalization between GFP‐TGN38 and mCherry‐BspF was analyzed in 2 10.5 µm^2^ areas/cell that were selected in the mCherry channel as containing BspF‐positive structures and analyzed using Fiji ImageJ (Fiji 2.1.0) Coloc_2 colocalization plug‐in. GFP‐ACAP1 colocalization with mCherry‐BspF in Cytochalasin D‐treated cells was analyzed on whole cells. All measurements were presented as Pearson’s correlation coefficient (no threshold). Ten cells/experiment were analyzed, and data from three independent experiments were combined.

#### rBCV biogenesis and bacterial replication assays

To monitor rBCV biogenesis and intracellular replication of *Brucella* strains, BMMs infected with DsRed_m_‐expressing bacteria for various time points pi and processed for immunofluorescence staining of LAMP1 using a rat anti‐LAMP1 antibody (clone 1D4B) followed by Alexa Fluor™ 488‐conjugated donkey anti‐rat IgG. Coverslips were mounted blind on glass slides and analyzed by independent observers by epifluorescence microscopy. To assess rBCV biogenesis, percentages of LAMP‐1‐positive BCVs were scored at 4, 8, 12, and 24 h pi to evaluate the progressive exclusion of endosomal markers from BCV membranes as a readout of eBCV to rBCV conversion (Comerci *et al*, 2001; Celli *et al*, 2003; Salcedo *et al*, 2008; Starr *et al*, 2008, 2012; Miller *et al*, 2017; Smith *et al*, 2020). At least 100 BCVs were analyzed in each experiment, with experiments repeated independently at least three times. To assess bacterial replication in rBCVs, numbers of intracellular bacteria were scored at 24 h pi in all individual infected BMMs within a series of random fields (~100 BMMs analyzed/experiment). Each experiment was repeated independently at least 3 times.

#### Secretory trafficking assay

HeLa‐M (C1) cells expressing the secretory reporter protein ss‐eGFP‐FKBP^F36 M^ (Gordon *et al*, 2010) were transfected with pmCherry‐C1 control (Clontech) or pmCherry‐*bspF* for 24 h to allow for production of mCherry or mCherry‐BspF. Transfections were conducted following the FuGENE^®^ 6 manufacturer’s instructions (Roche). At 24‐h post‐transfection, rapamycin (200 nM, LC Laboratories^®^) was added to trigger solubilization, and initiate trafficking, of ss‐eGFP‐FKBP_F36 M_, which was followed over a 60‐min time course at 37°C and 5% CO_2_. Cells were PFA fixed and analyzed via immunofluorescence microscopy. To identify where ss‐eGFP‐FKBP^F36 M^ localized over time, the ER, ERGIC, Golgi apparatus, and trans‐Golgi network (TGN) were individually counterstained using anti‐Calnexin, anti‐ERGIC‐53, anti‐GM130, and anti‐p230 antibodies, respectively.

#### Yeast two‐hybrid screen

A yeast two‐hybrid screen to identify host proteins interacting with BspF was performed using the Matchmaker Gold Yeast Two‐Hybrid system (Takara #630489). A Gal4 BD fusion with BspF was created by cloning *bspF* in‐frame with *GAL4 BD* in the plasmid pGBKT7. The *bspF* open reading frame missing the START and STOP codons was amplified from *B. abortus* genomic DNA using primers RC247 and RC248 (Reagents and Tools table) and cloned into the *Eco*RI and *Bam*HI restriction sites of pGBKT7 using the In‐Fusion^®^ PCR Cloning System (Clontech, 639650) to generate pGBKT7‐*bspF*, which was verified by sequencing. pGBKT7‐*bspF* was then transformed into the Y2HGold yeast strain using the Yeastmaker Yeast Transformation System 2 kit (Clontech). A Mate & Plate human bone marrow cDNA library (Takara #630477) cloned into the yeast GAL4 activation domain (GAL4‐AD) vector pGADT7‐Rec and transformed into *S. cerevisiae* host strain Y187 was screened by mating with Y2HGold (pGBKT7‐*bspF*) as described by the manufacturer’s protocol. The positive control used was created by mating Y187‐containing pGADT7‐T (simian virus 40 large T antigen) with Y2HGold‐containing pGBKT7‐p53 (murine p53 protein). Y187 (pGADT7) mated with either Y2HGold (pGBKT7 vector) or Y2HGold (pGBKT7‐*bspF*) acted as the negative controls. Mated cultures were concentrated and plated in their entirety on synthetic minimal defined (SD) medium without Tryptophan or Leucine and supplemented with 5‐bromo‐4‐chloro‐3‐indolyl‐α‐D‐galactopyranoside (40 mg/ml; X‐α‐Gal) and 250 ng/ml Aureobasidin A (DDO/X/A plates). After 5–7 days, all colonies growing on the DDO/X/A plates were patched on quadruple dropout (QDO) medium (SD medium without Tryptophan, Leucine, Histidine, or Adenine and supplemented with X‐α‐Gal and Aureobasidin A (QDO/X/A)). Positive interactions were then confirmed by isolation and direct mating of Y2HGold (pGBKT7‐*bspF*) with the Y187 prey clones, whose plasmids were isolated using an Easy Yeast Plasmid Isolation kit (Takara #630467), transformed into *E. coli* DH5α (Clontech), reisolated, and then sequenced to identify the insert cDNA using BLASTn analysis.

The full‐length *ACAP1* prey plasmid pGADT7‐ACAP1_FL_ was constructed as described above. The resulting plasmid pGADT7‐ACAP1_FL_ was introduced into strain Y187 for direct mating with Y2HGold (pGBKT7‐*bspF*). Production of GAL4‐BD and GAL4‐AD fusions were verified by Western blotting using either anti‐myc or anti‐HA antibodies, respectively.

#### Immunoprecipitation

To examine BspF–ACAP1 interactions, HeLa cells seeded in 10 cm tissue culture dishes (1 × 10^6^ cells/dish) were transfected at 70–80% confluency either with pCMV‐HA (empty vector) and pCMV‐myc‐ACAP1 or pCMV‐HA‐*bspF* and pCMV‐myc‐ACAP1 following the FuGENE® 6 manufacturer’s instructions. To examine ACAP1–Arf6 interaction in the presence or absence of BspF, HeLa cells were transfected either with pCMV‐myc (empty vector), pCMV‐HA (empty vector), and pcDNA3‐Arf6‐HA; pCMV‐myc (empty vector), pCMV‐HA‐*bspF*, and pcDNA3‐Arf6‐HA; pCMV‐myc‐ACAP1, pCMV‐HA (empty vector), and pcDNA3‐Arf6‐HA; or pCMV‐myc‐ACAP1, pCMV‐HA‐*bspF*, and pcDNA3‐Arf6‐HA following the FuGENE^®^ 6 manufacturer’s instructions. After 23.5‐h transfection, the medium was replaced with complete medium containing AlF_4_ (10 mM NaF, 100 μM AlCl_3_) and incubated at 37°C, 5% CO_2_ for 30 min. After 24 h of transfection, cells were washed in cold PBS and proteins were crosslinked with a 0.5mM dithiobis[succinimidylpropionate] (DSP) (Thermo Scientific) in PBS solution at 4°C for 2 h. DSP was quenched with cold 20 mM Tris pH 7.4 PBS for 15 min at 4°C. Cells were washed with cold PBS and lysed in 0.5 ml of lysis buffer (20 mM HEPES, 125 mM NaCl, 1 mM MgCl_2_, 0.5% Triton X‐100 (v/v), and 1:500 HALT protease inhibitor (Thermo Scientific)) on ice for 30 min, and lysates were clarified at 12,000 × *g* for 5 min at 4°C. Protein A Magnetic Beads (Thermo Scientific; 200 µl/6 × 10^6^ cells) were rinsed in PBS and incubated with clarified lysates for 1 h at 4°C to pre‐clear lysates. Anti‐Myc‐conjugated or anti‐HA‐conjugated Dynabeads (Novex, Life Technologies; 400 µl/6 × 10^6^ cells) were rinsed in PBS and lysis buffer, and blocked in 1 ml sterile 2% BSA (Sigma‐Aldrich, A8806) PBS for 1 h at 4°C. Anti‐HA‐conjugated or anti‐myc‐conjugated beads were washed with PBS and incubated with pre‐cleared lysates for 2 h at 4°C, then washed five times in wash buffer (20 mM HEPES, 500 mM NaCl, 1 mM MgCl_2_, 0.5% Triton X‐100 (v/v)). Bound proteins were eluted in 50 μl of 120 mM Tris–HCl pH 6.8, 1% glycerol (v/v), 120 mM SDS, and 0.4% bromophenol blue (w/v), and then heated at 95°C for 5 min. Immunoprecipitated proteins were separated by SDS–PAGE, transferred to nitrocellulose membrane for Western blot analysis, and quantification was performed by densitometric analysis using ImageLab 6.1 (Bio‐Rad).

#### Differential detergent fractionation

HeLa cells were seeded in 6‐well dishes at 2.4 × 10^5^ cells/well, transfected for 17 h, and subjected to differential detergent fractionation as described previously (Knodler *et al*, 2011) with the following modifications. Cells were washed in cold 1× PBS and incubated in 200 μl saponin lysis buffer (50 mM Tris–HCl (pH 7.6), 0.1% (w/v) saponin with protease inhibitors (Calbiochem Complete Mini EDTA‐free)) on ice for 5 min. Cells were scraped and collected, and wells washed with an additional 100 μl saponin lysis buffer for a total of 200 µl. Samples were centrifuged at 3,000 × *g* for 5 min at 4°C. Saponin‐soluble proteins were precipitated with 10% (w/v) trichloroacetic acid, washed in acetone, and solubilized in 120 μl of 1.5× SDS–PAGE sample buffer. Saponin‐insoluble proteins were solubilized in 100 μl Triton X‐100 lysis buffer (50 mM Tris–Cl pH 7.6, 0.5% (v/v) Triton X‐100 with protease inhibitors) and incubated on ice for 15 min, followed by centrifugation at 5,000 × *g* for 10 min at 4°C. Supernatants containing Triton X‐100–soluble proteins were collected and 20 µl 6× SDS–PAGE sample buffer added. Finally, the Triton X‐100–insoluble pellet was solubilized in 120 µl of 1.5× SDS–PAGE sample buffer. Saponin‐, Triton X‐100–, and SDS‐soluble fractions were analyzed by Western blotting.

#### Western blotting

Bacterial and mammalian cell lysates were generated using 2× SDS–PAGE sample buffer (0.12 M Tris (pH 6.8), 10% (v/v) glycerol, 3.4% (w/v) SDS, 0.2 M dithiothreitol [DTT], 0.004% (w/v) bromophenol blue). Samples were boiled at 95°C for 10 min and loaded at equal volumes or according to loading controls. Proteins were resolved on SDS–PAGE and transferred onto 0.45 µm or 0.2 µm nitrocellulose membranes (Amersham Hybond‐ECL, GE Healthcare). Membranes were blocked in TBST (0.14 M NaCl, 0.02 M Tris (pH 7.6), 0.1% (w/v) Tween‐20), 5% (w/v) nonfat dry skim milk powder for 2 h at room temperature and probed with primary antibodies overnight at 4°C, then with HRP‐conjugated secondary antibodies, and all diluted in TBST‐milk. Western blots were developed using the Super Signal West Femto Maximum Sensitivity Substrate (Thermo Scientific) and imaged using a Bio‐Rad ChemiDoc gel imaging system, and representative figures were assembled using Adobe Photoshop CS6.

#### Cholera Toxin B trafficking assay

HeLa cells seeded in a 24‐well plate on glass coverslips (3.5 × 10^4^/well) were transfected at 70–80% confluency with either pmCherry‐C1 (empty vector), pmCherry‐C1‐*bspF*, or pCMV‐HA‐*bspF* following the FuGENE^®^ 6 manufacturer’s instructions. At 24‐h post‐transfection, cells were placed on ice and medium was replaced with ice‐cold complete medium containing Alexa Fluor™ 488‐conjugated recombinant Cholera Toxin Subunit B, (CTxB; 5 μg/ml; Invitrogen, C22841) for 30 min. Cells were washed with serum‐free medium and replaced with 37°C complete medium and incubated at 37°C, 5% CO_2_, to initiate CTxB traffic that was followed over a 30‐min time course. BMMs were seeded in a 24‐well plate on glass coverslips (1 × 10^5^/well). 72 h after seeding, cells were incubated on ice and medium was replaced with ice‐cold complete medium containing Alexa Fluor™ 488‐conjugated CTxB (0.2 μg/ml) for 10 min. Cells were washed with serum‐free medium and replaced with pre‐warmed 37°C complete medium and incubated at 37°C, 10% CO_2_ to initiate CTxB traffic, which was followed for 60 min. Cells were fixed in 3% paraformaldehyde in 1× PBS for 20 min at 37°C, and the Golgi apparatus was counterstained with an anti‐GM130 antibody. Coverslips were mounted blind onto glass slides for analysis via immunofluorescence microscopy. 100 CTxB positive cells that were either transfected or infected were analyzed per experiment. Cells were counted as positive when the CTxB signal colocalized with GM130‐positive Golgi structures at the time point analyzed. Cells in which the CTxB signal remained at the plasma membrane or had not reached the Golgi apparatus at the timepoint of analysis were counted as negative.

#### Syntaxin 6 recruitment assay

BMMs were seeded in 24‐well plates on coverslips (1 × 10^5^/well, or 1 × 10^6^ for siRNA knockdown), infected with DsRed_m_‐expressing bacteria for 24 h, and fixed in 3% paraformaldehyde in 1× PBS for 20 min at 37°C. Cells were permeabilized in 0.5% Triton X‐100 for 5 min at room temperature and stained for Syntaxin 6 (1:100) overnight at 4°C followed by incubation with Alexa Fluor™ 488‐conjugated secondary anti‐rabbit antibodies. Coverslips were mounted blind on glass slides and analyzed via confocal microscopy. Five random fields containing infected BMMs were imaged (single 0.33 µm Z‐sections) per sample for analysis. Analysis of Stx6‐positive vesicle recruitment to rBCVs was performed using the cell image analysis software CellProfiler 4.0.7 (https://cellprofiler.org) according to the following analysis pipeline (Fig EV4). Two 10.5 µm^2^ areas per infected cell were selected blind in the DsRed channel (bacteria), overlayed onto the AlexaFluor™488 channel (Stx6), and input into CellProfiler pipeline. Using the “EnhanceEdges” module, bacterial edges were defined using the Sobel edge finding method. The “Identify Primary Objects” module identified individual bacteria using a size range of 16–20 pixel units. Threshold was measured by an Adaptive strategy and Otsu correction factor, with a smoothing scale of 1.3488 and correction of 1.0, and Shape was used to de‐clump and draw dividing lines between objects. The “ExpandOrShrinkObjects” module expanded each bacterial body by 6 pixels to include rBCV membranes and associated vesicles, based on electron microscopy measurements of rBCVs (Celli *et al*, 2003; Starr *et al*, 2012) Stx6 vesicles were identified using the “IdentifyPrimaryObject” module in a size range of 3–10 pixel units. Identified rBCVs and vesicles were related using the “RelateObjects” module, using bacteria as parent objects and vesicles as children objects. Finally, the “ClassifyObjects” module was used to count the number of vesicles associated with each rBCV defined as expanded bacterium. The percentage of Stx6‐positive rBCVs was then derived from the CellProfiler analysis output, with rBCVs counted as positive when associated with at least one Stx6‐positive structure. At least 300 individual rBCVs per sample were analyzed for their association with Stx6‐positive vesicles, and experiments were repeated independently three times.

#### Arf6 activation assay

To measure levels of active Arf6, HeLa cells were seeded in tissue culture‐treated 6‐well plates (1.5 × 10^5^/well) and transfected for 24 h with either pmCherry‐C1 (empty vector) and pcDNA3‐*arf6‐HA*, or pmCherry‐C1‐*bspF* and pcDNA3‐*arf6‐HA* following the FuGENE® 6 manufacturer’s instructions. Cells were starved for 20 h prior to lysis with FBS‐free medium, then incubated in FBS‐free medium supplemented with 200 ng/ml human epidermal growth factor (EGF, EMD Millipore) at 37°C for 7 min to trigger Arf6 activation. Activation was stopped by placing cells on ice, which were immediately lysed and processed using a G‐LISA Arf6 Activation Kit Assay (Cytoskeleton Inc, BK133) according to the manufacturers’ instructions. Lysates (0.5mg/ml total protein) were added to a 96‐well plate coated with a proprietary GTP‐Arf6‐binding protein and wells were washed 3 times. Bound, active GTP‐Arf6 was detected using an Arf6‐specific antibody followed by HRP detection. Experiments were performed in duplicates and repeated 3 times independently.

#### Data quantification

Statistical analysis was performed using GraphPad Prism 8 software. All data are presented as the means ± standard deviations (SD) of results from at least three independent experiments. Statistical significance of comparisons between control and treatment groups was determined using either an unpaired, two‐tailed Student’s *t*‐test, a Mann–Whitney test, or for group analysis, one‐way or two‐way analysis of variance (ANOVA) followed by either Sidak’s, Dunnett’s, or Tukey’s multiple‐comparison test, based on the experimental design. A *P* < 0.05 was considered significant. The specific statistical tests used are indicated in the corresponding figure legends.

## Author contributions

Conceptualization: EPS and JC; Methodology: EPS, EB, LAK. SM, KB, and JC; Investigation: EPS, EB, SM, LAK, and JC; Writing—original draft: EPS, EB, and JC; Writing—review and editing: EPS, EB, SM, LAK, and JC; Funding acquisition: JC; Resources: LAK and JC; Supervision: JC.

## Conflict of interest

The authors declare that the research was conducted in the absence of any commercial or financial relationships that could be construed as a potential conflict of interest.

## Supporting information



Expanded View Figures PDF

Source Data for Figure 2

Source Data for Figure 3

Source Data for Figure 4

Source Data for Figure 5

Source Data for Figure 6

## Data Availability

This study includes no data deposited in external repositories.
