# Supplementary material for: A Brucella effector modulates the Arf6‐Rab8a GTPase cascade to promote intravacuolar replication
Source: EMBO J. 2021 Aug 23;40(19):e107664. doi: 10.15252/embj.2021107664 (PMC8488576; doi:10.15252/embj.2021107664)
Supplement: Supplementary file 1 — Expanded View Figures PDF [file EMBJ-40-e107664-s006.pdf]

Expanded View Figures

Figure EV1. BspF localizes to the endosomal recycling compartment.

A Representative confocal fluorescence micrograph of HeLa cells co-transfected for 24 h to produce mCherry-BspF and GFP-BspF and treated with Cytochalasin D (200 nM) for 30 min prior to fixation. Scale bars: 10 and 2 μm (insets).

B Representative confocal fluorescence micrographs of HeLa cells co-transfected for 24 h to produce mCherry-BspF and either GFP-MICAL-L1, GFP-STX16, GFP-STX6, or GFP-VAMP3 and treated with Cytochalasin D (200 nM) for 30 min prior to fixation. Scale bars: 10 and 2 μm (insets). Localization of GFP-MICAL-L1, GFP-STX16, GFP-STX6, or GFP-VAMP3 to mCherry-BspF-labeled tubules was quantified in at least 300 individual cells per experiment. Data are means ± SD from n = 3 independent experiments.

C Representative confocal fluorescence micrographs of HeLa cells co-transfected for 24 h to produce mCherry-BspF and either GFP-Rab11a or VAMP4-GFP and treated with Cytochalasin D (200 nM) for 30 min prior to fixation. Scale bars: 10 and 2 μm (insets). Localization of GFP-Rab11a or VAMP4-GFP to mCherry-BspF-labeled tubules was quantified in at least 300 individual cells per experiment. Data are means ± SD from n = 3 independent experiments.

 © 2021 The Authors

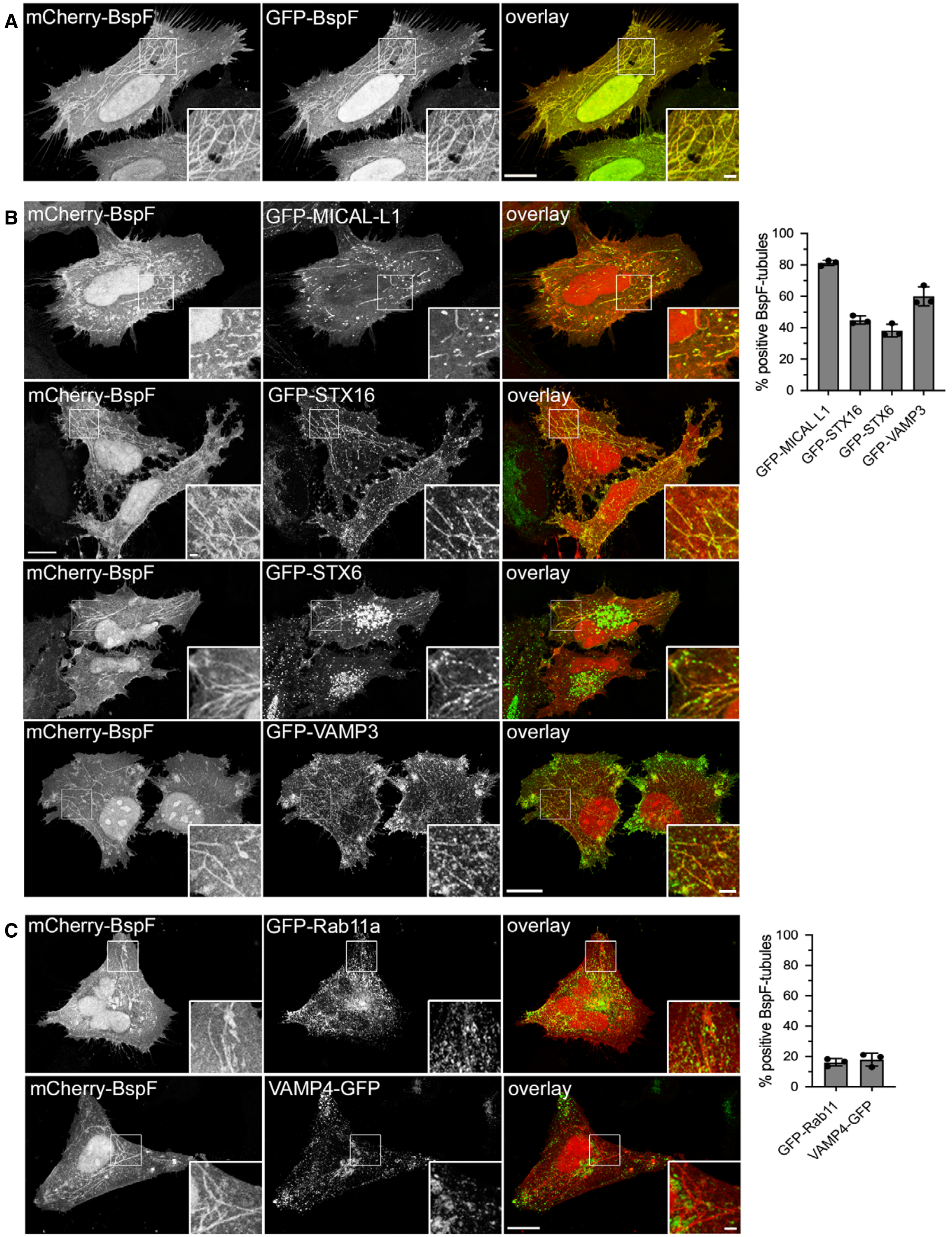
A
mCherry-BspF
GFP-BspF
overlay
B
mCherry-BspF
GFP-MICAL-L1
overlay
mCherry-BspF
GFP-STX16
overlay
mCherry-BspF
GFP-STX6
overlay
mCherry-BspF
GFP-VAMP3
overlay
% positive BspF-tubules
100
80
60
40
20
0
GFP-MICAL L1
GFP-STX16
GFP-STX6
GFP-VAMP3
C
mCherry-BspF
GFP-Rab11a
overlay
mCherry-BspF
VAMP4-GFP
overlay
% positive BspF-tubules
GFP-Rab11
VAMP4-GFP

Figure EV1.

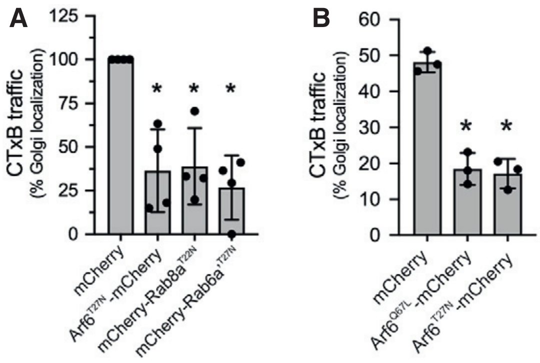

Figure EV2. Retrograde transport of Cholera toxin depends upon Arf6, Rab8a, and Rab6a′ in HeLa cells and BMMs.

A, B Quantification of CTxB transport to the Golgi apparatus in either HeLa cells producing either mCherry, Arf6^{T27N}-mCherry, mCherry-Rab8a^{T22N}, or mCherry-Rab6a$'^{T27N}$ (A), or in BMMs producing either mCherry, Arf6^{Q67L}-mCherry, or Arf6^{T27N}-mCherry (B). Cells were transfected for 24 h (A) or transduced for 48 h (B) then incubated on ice with AlexaFluor488™-Cholera Toxin subunit B (CTxB) for binding followed by a 20-min (A) or 30-min (B) incubation at 37°C to allow for CTxB retrograde transport to the Golgi apparatus (stained using an anti-GM130 antibody). CTxB retrograde transport is expressed as percentages of cells in which CTxB colocalized with the GM130 Golgi marker. Data are means ± SD from n = 3 to 4 independent experiments, in which 100 cells were analyzed per experiment. Asterisks indicate statistically significant differences compared with mCherry-producing cells as determined by a one-way ANOVA with Dunnett's multiple comparisons test ($P < 0.05$).

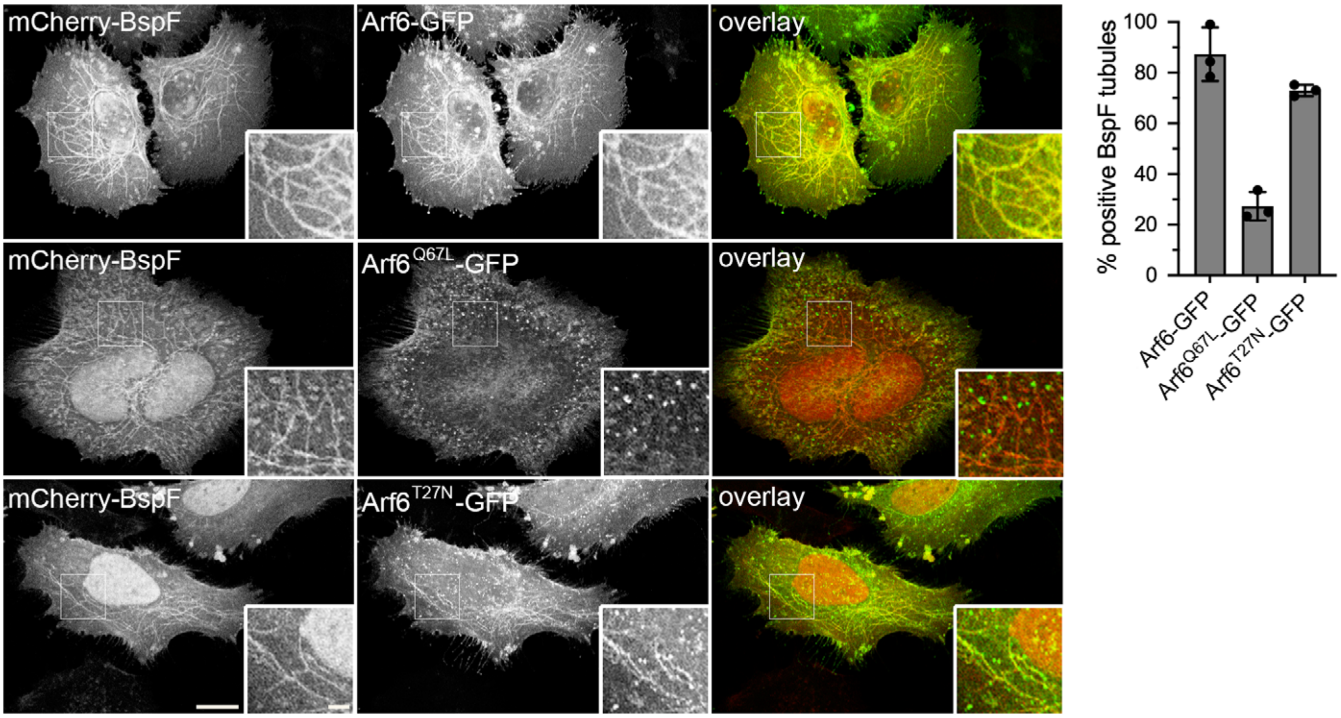

Figure EV3. Localization of Arf6-GFP alleles to mCherry-BspF-labeled tubules.

Representative confocal fluorescence micrographs of HeLa cells co-transfected for 24 h to produce mCherry-BspF and either Arf6-GFP, Arf6^{Q67L}-GFP, or Arf6^{T27N}-GFP and treated with Cytochalasin D (200 nM) for 30 min prior to fixation. Scale bars: 10 and 2 μm (insets). Localization of Arf6-GFP, Arf6^{Q67L}-GFP, or Arf6^{T27N}-GFP to mCherry-BspF-labeled tubules was quantified in at least 300 individual cells per experiment. Data are means ± SD from n = 3 independent experiments.

 © 2021 The Authors

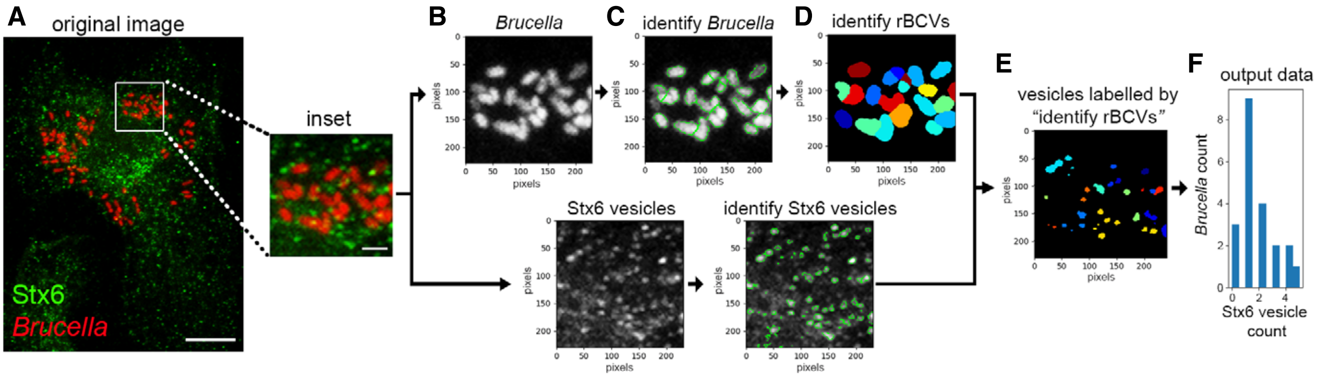

Figure EV4. CellProfiler analysis pipeline of Stx6-positive vesicle recruitment to rBCVs.

A A confocal micrograph inset (10.5 μm^2 area) from a BMM infected with wild-type $DsRed_m$-expressing *B. abortus* and stained for endogenous Syntaxin 6 (AlexaFluor™488-Stx6) was selected in the DsRed channel (*Brucella*) and input into CellProfiler for analysis. Scale bars, 10 and 2 μm.

B *Color to gray* module split the red (*Brucella*) and green (Stx6) channels and reverted the images to gray scale.

C *Identify primary objects* module identified individual *Brucella* and Stx6-positive vesicles based on their size.

D *Expand or shrink objects* module expanded the size of individual *Brucella* by 6 pixels to encompass whole rBCVs and associated vesicles.

E *Relate objects* module identified Stx6-positive vesicles within the rBCV area and filtered out non-associated vesicles.

F *Classify objects* module counted the number of vesicles associated with each rBCV (expanded *Brucella*). X- and y-axes represent pixels coordinates. The output data counted the number of vesicles associated with each *Brucella*, which was derived to determine the percentage of Stx6-positive rBCVs.

© 2021 The Authors
