# Supplementary figures and images for: A Brucella effector modulates the Arf6‐Rab8a GTPase cascade to promote intravacuolar replication
Source: EMBO J. 2021 Aug 23;40(19):e107664. doi: 10.15252/embj.2021107664 (PMC8488576; doi:10.15252/embj.2021107664)

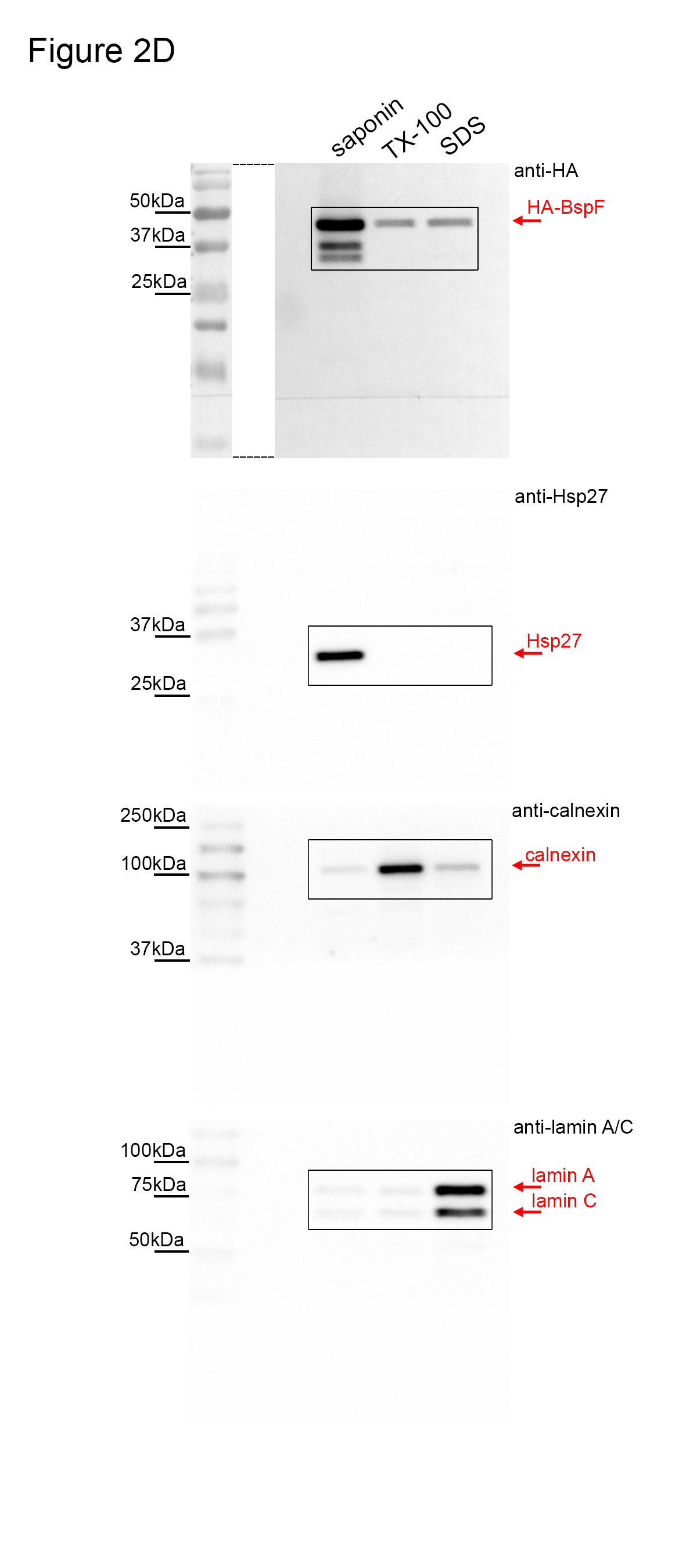

Supplement: Supplementary file 2 — Source Data for Figure 2 [file EMBJ-40-e107664-s007.jpg]

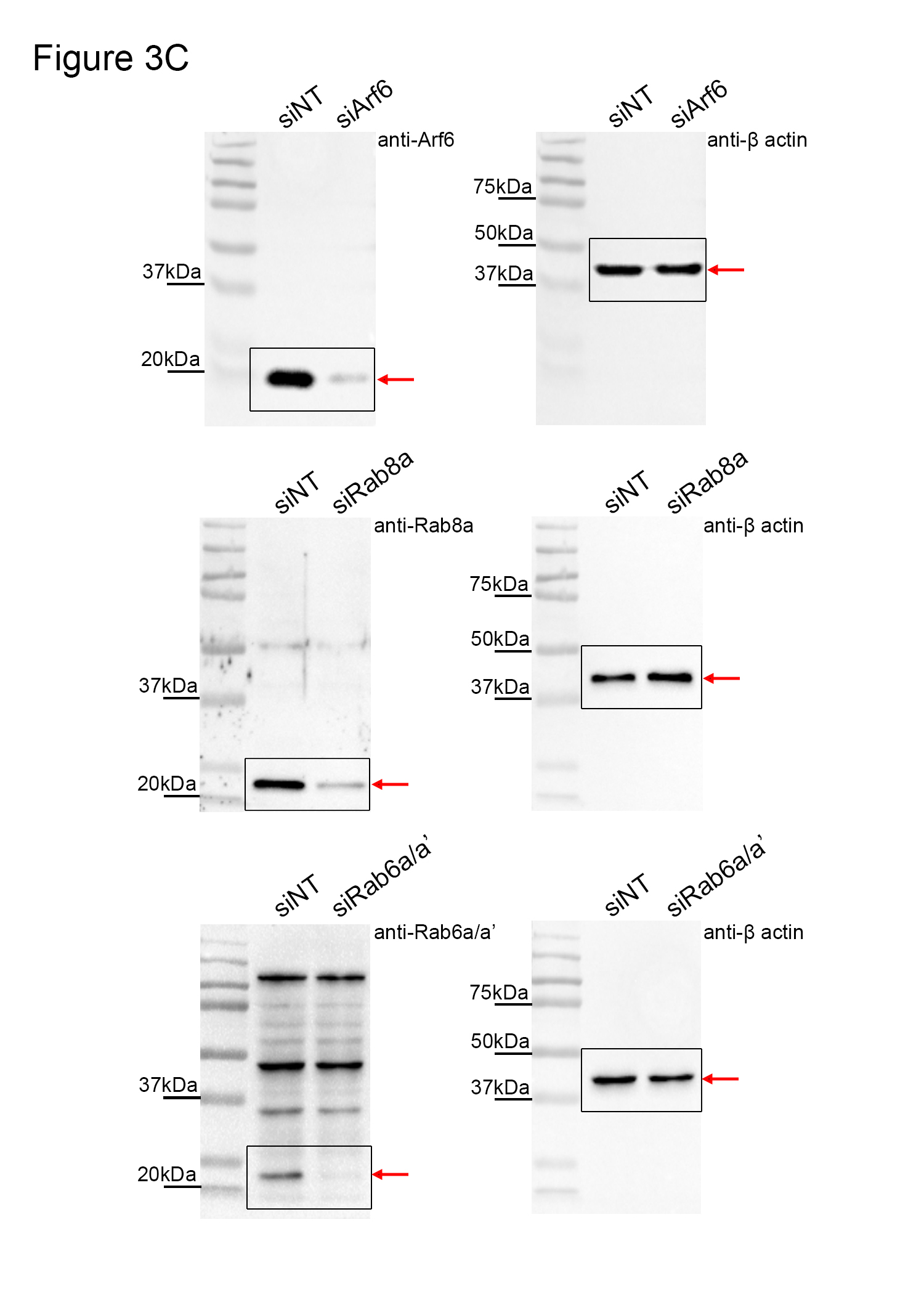

Supplement: Supplementary file 3 — Source Data for Figure 3 [file EMBJ-40-e107664-s002.jpg]

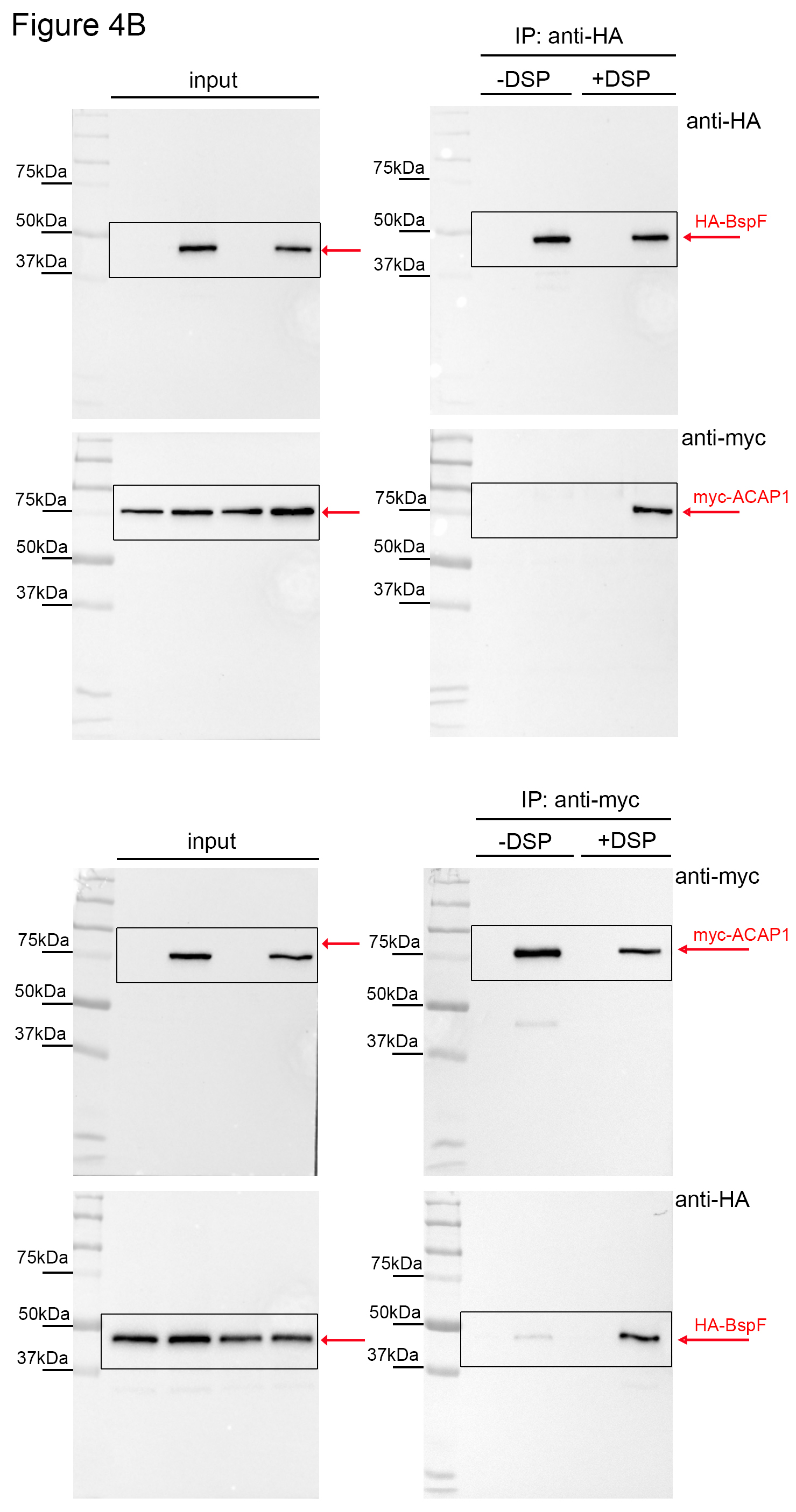

Supplement: Supplementary file 4 — Source Data for Figure 4 [file EMBJ-40-e107664-s001.jpg]

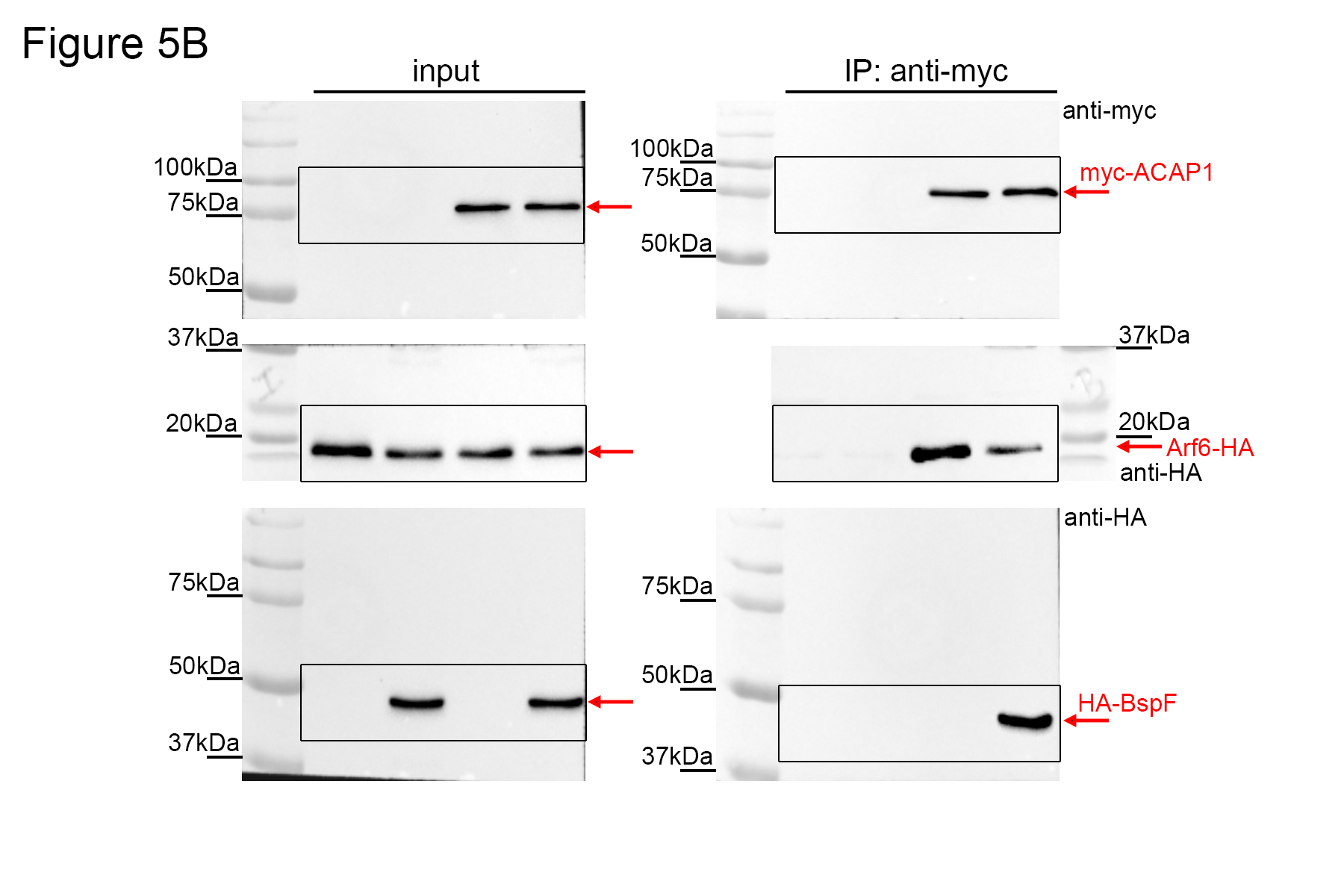

Supplement: Supplementary file 5 — Source Data for Figure 5 [file EMBJ-40-e107664-s005.jpg]

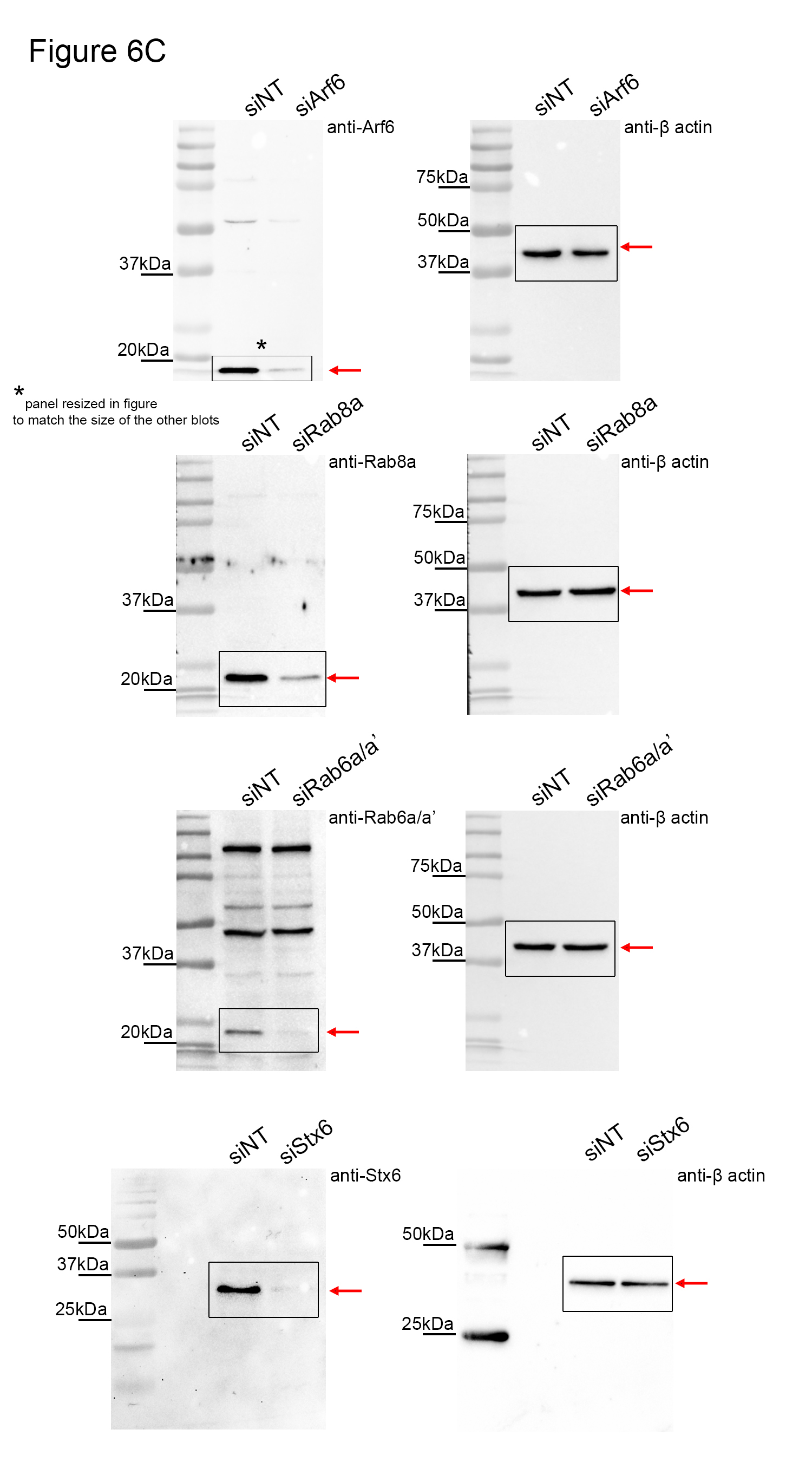

Supplement: Supplementary file 6 — Source Data for Figure 6 [file EMBJ-40-e107664-s003.jpg]
