# Supplementary material for: A Brucella effector modulates the Arf6‐Rab8a GTPase cascade to promote intravacuolar replication
Source: EMBO J. 2021 Aug 23;40(19):e107664. doi: 10.15252/embj.2021107664 (PMC8488576; doi:10.15252/embj.2021107664)
Supplement: Supplementary file 7 [file EMBJ-40-e107664-s004.pdf]

THE EMBO JOURNAL

A Brucella effector modulates the Arf6-Rab8a GTPase cascade to promote intravacuolar replication

Jean Celli, Elizabeth Borghesan, Erin Smith, Sebenzile Myeni, Kelsey Binder, and Leigh Knodler
DOI: 10.15252/embj.2021107664

Corresponding author(s): Jean Celli (jean.celli@wsu.edu)

Review Timeline:

Submission Date:	6th Jan 21
Editorial Decision:	11th Feb 21
Revision Received:	9th Jun 21
Editorial Decision:	12th Jul 21
Revision Received:	26th Jul 21
Accepted:	30th Jul 21

Editor: Elisabetta Argenzio

Transaction Report:

(Note: With the exception of the correction of typographical or spelling errors that could be a source of ambiguity, letters and reports are not edited. Depending on transfer agreements, referee reports obtained elsewhere may or may not be included in this compilation. Referee reports are anonymous unless the Referee chooses to sign their reports.)

1st Editorial Decision 11th Feb 2021

Thank you for submitting your manuscript entitled "Remodeling of trans-Golgi network transport by a Brucella effector promotes intravacuolar replication" [EMBOJ-2020-107664] to The EMBO Journal. Your study has now been assessed by three reviewers, whose reports are enclosed below for your information.

As you can see, referee #1 finds the study novel and interesting and supports publication upon satisfactorily addressing a few minor points. However, referee #2 points out that the mechanism of action of BspF is not sufficiently investigated, whereas referee #3 stresses that conclusions are not supported by data.

Given the overall interest of your study, we have decided to invite you to submit a new version of the manuscript revised according to the referees' requests. I should add that it is The EMBO Journal policy to allow only a single round of revision, and acceptance of your manuscript will therefore depend on the completeness of your responses in the revised version. Please note that strong support from the referees would also be needed for publication here.

We generally grant three months as standard revision time. As we are aware that many laboratories cannot function at full capacity owing to the COVID-19 pandemic, we may relax this deadline. Also, we have decided to apply our 'scooping protection policy' to the time span required for you to fully revise your manuscript and address the experimental issues highlighted herein. Nevertheless, please inform us as soon as a paper with related content is published elsewhere.

When preparing your letter of response to the referees' comments, please bear in mind that this will form part of the Review Process File and will therefore be made available online. For more details on our Transparent Editorial Process, please visit our website: http://emboj.embopress.org/about#Transparent_Process

Before submitting your revised manuscript, deposit any primary datasets and computer code produced in this study in an appropriate public database (see http://msb.embopress.org/authorguide#dataavailability). Please remember to provide a reviewer password, in case such datasets are not yet public. The accession numbers and database names should be listed in a formal "Data Availability" section (placed after Materials & Method). Provide a "Data availability" section even if there are no primary datasets produced in the study.

Feel free to contact me if you have any questions about the submission of the revised manuscript to The EMBO Journal. I thank you again for the opportunity to consider this work for publication and look forward to your revision.

Referee #1:

The study by Smith et al. investigates the mode of action of the type IV-translocated effector protein BspF produced by Brucella abortus, a zoonotic pathogen that causes brucellosis, which replicates intracellularly in an ER-derived, replication-permissive vacuole (rBCV). Specifically, the authors demonstrate that ectopically produced BspF (i) targets a tubular membrane compartment involved in the transport between the trans-Golgi network (TGN) and recycling endosomes (RE), (ii)

interferes with retrograde trafficking of fluorescently labeled Cholera toxin subunit B (CTxB), (iii) co-localizes with the small GTPases Rab8a (RE) and Arf6, but not with Rab6a (TGN), and (iv) abrogates the accumulation of the Arf6 GTPase-activating protein (GAP) ACAP1 on tubular RE. A yeast two-hybrid screen and co-immunoprecipitation assays revealed that BspF interacts with the C-terminal part of ACAP1 (harboring the GAP domain and ankyrin repeats). Moreover, ectopically produced BspF inhibited the interaction between Arf6 and ACAP1, while reducing the amount of activated Arf6-GTP.
Upon infection of bone marrow-derived macrophages (BMM) with B. abortus wild-type, a bspF mutant strain or the genetically complemented mutant, (i) the bspF mutant was impaired for replication and no longer inhibited retrograde trafficking of CTxB, and (ii) the replication phenotype of the bspF mutant was reverted upon overexpression of ACAP1. Furthermore, the depletion by RNA interference of Rab6a/a', Rab8 or Arf6 in BMM had opposite effects on (iii) the intracellular replication of B. abortus wild-type (decrease) and the bspF mutant (increase), or (iv) the accumulation on rBCVs of the SNARE syntaxin 6 (Stx6), a marker of TGN-derived vesicles. Finally, (v) the depletion of Stx6 impaired intracellular replication of B. abortus wild-type.

In summary, the manuscript convincingly reveals a novel and intriguing mechanism of the B. abortus effector BspF. The RE-localizing effector binds to the Arf6 GAP ACAP1, resulting in decreased Arf6-ACAP1 interactions, reduced Arf6-GTP and increased recruitment of TGN-derived, Stx6-positive vesicles to rBCVs. The well-controlled study includes state-of-the art fluorescence microscopy as well as biochemical interaction tests and infection assays, and the story unfolds in a straightforward manner. Some minor issues should be addressed to further strengthen the work.

Specific points
1) Consider a more specific title for the manuscript.

2) Fig. EV1A and EV1B should be switched, as EV1B is currently called out in the text before EV1A (l. 136).

3) Consider relegating the Western blots showing RNAi depletion efficiency to the EV section (Figs. 3CFH, 6C). This would further enhance the flow of the story.

4) Fig. 5A: A control for mCherry is missing. However, (abundant cytosolic) mCherry perhaps confounds the interpretation of the results?

5) Fig. 6A: Fluorescence images for the complemented strain could be shown.

6) Discussion:
- l. 346: L. pneumophila has also been shown to capture Golgi-derived vesicles (PMID: 30538188). This could be discussed here.
- l. 355-359: The structure of the L. pneumophila effector RidL (in complex with Vps29) has been (partially) solved (PMID: 29146912, PMID: 29229824, PMID: 29386389). These references could be discussed here.

7) Figure legends
- l. 884/953/994/1024: the number of cells counted could be indicated (n = ?).
- l. 983: ... performed by densitometric analysis (lower panel).

8) Wording/typos
- l. 97: define "BMM" the first time the abbreviation appears in the text.

- l. 126: ... identify the BspF-targeted ...
- l. 167: ... and CTxB traffic was analyzed ...
- l. 275/276: "Fig. 6H" (not "Fig. 6G").
- l. 708-710: reference incomplete.
- l. 790-791: reference incomplete.
- l. 822: ... Bärlocher K, ...
- l. 924/940/947/1010: "??-actin".

Referee #2:

In this study, the authors report that replication of the intracellular bacterial pathogen Brucella abortus is promoted by its effector BspF, which interferes with the host's vesicular transport between the trans-Golgi network (TGN) and recycling endocytic compartment. Although the authors have previously implicated BspF in bacterial proliferation, understanding the mode of host manipulation by this effector protein brings substantial novelty to the table.

Overall, the data is of high quality and appears reproducible. The evidence provided largely supports the conclusions of the authors, with one important--but not disqualifying--exception (see major points). The central weakness of the study in my opinion is the limited degree to which the mechanism of action of BspF is developed, particularly for a journal such as EMBO. While the authors convincingly demonstrate that the Brucella effector BspF targets the Arf6/Rab8a trafficking route, the mode of BspF action in this context remains debatable, as detailed below. Given the quality of the work presented thus far, I am in principle supportive of the study's publication--if (and only if) the authors can make substantial inroads on the molecular mechanism during the revision process.

Major points:

The data on the interaction between BspF and ACAP1 is solid, however, the interpretation of its consequences by the authors--namely that BspF inactivates Arf6--are in my opinion run counter to expectations and are not supported by several strong lines of evidence provided in the manuscript. My main arguments against the interpretation are as follows.

#1 In fig. 5B, the authors provide compelling evidence that BspF inhibits the interaction between ACAP1 and Arf6. ACAP1 is an established GAP for Arf6, and GAPs by their nature are facilitators of the GTP hydrolysis cycle, thus rendering their cognate GTPase inactivate. I would therefore expect that diminished encounters with its GAP would result in prolonged activation of Arf6, not diminished activation, as claimed here. This expectation is further supported by the accumulation of Arf6 on tubules and enlarged endosomes. Typically (although granted not always), small GTPases driving the endocytic network are relegated to the cytosol in their inactive state, while constitutively active mutants tend to accumulate on distended and/or swollen membranes. If the authors claim that in this situation BspF causes inactivation of Arf6, additional evidence besides the GTP loading presented in Fig. 5C is required to substantiate the issue. Furthermore, the selection of zoom-in area in the right panels of Fig. 5A ignores ample areas of white overlays, indicating colocalization between all 3 components, ACAP1/Arf6/BspF.

#2 The yeast-2-hybrid data presented in Fig. 4A suggests that ACAP1 binds BspF with the same region containing the GAP activity. Taken together with diminished interaction between ACAP1 and Arf6 in the presence of BspF, it is tempting to consider whether BspF sequesters ACAP1 from its substrate Arf6. This would once again support the notion that in the presence of BspF, Arf6 would be retained in its active state. In my opinion the authors need to provide more evidence to reconcile this with the data in Fig. 5C.

It may very well be that by virtue of such hyperactivation of Arf6 (rather than its hypoactivation as argued by the authors) the transport route in question is disrupted, because vesicular transport requires its regulatory GTPases to remain dynamic (i.e. transitioning between active and inactive states).

#3 Co-suppression/reconstitution data in Fig. 3K is not convincing and does not help the author's case regarding the proposed BspF-induced inactivation of Arf6.

Minor points:

#1. It may be better to combine Fig. 1 with Fig. 2A for a more substantial opening argument.

#2. data in Fig. 2b needs to be quantified to report colocalization. Additionally, attachment of tubular structures to vesicles (as described in the text) is highly speculative.

#3. Although the images shown in Fig. 2e are very convincing, quantification in the form of some overlap coefficient derived from multiple cells and at least 2 independent experiments should be provided.

#4. In my opinion data shown in Figures 4 and 5 should be presented in one figure to improve clarity and flow. Redundant data can be moved to the supplementary to alleviate space constraints.

#5. Data on the acquisition of STX6 by the bacterial phagosome are not very convincing. The differences in the images shown are not striking and the quantification is based on manual counting rather than less biased colocalization coefficients. The overall study would benefit from further solidifying this line of observations.

Referee #3:

In the current manuscript the authors investigate the role of BspF, which has previously been demonstrated to be secreted by Brucella via the type IV secretion system. Previous data from ectopic expression experiments demonstrated that BspF interferes with the host secretory pathway. Here they generated a BspF mutant which is impaired in replication in macrophages. Ectopic expression of BspF demonstrated the targeting of the recycling endosome. They identified in a Yeast Two hybrid screen Arf6 GTPase-activating Protein ACAP1 which interferes with the vesicular transport within the recycling endosome and supports the replication of Brucella in rBCVs.

The subversion of host cell vesicular trafficking by bacterial pathogens is a current topic in the field. The current study would contribute to the mechanistic understanding of these events, however, the results presented and the design of the experiments in the present manuscript do not justify the conclusions drawn by the authors.

Major points

1. The major problem with this study is that there is no evidence provided that BspF secreted by Brucella really targets the recycling endosome, interacts with ACAP1 and functions by the interference with Arf6/Rab8a-dependent transport. It is definitely easier to use ectopic expression constructs but these overexpression experiments are prone to generate artifacts and at least need to be validated by demonstrating localization of BspF in selected infection experiments (Fig. 2). This is particularly important with ectopically expressed bacterial proteins that are supposed to target intracellular trafficking since they are produced and transported within the compartments they have to target from outside in natural infections.

2. Figure 2B and 2C require quantification from different fields and different cells.

3. In figure 3E, CtxB trafficking is demonstrated to be affected as consequence of wildtype infection but not of the bspF mutant. Since the bspF mutant has a growth defect (Fig. 1B) the question is whether the bspB mutant which is used as a control in this experiment has the same growth defect. If not, the control is hardly adequate and should be replaced.

4. Experiments in figure 4B need additional controls. For IP experiments an unrelated Bsp protein should be used as negative control. And even more importantly, native complexes should be demonstrated in IPs by pulling down endogenous BspF and ACAP1 from infected cells. The high efficiency of co-IP does not fit at all to the picture of co-localization provided in figure 4C.

5. Overexpression of GFP-ACAP1 in figure 4 C causes aggregates in the cells to which the mCherry-BspF co-localizes. Since these are the only structures to which co-localization occurs the authors should try to titrate the GFP-ACAP1 to avoid aggregates. Otherwise the co-localization of BspF and ACAP1 occurs in a such a minor compartment compared to the overall expression pattern, that a functional impact of this interaction can be questioned. This interaction in the cell should be quantified in any case.

Minor:

Line 82: What is "aBCV"?

Line 97: Explain BMM.

Massive aggregation of STX6: This image should be exchanged for an image that shows less aggregates.

EMBOJ-2021-107664 - Responses to referees' comments

Referee #1:

In summary, the manuscript convincingly reveals a novel and intriguing mechanism of the B. abortus effector BspF. The RE-localizing effector binds to the Arf6 GAP ACAP1, resulting in decreased Arf6-ACAP1 interactions, reduced Arf6-GTP and increased recruitment of TGN-derived, Stx6-positive vesicles to rBCVs. The well-controlled study includes state-of-the art fluorescence microscopy as well as biochemical interaction tests and infection assays, and the story unfolds in a straightforward manner. Some minor issues should be addressed to further strengthen the work.

> We thank this referee for their enthusiastic assessment of our manuscript. As detailed below, and in response to the other referees' comments, we have addressed the concerns raised and present a revised, improved version of the manuscript.

Specific points
1) Consider a more specific title for the manuscript.

> We propose a more specific title that refers to the targeting of the Arf6-Rab8 cascade by BspF.

2) Fig. EV1A and EV1B should be switched, as EV1B is currently called out in the text before EV1A (l. 136).

> Instead of switching the panels on the figure, we have now revised the order of the text (lines 145-149 of the revised manuscript) to cite data from new Fig. EV1A prior to that shown in new Fig.EV1B.

3) Consider relegating the Western blots showing RNAi depletion efficiency to the EV section (Figs. 3CFH, 6C). This would further enhance the flow of the story.

> To streamline these figures, we have removed redundant siRNA depletion Western blots from Fig. 3 and now only show a set of representative blots in both Fig. 3C and 6C. We also cite in the text depletion levels that correspond to specific experiments.

4) Fig. 5A: A control for mCherry is missing. However, (abundant cytosolic) mCherry perhaps confounds the interpretation of the results?

> We thank the referee for the suggestion. We now present new representative micrographs in Fig. 5A, including an mCherry control that shows colocalization of GFP-ACAP1 and Arf6-HA to tubular structures. Of note, tubular structures generated by Cytochalasin D treatment in mCherry control cells (i.e. not expressing BspF) were shorter and more peripheral than the BspF-labelled tubules, yet they clearly document colocalization of ACAP1 and Arf6 on tubular structures.

5) Fig. 6A: Fluorescence images for the complemented strain could be shown.

> We thank the referee for this valid suggestion. We have now added to Fig. 6A a representative micrograph showing restoration on Stx6-positive vesicle recruitment to rBCVs containing the complemented Δ*bspF* mutant.

6) Discussion:
- l. 346: L. pneumophila has also been shown to capture Golgi-derived vesicles (PMID: 30538188). This could be discussed here.
- l. 355-359: The structure of the L. pneumophila effector RidL (in complex with Vps29) has been (partially) solved (PMID: 29146912, PMID: 29229824, PMID: 29386389). These references could be discussed here.

> We thank the referee for their suggestion and now discuss these studies in the context of acquisition of TGN-derived membranes by bacterial vacuoles (lines 354-356 of the revised manuscript) and the mode of action of RidL on retrograde transport (lines 372-376 of the revised manuscript).

7) Figure legends
- l. 884/953/994/1024: the number of cells counted could be indicated (n = ?).
- l. 983: ... performed by densitometric analysis (lower panel).

> We have included the number of cells, BCVs or bacteria counted in the corresponding figure legends. The incomplete sentence has now been corrected.

8) Wording/typos
- l. 97: define "BMM" the first time the abbreviation appears in the text.
- l. 126: ... identify the BspF-targeted ...
- l. 167: ... and CTxB traffic was analyzed ...
- l. 275/276: "Fig. 6H" (not "Fig. 6G").
- l. 708-710: reference incomplete.
- l. 790-791: reference incomplete.
- l. 822: ... Bärlocher K, ...
- l. 924/940/947/1010: "??-actin".

> All these errors and omissions have now been corrected.

Referee #2:

Overall, the data is of high quality and appears reproducible. The evidence provided largely supports the conclusions of the authors, with one important--but not disqualifying--exception (see major points). The central weakness of the study in my opinion is the limited degree to which the mechanism of action of BspF is developed, particularly for a journal such as EMBO. While the authors convincingly demonstrate that the Brucella effector BspF targets the Arf6/Rab8a trafficking route, the mode of BspF action in this context remains debatable, as detailed below. Given the quality of the work presented thus far, I am in principle supportive of the study's publication--if (and only if) the authors can make substantial inroads on the molecular mechanism during the revision process.

> We thank this referee for their positive evaluation of our manuscript and acknowledge their concerns about the mechanism of action of BspF. As detailed below in our responses to their specific points, we now provide new experimental evidence that supports our model of the mode of action of BspF on Arf6 activity.

Major points:

The data on the interaction between BspF and ACAP1 is solid, however, the interpretation of its consequences by the authors--namely that BspF inactivates Arf6--are in my opinion run counter to expectations and are not supported by several strong lines of evidence provided in the manuscript. My main arguments against the interpretation are as follows.

#1 In fig. 5B, the authors provide compelling evidence that BspF inhibits the interaction between ACAP1 and Arf6. ACAP1 is an established GAP for Arf6, and GAPs by their nature are facilitators of the GTP hydrolysis cycle, thus rendering their cognate GTPase inactivate. I would therefore expect that diminished encounters with its GAP would result in prolonged activation of Arf6, not diminished activation, as claimed here. This expectation is further supported by the accumulation of Arf6 on tubules and enlarged endosomes. Typically (although granted not always), small GTPases driving the endocytic network are relegated to the cytosol in their inactive state, while constitutively active mutants tend to accumulate on distended and/or swollen membranes. If the authors claim that in this situation BspF causes inactivation of Arf6, additional evidence besides the GTP loading presented in Fig. 5C is required to substantiate the issue. Furthermore, the selection of zoom-in area in the right panels of Fig. 5A ignores ample areas of white overlays, indicating colocalization between all 3 components, ACAP1/Arf6/BspF.

> We agree, in principle, with this referee's expectation that the reduced interaction of a GAP protein with its GTPase may lead to increased active GTP-bound form of the latter, and we acknowledged this possibility in our original manuscript. However, this scenario is inconsistent with the following lines of evidence we presented:

- our direct measurement of total active Arf6 shows a significant decrease in BspF-expressing cells, in agreement with Arf6 inactivation;
- ACAP1 overexpression rescues the replication defect of a Δ*bspF* mutant (original Fig. 5D and revised Fig. 5E), arguing that increased ACAP1 function, *i.e.* an enhanced inactivation of Arf6, mimics BspF's role in bacterial replication;
- depletion of Arf6 also rescued the replication defect of a Δb*spF* mutant (original Fig. 3K and revised Fig. 3I), indicating that lacking Arf6 function mimics BspF activity.

Our data supports an alternative possibility that increased GAP activity enhances GTP hydrolysis and dissociation of a GAP protein from its GTPase, which may be reflected by the decreased steady-state interactions we observed. Hence, decreased interactions between these proteins in the presence of BspF is not inconsistent with the model we have proposed.

We also agree that endosomal GTPases tend to localize to membranes in their active form and be cytosolic when inactive. However, Arf6 displays a non-canonical behavior in this respect, in that its inactive GDP-bound form associates with recycling endosomal membranes (Hattula, 2006), unlike most other endosomal GTPases, while active GTP-bound Arf6 tends to associate with the plasma membrane and early endosomal membranes. To document this Arf6 behavior in the context of BspF's effect and address this specific concern, we now provide evidence (new Fig. EV3) that the dominant negative allele $Arf6^{T27N}$ preferentially accumulates on BspF-labelled endosomal tubules over the dominant active allele $Arf6^{Q67L}$. These results therefore argue that inactive Arf6 is the GTPase form that associates with membranes targeted by BspF.

#2 The yeast-2-hybrid data presented in Fig. 4A suggests that ACAP1 binds BspF with the same region containing the GAP activity. Taken together with diminished interaction between ACAP1 and Arf6 in the presence of BspF, it is tempting to consider whether BspF sequesters ACAP1 from its substrate Arf6. This would once again support the notion that in the presence of BspF, Arf6 would be retained in its active state. In my opinion the authors need to provide more evidence to reconcile this with the data in Fig. 5C.

> This referee is correct in that BspF interacts with a C-terminal region of ACAP1 that includes a portion of the GAP domain, suggesting that BspF could interfere with ACAP1 GAP activity. The interacting fragment, however, also contains the full ankyrin (Ank)-repeat domain of ACAP1, which could be the actual region of BspF binding, with the partial GAP domain being a bystander portion of the protein in the ACAP1 fragment hit in our Y2H screen. We unfortunately could not further define which ACAP1 domain interacts with BspF, as domain truncations of ACAP1 were highly unstable in both Y2H and mammalian expression systems. Hence, it remains possible that BspF binds ACAP1 through its Ank domain to gain access to, and modulate the activity of, the adjacent GAP domain, but does not interfere with ACAP1's GAP activity.

It may very well be that by virtue of such hyperactivation of Arf6 (rather than its hypoactivation as argued by the authors) the transport route in question is disrupted, because vesicular transport requires its regulatory GTPases to remain dynamic (i.e. transitioning between active and inactive states).

> We agree with this referee that hyperactivation of Arf6 would also disrupt Arf6-dependent retrograde transport and we provide new evidence that both active and inactive alleles similarly affect Cholera toxin traffic in BMMs (new Fig. EV2B and lines 246-248 of the revised manuscript). Would BspF cause Arf6 hyperactivation, however, we would observe increased total GTP-bound, active Arf6 upon BspF expression, and not the decreased amount measured in Fig. 3C.

To further discriminate between our proposed model and the alternate interpretation of our data proposed by this referee, we have tested the effect of overexpression of dominant Arf6 alleles on the replication of the Δ*bspF* mutant. We reasoned that the dominant Arf6 allele (constitutively active or dominant inactive) that mimics the effect of BspF on Arf6 should rescue bacterial replication and reveal whether BspF activates or inactivates Arf6. Our new data (Fig. 5D of the revised manuscript) shows that the dominant negative allele $Arf6^{T27N}$ but not the constitutively active allele $Arf6^{Q67L}$ rescues replication of the Δ*bspF* mutant, further supporting our model that BspF causes inactivation of Arf6 to promote bacterial replication. This is described in lines 240-245 of the revised manuscript.

Additionally, we have expanded upon our original data showing that ACAP1 overexpression also rescues replication of the Δ*bspF* mutant, by repeating these experiments and including the $ACAP1^{R448Q}$ GAP mutant (Fig. 5E of the revised manuscript). Unlike wild type ACAP1, overexpression of $ACAP1^{R448Q}$ failed to rescue replication of the Δ*bspF* mutant, demonstrating that the GAP activity of ACAP1 is required for the suppressive effect of ACAP1 overexpression on BspF deficiency, further supporting our model of Arf6 inactivation by BspF via ACAP1 GAP activity. This is described in lines 249-258 of the revised manuscript.

Altogether, we now provide four independent lines of evidence that are inconsistent with a scenario of Arf6 hyperactivation, and instead support Arf6 inactivation by BspF.

#3 Co-suppression/reconstitution data in Fig. 3K is not convincing and does not help the author's case regarding the proposed BspF-induced inactivation of Arf6.

> We are unclear as to why this referee finds the suppressive effect of Arf6 depletion on the replication defect of the Δ*bspF* mutant unconvincing. This dataset does not show any weaker or less significant effects of Arf6 depletion on bacterial replication than those of Rab6a/a' or Rab8a

depletions (Fig. 3G-H), which did not seem to be a cause of concern. While our Arf6 depletion dataset does not agree with this referee's alternative model of BspF activity on Arf6, it is consistent with our model of BspF-mediated inactivation of Arf6, which is now further substantiated by our new data on the effect of dominant Arf6 alleles on bacterial replication (Fig. 5D of the revised manuscript).

Minor points:

#1. It may be better to combine Fig. 1 with Fig. 2A for a more substantial opening argument.

> We have now added new experimental data to Fig.1 (revised Fig. 1C), which substantiates the first figure's opening argument. We do not feel that combining Fig. 2A with Fig. 1 would help the flow of the study as the data presented in Fig. 2A is linked to the rest of Fig. 2 and addresses a question distinct from the infection data presented in Fig. 1.

#2. data in Fig. 2b needs to be quantified to report colocalization. Additionally, attachment of tubular structures to vesicles (as described in the text) is highly speculative.

> We have quantified colocalization of mCherry-BspF and GFP-TGN38 signals using the Fiji Coloc_2 plugin to calculate Pearson's correlation coefficients, focusing on select areas containing BspF-positive structures, instead of whole cells, to address whether BspF-labelled structures intersect with the TGN-PM compartment. This quantification shows a colocalization of the two markers that is only slightly decreased upon Cytochalasin D treatment (revised Fig. 2C), confirming intersection between the two compartments. We have revised the description of the data accordingly (lines 119-122 of the revised manuscript) and removed mentions of attached vesicles.

#3. Although the images shown in Fig. 2e are very convincing, quantification in the form of some overlap coefficient derived from multiple cells and at least 2 independent experiments should be provided.

> We thank the referee for this valid suggestion and have now quantified the localization of the different TGN-PM markers tested in both Fig. 2E (now revised Fig. 2F-G) and Fig EV1 to BspF-labelled tubules. Since we sought to characterize host markers that accumulate specifically on BspF-labelled tubules, we did not use an automated image analysis process that would generate correlation coefficient from whole cells but instead quantified the percentage of BspF-expressing cells containing positive tubules, using a stringent cut-off value of 75% of positive tubules/cell analyzed. These quantifications confirmed our original conclusions about the specific accumulation of recycling endosome markers on BspF-labelled tubules.

#4. In my opinion data shown in Figures 4 and 5 should be presented in one figure to improve clarity and flow. Redundant data can be moved to the supplementary to alleviate space constraints.

> We agree with this referee that data presented in Fig 4 and 5 are linked and could be merged, as both address BspF interaction with ACAP1 and its consequences on Arf6 activity. However, in order to address concerns raised by the other referees, the content of both figures has been substantially increased in the revised manuscript and it would now be impossible to merge them into a single, readable figure.

#5. Data on the acquisition of STX6 by the bacterial phagosome are not very convincing. The differences in the images shown are not striking and the quantification is based on manual counting rather than less biased colocalization coefficients. The overall study would benefit from further solidifying this line of observations.

> We regret that this referee did not find the recruitment of Stx6-positive vesicles to rBCVs convincing. The micrographs shown in Fig. 6A illustrate Stx6-positive vesicle recruitment to rBCVs and were not meant to show that rBCV membranes are Stx6-positive, which appears to be the referee's misunderstanding. We observed and quantified recruitment of Stx6-positive, TGN-derived vesicles but did not detect a large accumulation of endogenous Stx6 on rBCV membranes in BMMs. Of note, we observed massive recruitment of overexpressed GFP-STX6 to rBCVs in HeLa cells (see **Figure1A** below, for the referees' and editor's consideration). Out of concern of overexpression artifacts, we considered it more relevant to investigate these membrane acquisition events with endogenous Stx6 in macrophages. We agree with the referee that our original manual method of counting rBCVs associated with Stx6-positive vesicles may be subjective, so we have re-analyzed our raw data using the CellProfiler cell image analysis software. We designed a pipeline (described in new Fig. EV4) that separately identifies individual bacteria - in lieu of rBCVs - and Stx6-positive vesicles and tests in an unbiased manner whether their respective signals overlap, reflecting a vesicle attached or fused to an rBCV. Given the shapes and interactions of these two compartments, a correlation coefficient method is not appropriate. By deriving the pipeline output into percentage of positive rBCVs, we obtained comparably similar results between this analysis and our original method (see **Figure 1B** for the referees' and editor's consideration), ruling out a significant bias in our original analysis. We have now included this automated method of analysis in the revised manuscript to increase confidence in our results.

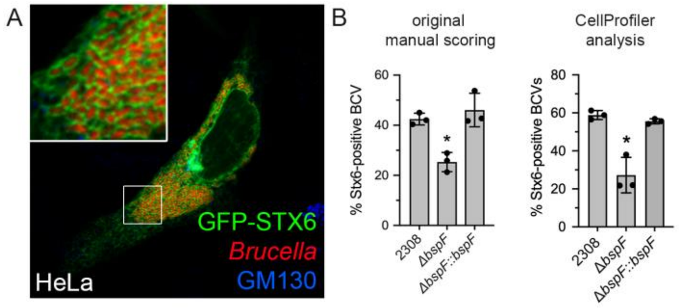

Figure 1: Stx6 recruitment to rBCVs in HeLa cells and BMMs. (A) Representative confocal micrograph of GFP-STX6 recruitment to rBCVs in HeLa cells. (B) Quantification of Stx6-positive vesicle recruitment to rBCVs in BMMs using manual scoring (left panel- original Fig. 6B) or CellProfiler analysis (right panel – revised Fig. 6B).

Referee #3:

In the current manuscript the authors investigate the role of BspF, which has previously been demonstrated to be secreted by Brucella via the type IV secretion system. Previous data from ectopic expression experiments demonstrated that BspF interferes with the host secretory pathway. Here they generated a BspF mutant which is impaired in replication in macrophages. Ectopic expression of BspF demonstrated the targeting of the recycling endosome. They identified in a Yeast Two hybrid screen Arf6 GTPase-activating Protein ACAP1 which interferes with the vesicular transport within the recycling endosome and supports the replication of Brucella in rBCVs.

The subversion of host cell vesicular trafficking by bacterial pathogens is a current topic in the field. The current study would contribute to the mechanistic understanding of these events, however, the results presented and the design of the experiments in the present manuscript do not justify the conclusions drawn by the authors.

> We thank this reviewer for appreciating the relevance of our study and address below their concerns about the validity of our conclusions.

Major points

1. The major problem with this study is that there is no evidence provided that BspF secreted by Brucella really targets the recycling endosome, interacts with ACAP1 and functions by the interference with Arf6/Rab8a-dependent transport. It is definitely easier to use ectopic expression constructs but these overexpression experiments are prone to generate artifacts and at least need to be validated by demonstrating localization of BspF in selected infection experiments (Fig. 2). This is particularly important with ectopically expressed bacterial proteins that are supposed to target intracellular trafficking since they are produced and transported within the compartments they have to target from outside in natural infections.

> We agree with this referee that ectopic expression of bacterial effectors might be prone to overexpression artifacts, or not reflect bacterially delivered effector functions. Ideally, identified phenotypes should be confirmed by direct studies of bacterially delivered effectors. While such direct approaches are feasible with Type III secretion effectors, they have rarely been possible with Type IV secreted effectors, with some exceptions for effectors delivered by Dot/Icm systems. Unfortunately, we have not been able to detect and directly study bacterially delivered BspF via microscopy or biochemical means, a common caveat associated with studying Type IV secretion effectors in *Brucella.* While we indeed could not show that bacterially delivered BspF binds ACAP1, we did show i) that translocated BspF is required for *Brucella*-mediated inhibition of retrograde traffic through the recycling endosome (Fig. 3E), as is ectopically expressed BspF and ii) that BspF-dependent bacterial replication and recruitment of TGN-derived vesicles requires Arf6/Rab8a-dependent transport (Fig. 3H-I, Fig. 6DEH), which is targeted by ectopically expressed BspF. While these results do not constitute direct evidence, we feel that this referee's claim that "there is no evidence provided that BspF secreted by *Brucella* really targets the recycling endosome, …. and functions by the interference with Arf6/Rab8a-dependent transport" is incorrect.

To further address this referee's major concern about our approach, we now provide additional data (revised Fig. 1C and lines 108-110 of the revised manuscript) that shows that ectopically expressed BspF rescues the replication defect of the Δ*bspF* mutant i.e. *in trans* complementation. This demonstrates that ectopically expressed BspF functions as a surrogate for bacterially delivered BspF, which validates our ectopic expression approaches to characterize BspF's target and study its mode of action.

2. Figure 2B and 2C require quantification from different fields and different cells.

> As detailed in response to Referee #2's minor point #2, we now provide quantification of the colocalization between mCherry-BspF and GFP-TGN38 (revised Fig. 2B-C).

3. In figure 3E, CtxB trafficking is demonstrated to be affected as consequence of wildtype infection but not of the bspF mutant. Since the bspF mutant has a growth defect (Fig. 1B) the question is whether the bspB mutant which is used as a control in this experiment has the same growth defect. If not, the control is hardly adequate and should be replaced.

> We thank this referee for pointing out this important detail that we failed to mention in our original manuscript. As we recently published (see Miller *et al*., 2017), a Δ*bspB* mutant displays a replication defect in macrophages that is comparable to that of a Δ*bspF* mutant. It therefore

constitutes an adequate control in the experiment shown in Fig. 3E. This is now clarified (lines 175-176 of the revised manuscript).

4. Experiments in figure 4B need additional controls. For IP experiments an unrelated Bsp protein should be used as negative control. And even more importantly, native complexes should be demonstrated in IPs by pulling down endogenous BspF and ACAP1 from infected cells. The high efficiency of co-IP does not fit at all to the picture of co-localization provided in figure 4C.

> The co-immunoprecipitations presented in Figure 4B were meant as an independent confirmation of the ACAP1-BspF interaction identified by the Yeast-2-hybrid screen data and included a negative control (vector only) for the ACAP1-BspF interaction. We do not feel that an additional effector control would add to these experimental results. As discussed above in response to this referee's main concern, we have yet to detect translocated BspF, which precludes the confirmation of endogenous ACAP1-BspF interactions in infected cells.

The high efficiency of co-IP shown in the original Figure 4B is likely due to the DSP cross-linking step that was performed to visualize ACAP1-BspF interaction, which likely enhanced their interaction. Based on the added quantification of ACAP1-BspF colocalization (see our response to point #5 below), we do not think that our co-IP data is discrepant with the microscopy data presented in Figure 4C. To address this concern, we now provide in the revised Fig. 4B comparative co-IPs in absence or presence of DSP cross-linker that show weak to no interactions between BspF and ACAP1, unless cross-linking is performed. These results suggest a weak or transient interaction between these proteins that may reflect their colocalization pattern, which is now discussed in the manuscript (lines 216-218 of the revised manuscript).

5. Overexpression of GFP-ACAP1 in figure 4 C causes aggregates in the cells to which the mCherry-BspF co-localizes. Since these are the only structures to which co-localization occurs the authors should try to titrate the GFP-ACAP1 to avoid aggregates. Otherwise the co-localization of BspF and ACAP1 occurs in a such a minor compartment compared to the overall expression pattern, that a functional impact of this interaction can be questioned. This interaction in the cell should be quantified in any case.

> The peripheral aggregates seen in Fig. 4C are not specifically caused by expression of GFP-ACAP1, but rather by the Cytochalasin D treatment that was used to enhance/stabilize BspF-labelled tubules. In revised Fig 2B, 2F, EV1 and new Fig. EV3, similar endosomal aggregates (that colocalize to areas of actin aggregation – see Fig. 2B) are visible in cells expressing other host markers and we provide below an example that expression of GFP-ACAP1 and mCherry-BspF in absence of Cytochalasin D treatment does not cause such aggregated structures (Figure 2 for the referee's and editor's consideration). As suggested, we have now quantified the colocalization of GFP-ACAP1 and mCherry-BspF (see revised Fig. 4C and lines 220-224 of the revised manuscript) using a Pearson's correlation coefficient analysis, which shows significant colocalization of these proteins in the cells analyzed.

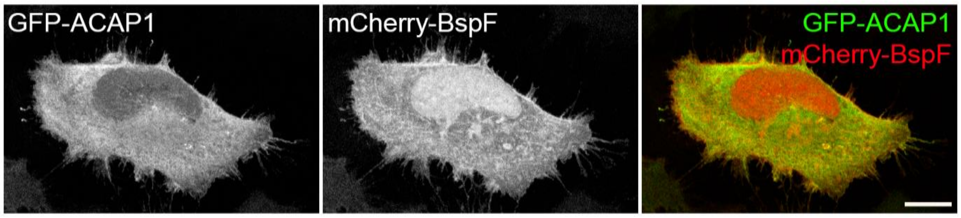

Figure 2. Expression patterns of GFP-ACAP1 and mCherry-BspF in absence of Cytochalasin D treatment. Scale bar, 10 µm.

Minor:
Line 82: What is "aBCV"?

> “aBCV” stands for “autophagic *Brucella*-containing vacuole”, as described in line 74 of the revised manuscript.

Line 97: Explain BMM.

> “BMM” is now fully spelled out as “bone-marrow-derived macrophage” in line 96 of the revised manuscript.

Massive aggregation of STX6: This image should be exchanged for an image that shows less aggregates.

> We assume that this referee refers to the GFP-STX6 micrograph presented in Fig. EV2A. We do not believe that the high STX6 signal seen in the Golgi/TGN area are aggregates, but rather saturated signals due to the need to visualize peripheral, lower intensity tubular structures. Due to the prominent accumulation of STX6 in the Golgi/TGN area, it is very rare to find cells with equivalent perinuclear and peripheral signals and these would not be representative.

1st Revision - Editorial Decision 12th Jul 2021

Thank you for submitting your revised study. The manuscript has been sent back to the original referees and we have now obtained their reports, which are appended below for your information.

As you can see, the referees find that their criticisms have been adequately addressed and recommend the study for publication. Referee #2 and #3 also give you few suggestions to improve the main text.

In addition, there are few editorial issues concerning the text and the figures that I need you to address before we can officially accept your manuscript.

Referee #1:

The authors have done an impressive job responding to my and the other reviewers' initial comments, including further experimental validation and quantification of their observations. The model describing BspF action through binding to ACAP1 and the consequent effect on Arf6 is

plausible and solidly substantiated by several independent lines of experimental evidence. As stated by the authors, it is indeed technically very challenging to detect and document interactions of endogenous type IV-secreted effector proteins in pathogen-infected cells, since Brucella abortus secretes at least 15 (and likely considerably more) effectors at low quantities. The authors have done an excellent job at circumventing and dealing with this technical difficulty.
I have no further suggestions for improvements and believe this insightful and significant study should be given high priority to be published in the EMBO Journal.

Referee #2:

The revised manuscript adequately addresses my major points of criticism by examining the relationship of BspF to Arf6 activation status and ACAP1 catalytic activity using specific point mutants in the context of infection. I now find the revised manuscript suitable for publication.

The following minor comments came up during the reading of the revised manuscript:

Figure 1C - should the last sample also be designated as one * significant? if not, please indicate no significance with 'ns'. The same holds for Figure 3I.

Line 142 - the phrase 'at the RE' is likely extraneous and should be removed.

Referee #3:

The authors responded to all my queries. To my main criticism that the study is based on overexpression of bacterial proteins in eukaryotic cells and at least some of the data should be reproduced with endogenous secreted proteins, the authors replied that it is a common caveat for Type IV secreted effectors in Brucella that they cannot be detected by microscopy in host cells. Although this is hard to understand, The new data demonstrating the complementation of the growth defect of the ΔbspF mutant by expressing GFP-BspF in macrophages is interesting and indeed points to role of BspF in the macrophage. But why did they not use the mCherry-BspF construct for complementation of the mutant which was used in many other experiments in the manuscript? The GFP-BspF is not characterized in this paper and just used for this complementation experiment. They should at least mention that the mCherry-BspF and the GFP-BspF behaved the same in the localization studies.

In figure 1C, the ΔbspF::bspF + GFP-BspF doesn't appear to be significant. Why is that?

EMBOJ-2021-107664R - Responses to referees' comments

Referee #1:

The authors have done an impressive job responding to my and the other reviewers' initial comments, including further experimental validation and quantification of their observations. The model describing BspF action through binding to ACAP1 and the consequent effect on Arf6 is plausible and solidly substantiated by several independent lines of experimental evidence. As stated by the authors, it is indeed technically very challenging to detect and document interactions of endogenous type IV-secreted effector proteins in pathogen-infected cells, since Brucella abortus secretes at least 15 (and likely considerably more) effectors at low quantities. The authors have done an excellent job at circumventing and dealing with this technical difficulty.
I have no further suggestions for improvements and believe this insightful and significant study should be given high priority to be published in the EMBO Journal.

> We thank this referee for their enthusiastic assessment of our revised manuscript and for appreciating our efforts to address all original concerns.

Referee #2:

The revised manuscript adequately addresses my major points of criticism by examining the relationship of BspF to Arf6 activation status and ACAP1 catalytic activity using specific point mutants in the context of infection. I now find the revised manuscript suitable for publication.

The following minor comments came up during the reading of the revised manuscript:

Figure 1C - should the last sample also be designated as one * significant? if not, please indicate no significance with 'ns'. The same holds for Figure 3I.

> We apologize for the confusion with how we marked significant different experimental groups in Figures 1C and 3I. We tried to highlight the key statistical differences between groups in these panels and did not indicate all results, which we agree is misleading. We have now edited both panels to show all significant differences.

Line 142 - the phrase 'at the RE' is likely extraneous and should be removed.

> We have now edited this sentence accordingly.

Referee #3:

The authors responded to all my queries. To my main criticism that the study is based on overexpression of bacterial proteins in eukaryotic cells and at least some of the data should be reproduced with endogenous secreted proteins, the authors replied that it is a common caveat for Type IV secreted effectors in Brucella that they cannot be detected by microscopy in host cells. Although this is hard to understand, The new data demonstrating the complementation of the growth defect of the ΔbspF mutant by expressing GFP-BspF in macrophages is interesting and indeed points to role of BspF in the macrophage. But why did they not use the mCherry-BspF construct for complementation of the mutant which was used in many other experiments in the manuscript? The GFP-BspF is not characterized in this paper and just used for this

complementation experiment. They should at least mention that the mCherry-BspF and the GFP-BspF behaved the same in the localization studies.

> The *Brucella* strains we used in this study express the red fluorescent protein $DsRed_m$ for fluorescence microscopy detection purposes, which is excited at the same wavelengths as mCherry. We therefore could not use mCherry-BspF to *trans*-complement the Δ*bspF* mutant in macrophages and had to use GFP-BspF instead. We now provide in the revised Fig. EV1A evidence that mCherry-BspF and GFP-BspF co-localize to the same tubular structures in Cytochalasin D-treated HeLa cells when co-expressed (see also lines 122-123 of the revised manuscript), arguing that the fluorescent tag used does not influence BspF function.

In figure 1C, the ΔbspF::bspF + GFP-BspF doesn't appear to be significant. Why is that?

> We apologize for the confusion with how we marked significantly different experimental groups in Figures 1C, as discussed above in response to Referee #2's comment. We have edited Fig. 1C panel to clarify this point.

2nd Revision - Editorial Decision 30th Jul 2021

I am pleased to inform you that your manuscript has been accepted for publication in The EMBO Journal.

EMBO PRESS

YOU MUST COMPLETE ALL CELLS WITH A PINK BACKGROUND ➔

PLEASE NOTE THAT THIS CHECKLIST WILL BE PUBLISHED ALONGSIDE YOUR PAPER

Corresponding Author Name: Jean Celli
Journal Submitted to: The EMBO Journal
Manuscript Number: EMBOJ-2021-107664

Reporting Checklist For Life Sciences Articles (Rev. June 2017)

This checklist is used to ensure good reporting standards and to improve the reproducibility of published results. These guidelines are consistent with the Principles and Guidelines for Reporting Preclinical Research issued by the NIH in 2014. Please follow the journal's authorship guidelines in preparing your manuscript.

A- Figures
1. Data
The data shown in figures should satisfy the following conditions:

- the data were obtained and processed according to the field's best practice and are presented to reflect the results of the experiments in an accurate and unbiased manner.
- figure panels include only data points, measurements or observations that can be compared to each other in a scientifically meaningful way.
- graphs include clearly labeled error bars for independent experiments and sample sizes. Unless justified, error bars should not be shown for technical replicates.
- if n< 5, the individual data points from each experiment should be plotted and any statistical test employed should be justified
- Source Data should be included to report the data underlying graphs. Please follow the guidelines set out in the author ship guidelines on Data Presentation.

2. Captions

Each figure caption should contain the following information, for each panel where they are relevant:

- a specification of the experimental system investigated (eg cell line, species name).
- the assay(s) and method(s) used to carry out the reported observations and measurements
- an explicit mention of the biological and chemical entity(ies) that are being measured.
- an explicit mention of the biological and chemical entity(ies) that are altered/varied/perturbed in a controlled manner.

- the exact sample size (n) for each experimental group/condition, given as a number, not a range;
- a description of the sample collection allowing the reader to understand whether the samples represent technical or biological replicates (including how many animals, litters, cultures, etc.).
- a statement of how many times the experiment shown was independently replicated in the laboratory.
- definitions of statistical methods and measures:
  - common tests, such as t-test (please specify whether paired vs. unpaired), simple χ2 tests, Wilcoxon and Mann-Whitney tests, can be unambiguously identified by name only, but more complex techniques should be described in the methods section;
  - are tests one-sided or two-sided?
  - are there adjustments for multiple comparisons?
  - exact statistical test results, e.g., P values = x but not P values < x;
  - definition of 'center values' as median or average;
  - definition of error bars as s.d. or s.e.m.

Any descriptions too long for the figure legend should be included in the methods section and/or with the source data.

In the pink boxes below, please ensure that the answers to the following questions are reported in the manuscript itself. Every question should be answered. If the question is not relevant to your research, please write NA (non applicable). We encourage you to include a specific subsection in the methods section for statistics, reagents, animal models and human subjects.

USEFUL LINKS FOR COMPLETING THIS FORM

http://www.antibodypedia.com
http://1degreebio.org
http://www.equator-network.org/reporting-guidelines/improving-bioscience-research-repor

http://grants.nih.gov/grants/olaw/olaw.htm
http://www.mrc.ac.uk/Ourresearch/Ethicsresearchguidance/Useofanimals/index.htm
http://ClinicalTrials.gov
http://www.consort-statement.org
http://www.consort-statement.org/checklists/view/32-consort/66-title

http://www.equator-network.org/reporting-guidelines/reporting-recommendations-for-tumo

http://datadryad.org

http://figshare.com

http://www.ncbi.nlm.nih.gov/gap

http://www.ebi.ac.uk/ega

http://biomodels.net/

http://biomodels.net/miriam/
http://jjj.biochem.sun.ac.za
http://oba.od.nih.gov/biosecurity/biosecurity_documents.html
http://www.selectagents.gov/

B- Statistics and general methods

Please fill out these boxes ➔ (Do not worry if you cannot see all your text once you press return)

1.a. How was the sample size chosen to ensure adequate power to detect a pre-specified effect size?	NA
1.b. For animal studies, include a statement about sample size estimate even if no statistical methods were used.	NA
2. Describe inclusion/exclusion criteria if samples or animals were excluded from the analysis. Were the criteria pre-established?	NA
3. Were any steps taken to minimize the effects of subjective bias when allocating animals/samples to treatment (e.g. randomization procedure)? If yes, please describe.	NA
For animal studies, include a statement about randomization even if no randomization was used.	NA
4.a. Were any steps taken to minimize the effects of subjective bias during group allocation or/and when assessing results (e.g. blinding of the investigator)? If yes please describe.	All microscopy-based analyses of samples were performed blind without knowledge of the sample identity. Some experiments were repeated by different operators to ensure that no subjective bias affected data analysis. The cell image analysis software CellProfiler was used to provide automated analysis of microscopy data.
4.b. For animal studies, include a statement about blinding even if no blinding was done	NA
5. For every figure, are statistical tests justified as appropriate?	Yes, statistical tests to be applied to all data sets were defined by the experimental design and justified as appropriate using GraphPad Prism 8 and 9.
Do the data meet the assumptions of the tests (e.g., normal distribution)? Describe any methods used to assess it.	Yes. Statistical analysis tests used unpaired Student's two-tailed t-tests, Mann-Whitney test, and one- or two-way analysis of variance (ANOVA) with Sidak's, Dunnett's, or Tukey's Multiple comparison tests.

Is there an estimate of variation within each group of data?	Yes, standard deviation is reported in all experimental analyses.
Is the variance similar between the groups that are being statistically compared?	Yes.

C- Reagents

6. To show that antibodies were profiled for use in the system under study (assay and species), provide a citation, catalog number and/or clone number, supplementary information or reference to an antibody validation profile. e.g., Antibodypedia (see link list at top right), 1DegreeBio (see link list at top right).	All antibodies against endogenous proteins used in this study were either tested in the lab for specificity via siRNA-mediated depletion, or have been validated as labelling their known intracellular compartment by immunofluorescence. All catalog numbers and RRID numbers are provided in the Reagents and Tools Table.
7. Identify the source of cell lines and report if they were recently authenticated (e.g., by STR profiling) and tested for mycoplasma contamination.	Human embryonic kidney 293T cells (HEK293T/17; ATCC CRL-11268); HeLa cells (ATCC clone CCL-2). All cell lines are routinely tested for mycoplasma contamination in the laboratory.

* for all hyperlinks, please see the table at the top right of the document

D- Animal Models

8. Report species, strain, gender, age of animals and genetic modification status where applicable. Please detail housing and husbandry conditions and the source of animals.	6-12 week-old C75BL/6J female mice purchased from Jackson Laboratories were used to generate bone marrow derived macrophages. Mice were housed and cared in an AAALAC-certified vivarium facility following housing and husbandry procedures established and approved by the Washington State University Institutional Animal Care and Use Committee (IACUC).
9. For experiments involving live vertebrates, include a statement of compliance with ethical regulations and identify the committee(s) approving the experiments.	NA
10. We recommend consulting the ARRIVE guidelines (see link list at top right) (PLoS Biol. 8(6), e1000412, 2010) to ensure that other relevant aspects of animal studies are adequately reported. See author guidelines, under 'Reporting Guidelines'. See also: NIH (see link list at top right) and MRC (see link list at top right) recommendations. Please confirm compliance.	NA

E- Human Subjects

11. Identify the committee(s) approving the study protocol.	NA
12. Include a statement confirming that informed consent was obtained from all subjects and that the experiments conformed to the principles set out in the WMA Declaration of Helsinki and the Department of Health and Human Services Belmont Report.	NA
13. For publication of patient photos, include a statement confirming that consent to publish was obtained.	NA
14. Report any restrictions on the availability (and/or on the use) of human data or samples.	NA
15. Report the clinical trial registration number (at ClinicalTrials.gov or equivalent), where applicable.	NA
16. For phase II and III randomized controlled trials, please refer to the CONSORT flow diagram (see link list at top right) and submit the CONSORT checklist (see link list at top right) with your submission. See author guidelines, under 'Reporting Guidelines'. Please confirm you have submitted this list.	NA
17. For tumor marker prognostic studies, we recommend that you follow the REMARK reporting guidelines (see link list at top right). See author guidelines, under 'Reporting Guidelines'. Please confirm you have followed these guidelines.	NA

F- Data Accessibility

18: Provide a "Data Availability" section at the end of the Materials & Methods, listing the accession codes for data generated in this study and deposited in a public database (e.g. RNA-Seq data: Gene Expression Omnibus GSE39462, Proteomics data: PRIDE PXD000208 etc.) Please refer to our author guidelines for 'Data Deposition'.<br><br>Data deposition in a public repository is mandatory for:<br>a. Protein, DNA and RNA sequences<br>b. Macromolecular structures<br>c. Crystallographic data for small molecules<br>d. Functional genomics data<br>e. Proteomics and molecular interactions	This study includes no data deposited in external repositories.
19. Deposition is strongly recommended for any datasets that are central and integral to the study; please consider the journal's data policy. If no structured public repository exists for a given data type, we encourage the provision of datasets in the manuscript as a Supplementary Document (see author guidelines under 'Expanded View' or in unstructured repositories such as Dryad (see link list at top right) or Figshare (see link list at top right).	NA
20. Access to human clinical and genomic datasets should be provided with as few restrictions as possible while respecting ethical obligations to the patients and relevant medical and legal issues. If practically possible and compatible with the individual consent agreement used in the study, such data should be deposited in one of the major public access-controlled repositories such as dbGAP (see link list at top right) or EGA (see link list at top right).	NA
21. Computational models that are central and integral to a study should be shared without restrictions and provided in a machine-readable form. The relevant accession numbers or links should be provided. When possible, standardized format (SBML, CellML) should be used instead of scripts (e.g. MATLAB). Authors are strongly encouraged to follow the MIRIAM guidelines (see link list at top right) and deposit their model in a public database such as Biomodels (see link list at top right) or JWS Online (see link list at top right). If computer source code is provided with the paper, it should be deposited in a public repository or included in supplementary information.	NA

G- Dual use research of concern

22. Could your study fall under dual use research restrictions? Please check biosecurity documents (see link list at top right) and list of select agents and toxins (APHIS/CDC) (see link list at top right). According to our biosecurity guidelines, provide a statement only if it could.	This study includes the use of Select Agents (Brucella abortus) that has been reviewed and approved by the CDC/USDA Federal Select Agent Program and does not fall under dual use research restrictions.
